# Formal Carbene Insertion into Cyclopropanones: Access to 2-Aroyl Cyclobutanones via Sulfonium Ylides

**DOI:** 10.1021/acs.joc.5c00167

**Published:** 2025-04-29

**Authors:** Ishika Agrawal, Marvin Lange, Arthur Semmelmaier, Heinrich F. von Köller, Daniel B. Werz

**Affiliations:** Albert-Ludwigs-Universität Freiburg, Institute of Organic Chemistry, Albertstr. 21, 79104 Freiburg, Germany

## Abstract

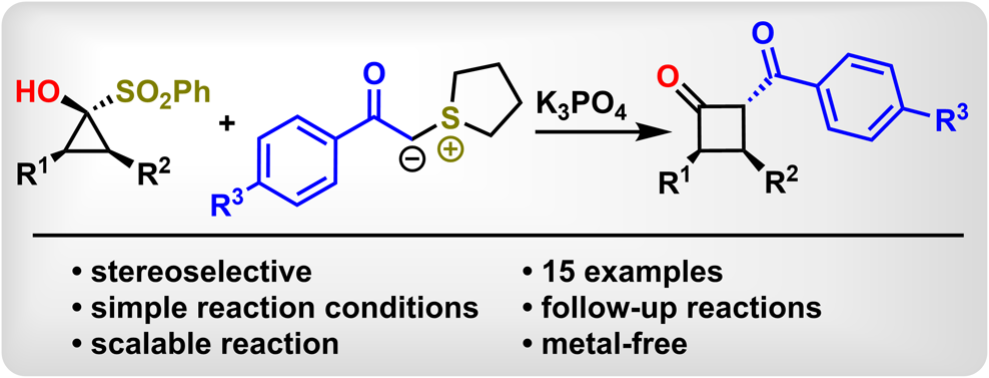

This report presents a method for the synthesis of 2-aroyl cyclobutanones via the reaction of in situ-generated cyclopropanones with acyl sulfonium ylides representing a formal carbene insertion into cyclopropanones. The reaction is highly stereoselective in the case of 2-substituted cyclopropanones, and the cyclobutanones thus obtained are well suited to α-alkylation, offering versatile synthetic applications.

## Introduction

**Scheme 1:**
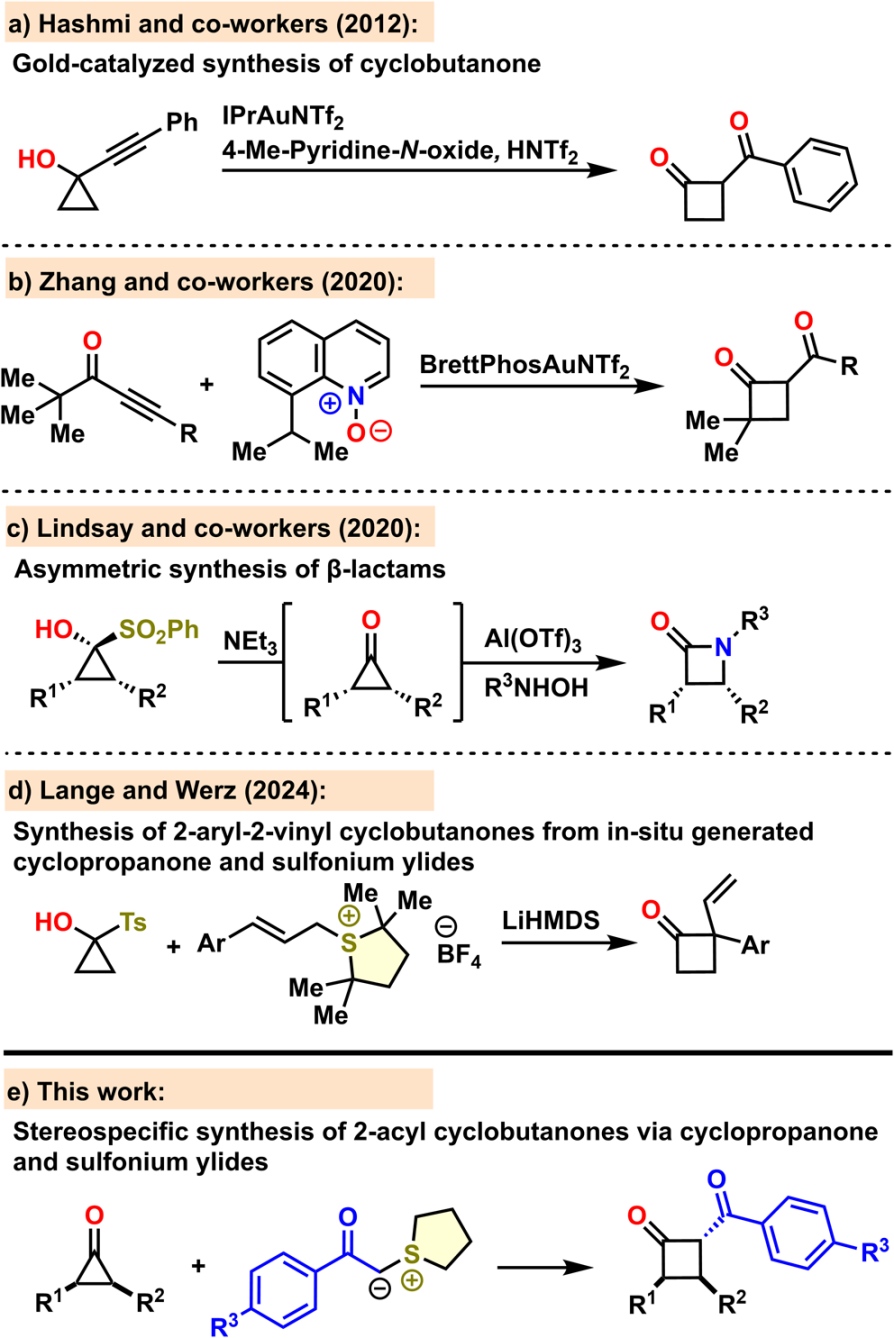
Previous Work and Our Design

Cyclobutanones are highly valuable intermediates in organic synthesis.^1^ Additionally, cyclobutanes serve as crucial building blocks in synthetic chemistry due to their high ring strain. Moreover, these structures are frequently encountered as key motifs in bioactive natural products and pharmaceuticals.^2,3^ Traditionally, cyclobutanones have been prepared via [2 + 2]-cycloaddition reactions of ketenes and olefins or enol ethers.^4,5^ Over the years, significant advancements have been made using different metal catalysts to enhance efficiency and selectivity.^6^ For instance, gold,^7^ ruthenium,^8^ and palladium^9^ catalysts have been widely used for cyclobutanone synthesis through semipinacol-type ring-expansion reactions of cyclopropanols.^10^ In 2012, Hashmi and co-workers demonstrated a gold-catalyzed oxidative rearrangement of propargyl alcohols to 1,3-diketones (Scheme 1a).^11^ Later, in 2020 Zhang and co-workers reported the C–H insertion of an oxidatively generated gold carbene with *tert*-butyl alkynyl ketones, leading to the formation of strained cyclobutanones (Scheme 1b).^12^

Notably, Lindsay and co-workers established the viability of enantioenriched 1-sulfonylcyclopropanols (SCPs) for synthesizing chiral β-lactams through a stereospecific [1,2]-shift involving a hydroxylamine hemiaminal intermediate (Scheme 1c).^13^ Recently, our group reported a straightforward and efficient one-pot procedure for synthesizing various 2-aryl-2-vinyl-substituted cyclobutanones. This approach makes use of readily available 1-tosylcyclopropanol as a cyclopropanone precursor and cinnamyl sulfonium salts as starting materials (Scheme 1d). The process involves the nucleophilic attack of an in situ-formed sulfonium ylide on the electrophilic carbonyl of the cyclopropanone, which ultimately, after a series of proton shifts, leads to the formation of a leaving group and triggers the subsequent ring-expansion.^14^ Building on this strategy, we hypothesized that a similar approach could allow a one-pot synthesis of 2-aroylcyclobutanones from SCPs and acyl sulfonium salts as starting materials (Scheme 1e).

## Results and Discussion

We began our investigations employing the literature-known benzoyl sulfonium bromide salt resulting from the reaction of bromoacetophenone and thiolane as the precursor to sulfonium ylide **2**.^15^ However, when we reacted SCP **1a** with this sulfonium salt in the presence of different bases, the reaction primarily led to an undesired product resulting from the nucleophilic substitution of thiolane by the sulfinate anion generated in the cyclopropanone formation. To overcome this issue, we modified the reaction by directly utilizing sulfonium ylide **2a** which was obtained by reacting the benzoyl sulfonium salt with aqueous NaOH. Notably, the stability of the obtained sulfonium ylides strongly depends on the substituent at the arene core. Therefore, the sulfonium ylides were directly submitted to the cyclobutanone synthesis without further purification. This refined approach enabled the subsequent reaction with SCP **1a**, promoting the formation of the 2-benzoyl cyclobutanone **3a**.

**Scheme 2:**
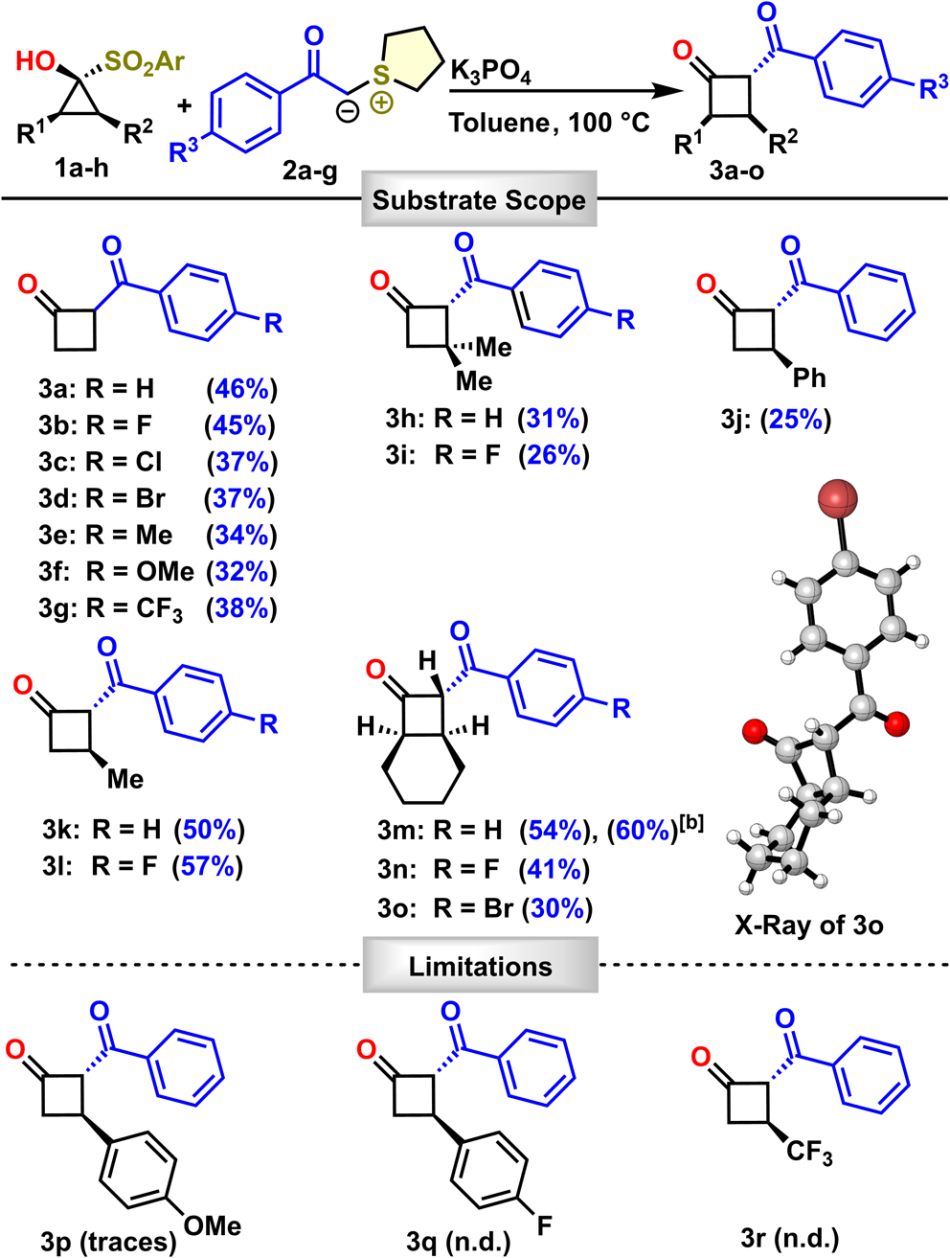
Cyclobutanone Substrate Scope Isolated yields on a 0.3 mmol scale if not stated otherwise. Reaction performed on a 1 mmol scale.

Following a thorough optimization of the reaction conditions (see Supporting Information for detailed information), we observed that strong bases such as LiHMDS or KOH (Table 1, entries 1 and 2) were not suitable to facilitate the desired reaction. Instead, K_3_PO_4_ proved to be more effective in promoting product formation (entry 3). Further investigations into the reaction parameters revealed that both the choice of solvent (entries 3 to 5) and the reaction temperature (entries 5 to 9) played critical roles in achieving a successful transformation. Toluene emerged as the optimal solvent, while low concentration of SCP and elevated temperatures were essential for maximizing efficiency. Based on these findings, we conducted the reaction using K_3_PO_4_ as the base and toluene as the solvent at 100 °C. Notably, within 30 min complete conversion of **1a** was confirmed by GC/MS analysis, leading to 46% isolated yield of **3a**. The resulting cyclobutanones were found to be moisture-sensitive and exhibited instability under humid conditions at room temperature, leading to ring-opening into the corresponding keto carboxylic acid over time (see SI). Storage under an atmosphere of argon at −20 °C significantly helped to increase stability. With optimized reaction conditions in hand, we examined the substrate scope of the transformation (Scheme 2). When reacting unsubstituted SCP **1a** with various 4-aryl-substituted sulfonium ylides **2a**–**g**, 2-aroylcyclobutanones **3a**–**g** were obtained in moderate yields. The reaction demonstrated tolerance for both electron-withdrawing and electron-donating groups.

**Table 1:**
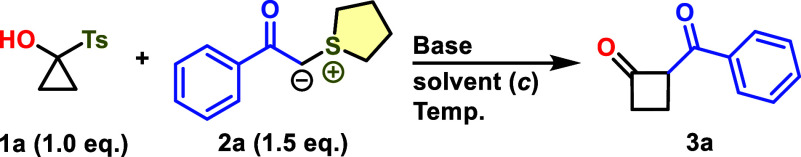
Optimization Table of the Reaction Conditions^a^

entry^b^	solvent (c/M)^c^	base (eq.)	*T* [°C]	yield [%]^d^^,^^e^
1	CH_2_Cl_2_ (0.05)	LiHMDS (1.0)	–78 to r.t.	<5
2	CH_2_Cl_2_ (0.05)	KOH (1.0)	–78 to r.t.	–
3	CH_2_Cl_2_ (0.05)	K_3_PO_4_ (1.0)	–78 to r.t.	13
4	DME (0.05)	K_3_PO_4_ (1.5)	–78 to r.t.	17
5	PhMe (0.05)	K_3_PO_4_ (1.5)	–78 to r.t.	23
6	PhMe (0.01)	K_3_PO_4_ (1.5)	0 to r.t.	24
7	PhMe (0.01)	K_3_PO_4_ (1.5)	r.t.	19
8	PhMe (0.03)	K_3_PO_4_ (1.5)	100	52 (46)
9	PhMe (0.01)	K_3_PO_4_ (1.5)	50	22

a See Supporting Information (SI) for detailed information.

b Reactions were carried out on a 0.1 mmol scale with respect to the SCP **1a**.

c Concentration with respect to SCP **1a**.

d Yields refer to ^1^H NMR yield against a 1,3,5-trimethoxybenzene standard.

e Yields in parentheses refer to isolated and purified products on a 0.3 mmol scale.

Next, we turned our attention to preparing and utilizing several substituted SCPs. Reacting the geminal dimethyl-substituted SCP **1b** with sulfonium ylides **2a** and **2b** afforded the cyclobutanones **3h** and **3i** in 31 and 26% yield, respectively. When phenyl-substituted SCP **1c** was reacted with sulfonium ylide **2a** 2,3-disubstituted cyclobutanone **3j** was isolated in 25% yield as the sole cyclobutanone product of the reaction. When methyl-substituted SCP **1d** was reacted with sulfonium ylides **2a** and **2b** 2,3-disubstituted cyclobutanones **3k** and **3l** were isolated in 50 and 57% yields, respectively. Careful NMR analyses of our products, as well as X-ray diffraction analysis of cyclobutanone **3o** confirmed that the *trans*-substituted cyclobutanones were obtained. Furthermore, when enantiomerically pure SCP **1d** (>99% *ee*) was used in the reaction with sulfonium ylide **2a**, cyclobutanone **3k** was obtained (92% *ee*) with only slight loss of enantiopurity confirming the stereospecificity of the reaction (see Supporting Information for further information). Gratifyingly, the fused SCP **1e** demonstrated good reactivity with sulfonium ylides **2a**, **2b** and **2d** producing **3m**, **3n** and **3o** in 54, 41 and 30% yield, respectively. In addition, our method shows scalability. Reacting the fused SCP **1e** and sulfonium ylide **2a** on a 1 mmol scale yielded cyclobutanone **3m** in 60% yield.

**Scheme 3:**
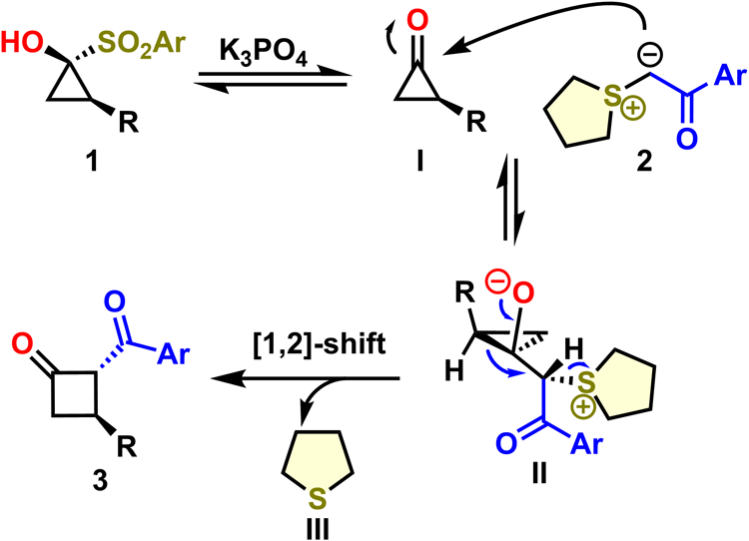
Plausible Mechanism

Based on our observations, the following mechanistic scenario is proposed (Scheme 3). Upon formation of cyclopropanone **I** from the SCP **1** under basic conditions, ylide **2** preferentially attacks the carbonyl of cyclopropanone **I** from the opposite face with respect to residue R, forming cyclopropoxide intermediate **II**. Subsequently, elimination of thiolane **III** facilitates ring-expansion via a stereospecific [1,2]-shift to afford the *trans*-substituted cyclobutanone **3**.

Notably, no other regioisomeric cyclobutanones were observed as the carbon center with higher electron density migrates similar to Baeyer–Villiger rearrangements, i.e., in our case the higher substituted bond.^13a,13d,13e^ This is easily understandable in terms of MO theory since the positive inductive effect of the substituents raises the energy level of the respective filled σ orbital.

**Figure 1:**
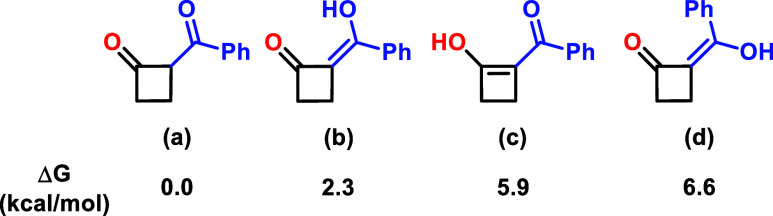
Different enol forms and their energies (M062X/def2-QZVP, D3, CPCM(chloroform)).

When measuring the NMR spectra of these compounds we were surprised that no enol forms were observed as it is most commonly the case in 1,3-dicarbonyl compounds. Theoretically, two enol forms might be possible (Figure 1). Both of them would involve a further sp^2^-hybridized carbon in the four-membered ring. The formation of such structures would increase the strain energy of the four-membered rings tremendously. Simple density functional theory (DFT) studies (M062X/def2-QZVP, D3, CPCM (chloroform)) we performed showed that (b) and (c) are +2.3 and +5.9 kcal/mol higher in energy then the 1,3-diketone (a). Consequently, the equilibrium between the ketone and the enol is strongly in favor of the ketone. More precisely, only 2% enol formation is expected.

**Scheme 4:**
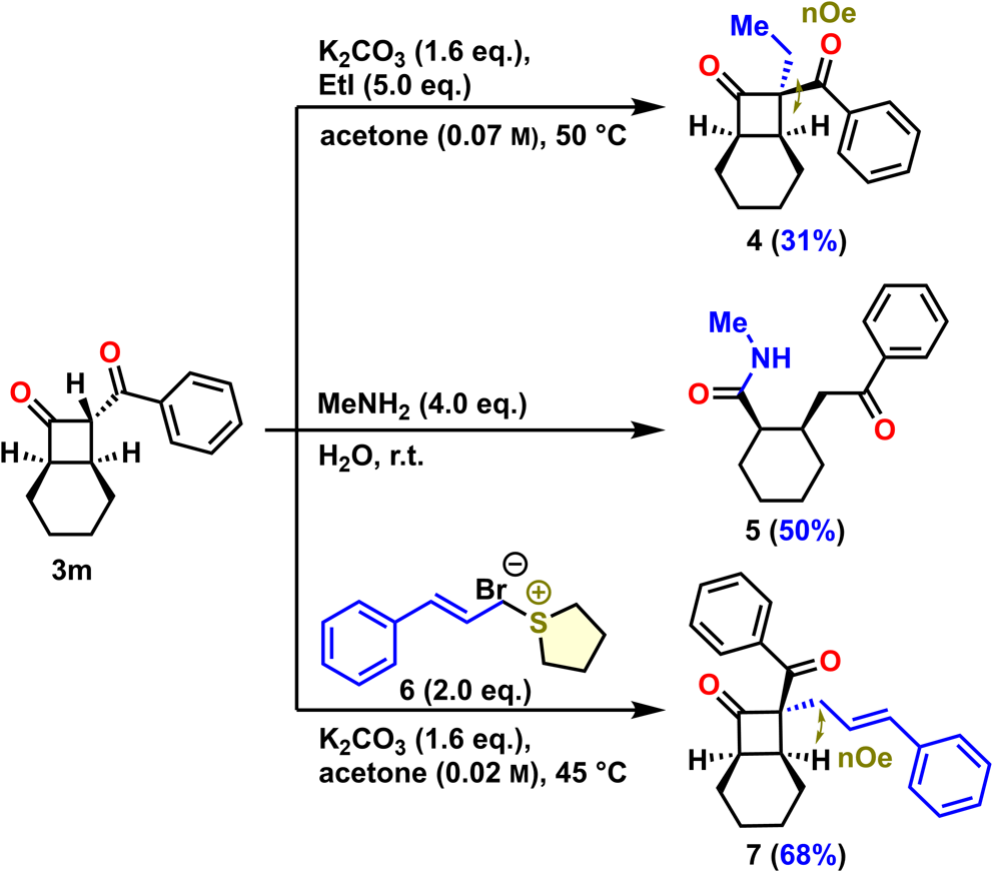
Follow-up Reactions

Cyclobutanone **3m** has emerged as a valuable substrate for alkylation, facilitating the formation of all-carbon quaternary centers.^16^ Thus, we performed several transformations on the cyclobutanones obtained in this study (Scheme 4). Alkylation of **3m** in the presence of K_2_CO_3_ and ethyl iodide diastereoselectively gave ethyl-substituted cyclobutanone **4** in 31% yield. Attack took place from the convex less hindered side of the molecule as it was proven by NOE investigations. Reaction of **3m** with methylamine led to ring-opening, producing cyclohexane **5** in 50% yield. Allylation of **3m** with the cinnamyl sulfonium salt **6**(14) diastereoselectively afforded cyclobutanone **7** in 68% yield. The configuration of the quaternary stereogenic center was confirmed again by NOE correlation of the allylic protons with the bridgehead protons of the fused ring system.

## Conclusions

An effective method for the stereoselective synthesis of *trans*-2,3-disubstituted aroyl cyclobutanones is reported via the insertion of sulfonium ylides into cyclopropanones which were generated in situ from stable SCP precursors. This protocol is characterized by mild reaction conditions and moderate functional group tolerance. Additionally, alkyl-substituted cyclobutanones were synthesized with ease and efficiency.

## Experimental Section

### General Procedures (GP1) for the Synthesis of Sulfonium Ylides **2a**–**2g**

Sulfonium ylides were synthesized following the procedure reported by Zefirov and co-workers.^15^ A solution of tetrahydrothiophene (132 mg, 1.50 mmol, 0.13 mL, 1.00 equiv) and the respective acyl bromide (1.50 mmol, 1.00 equiv) in anhydrous acetonitrile (0.35 M, 4 mL) under inert atmosphere (argon) was stirred at r.t. for 48 h. The resulting reaction mixture was then filtered and the solid residue was washed with diethyl ether and dried under vacuum affording the sulfonium salts as white solids. Subsequently, an aqueous solution of sodium hydroxide (1 M, 1.65 mmol, 1.65 mL, 1.10 equiv) was added dropwise to a stirred suspension of the crude sulfonium salt in H_2_O (5 mL) at 0 °C. The reaction mixture was allowed to stir at r.t. for 12 h and then extracted with CHCl_3_ (2 × 15 mL). The combined organic phases were dried over anhydrous MgSO_4_, filtered and concentrated under reduced pressure to yield the respective sulfonium ylides **2a**–**2g**.

#### 1-Phenyl-2-(tetrahydro-1λ^4^-thien-1-ylidene)ethan-1-one **(2a)**

Prepared according to **GP1** from 2-bromo-1-phenylethan-1-one (299 mg) afforded sulfonium ylide **2a** (220 mg, 1.07 mmol, 71%) as a pale-yellow solid. m.p.: 108–109 °C. FTIR (ATR):  [cm^–1^] = 2944, 1664, 1590, 1559, 1509, 1478, 1446, 1394, 1330, 1308, 1274, 1214, 1175, 1096, 1068, 1020, 1001. ^1^H NMR (400 MHz, CDCl_3_): δ (ppm) = 7.81–7.74 (m, 2H), 7.37–7.30 (m, 3H), 4.33 (s, 1H), 3.68–3.52 (m, 2H), 3.19–2.98 (m, 2H), 2.84–2.59 (m, 2H), 2.06–1.91 (m, 2H). ^13^C{^1^H} NMR (101 MHz, CDCl_3_): δ (ppm) = 181.7, 140.8, 129.3, 127.7, 126.2, 51.5, 43.1, 28.5. HRMS (ESI, Orbitrap): C_12_H_15_OS, calcd.: 207.0838, found: 207.0835 [M + H]^+^.

#### 1-(4-Fluorophenyl)-2-(tetrahydro-1λ^4^-thien-1-ylidene)ethan-1-one **(2b)**

Prepared according to **GP1** from 2-bromo-1-(4-fluorophenyl)ethanone (326 mg) afforded sulfonium ylide **2b** (202 mg, 0.90 mmol, 60%) as a yellow solid. m.p.: 76–77 °C. FTIR (ATR):  [cm^–1^] = 2937, 1669, 1597, 1513, 1497, 1385, 1210, 1153, 1083, 1012. ^1^H NMR (500 MHz, CDCl_3_): δ (ppm) = 7.82–7.70 (m, 2H), 7.04–6.95 (m, 2H), 4.26 (s, 1H), 3.66–3.54 (m, 2H), 3.09 (dddd, *J* = 11.3, 6.2, 5.1, 1.3 Hz, 2H), 2.82–2.66 (m, 2H), 2.06–1.92 (m, 2H). ^13^C{^1^H} NMR (126 MHz, CDCl_3_): δ (ppm) = 180.4, 163.6 (d, *J* = 248.3 Hz), 137.0 (d, *J* = 3.1 Hz), 128.2 (d, *J* = 8.4 Hz), 114.5 (d, *J* = 21.3 Hz), 51.5, 43.1, 28.6. ^19^F NMR (471 MHz, CDCl_3_): δ (ppm) = – 112.3 (m). HRMS (ESI, Orbitrap): C_12_H_14_OFS, calcd.: 225.0744, found: 225.0740 [M + H]^+^.

#### 1-(4-Chlorophenyl)-2-(tetrahydro-1λ^4^-thien-1-ylidene)ethan-1-one **(2c)**

Prepared according to **GP1** from 2-bromo-1-(4-chlorophenyl)ethanone (350 mg) afforded sulfonium ylide **2c** (223 mg, 0.93 mmol, 62%) as a red oil. FTIR (ATR):  [cm^–1^] = 2941, 1915, 1670, 1577, 1506, 1483, 1399, 1379, 1308, 1274, 1202, 1172, 1133, 1086, 1011. ^1^H NMR (400 MHz, CDCl_3_): δ (ppm) = 7.74–7.68 (m, 2H), 7.32–7.27 (m, 2H), 4.32 (s, 1H), 3.76–3.46 (m, 2H), 3.16–2.98 (m, 2H), 2.87–2.67 (m, 2H), 2.05–1.93 (m, 2H). ^13^C{^1^H} NMR (101 MHz, CDCl_3_): δ (ppm) = 180.9, 139.8, 135.8, 128.5, 128.3, 52.7, 43.7, 29.2. HRMS (ESI, Orbitrap): C_12_H_14_OClS, calcd.: 241.0459, found: 241.0456 [M + H]^+^.

#### 1-(4-Bromophenyl)-2-(tetrahydro-1λ^4^-thien-1-ylidene)ethan-1-one **(2d)**

Prepared according to **GP1** from 2-bromo-1-(4-bromophenyl)ethanone (417 mg) afforded sulfonium ylide **2d** (297 mg, 1.05 mmol, 70%) as a red oil. FTIR (ATR):  [cm^–1^] = 2942, 1916, 1671, 1576, 1511, 1480, 1396, 1375, 1308, 1294, 1272, 1201, 1173, 1133, 1083, 1067, 1007. ^1^H NMR (400 MHz, CDCl_3_): δ (ppm) = 7.66–7.62 (m, 2H), 7.48–7.44 (m, 2H), 4.30 (s, 1H), 3.71–3.53 (m, 2H), 3.17–3.04 (m, 2H), 2.82–2.68 (m, 2H), 2.06–1.95 (m, 2H). ^13^C{^1^H} NMR (101 MHz, CDCl_3_): δ (ppm) = 181.0, 140.3, 131.5, 128.6, 124.2, 52.7, 43.7, 29.2. HRMS (ESI, Orbitrap): C_12_H_14_OBrS, calcd.: 284.9949, found: 284.9945 [M + H]^+^.

#### 2-(Tetrahydro-1λ^4^-thien-1-ylidene)-1-(*p*-tolyl)ethan-1-one **(2e)**

Prepared according to **GP1** from 2-bromo-1-(4-methylphenyl)ethanone (320 mg) afforded sulfonium ylide **2e** (284 mg, 1.29 mmol, 86%) as a red oil. FTIR (ATR):  [cm^–1^] = 2943, 1664, 1589, 1558, 1509, 1478, 1393, 1329, 1307, 1273, 1174, 1096, 1020, 1001. ^1^H NMR (500 MHz, CDCl_3_): δ (ppm) = 7.68 (d, *J* = 8.1 Hz, 2H), 7.13 (d, *J* = 7.9 Hz, 2H), 4.30 (s, 1H), 3.60 (dtd, *J* = 11.6, 6.4, 3.0 Hz, 2H), 3.14 – 3.02 (m, 2H), 2.80 – 2.69 (m, 2H), 2.34 (s, 3H), 1.98 (m, 2H). ^13^C{^1^H} NMR (126 MHz, CDCl_3_): δ (ppm) = 181.9, 139.6, 138.2, 128.7, 126.5, 51.5, 43.4, 28.8, 21.4. HRMS (ESI, Orbitrap): C_13_H_16_ONaS, calcd.: 243.0814, found: 243.0818 [M + Na]^+^.

#### 1-(4-Methoxyphenyl)-2-(tetrahydro-1λ^4^-thien-1-ylidene)ethan-1-one **(2f)**

Prepared according to **GP1** from 2-bromo-1-(4-methoxyphenyl)ethanone (344 mg) afforded sulfonium ylide **2f** (235 mg, 0.99 mmol, 66%) as a red oil. FTIR (ATR):  [cm^–1^] = 2933, 1664, 1589, 1543, 1509, 1478, 1393, 1330, 1307, 1270, 1174, 1094, 1020, 1007. ^1^H NMR (300 MHz, CDCl_3_): δ (ppm) = 7.80–7.69 (m, 2H), 6.91–6.78 (m, 2H), 4.27 (s, 1H), 3.82 (s, 3H), 3.62 (dt, *J* = 12.9, 7.3 Hz, 2H), 3.08 (dt, *J* = 11.9, 6.4 Hz, 2H), 2.85–2.67 (m, 2H), 2.07–1.87 (m, 2H). ^13^C{^1^H} NMR: Sulfonium ylide **2f** shows low stability in solution and undergoes decomposition. Therefore, it was not possible to obtain a clean ^13^C NMR spectrum. HRMS (ESI, Orbitrap): C_13_H_17_O_2_S calcd.: 237.0949, found: 237.0945 [M + H]^+^.

#### 2-(Tetrahydro-1λ^4^-thien-1-ylidene)-1-(4-(trifluoromethyl)phenyl)ethan-1-one **(2g)**

Prepared according to **GP1** from 2-bromo-1-(4-trifluoromethyl phenyl)ethanone (400 mg) afforded sulfonium ylide **2g** (218 mg, 0.79 mmol, 53%) as a pale-yellow solid. m.p.: 72–73 °C. FTIR (ATR):  [cm^–1^] = 2936, 1614, 1587, 1519, 1505, 1443, 1393, 1325, 1273, 1206, 1151, 1107, 1014. ^1^H NMR (500 MHz, CDCl_3_): δ (ppm) = 7.89–7.83 (m, 2H), 7.62–7.56 (m, 2H), 4.36 (s, 1H), 3.71–3.55 (m, 2H), 3.20–3.05 (m, 2H), 2.84–2.69 (m, 2H), 2.03 (dqd, *J* = 11.4, 7.2, 2.2 Hz, 2H). ^13^C{^1^H} NMR: Sulfonium ylide **2g** shows low stability in solution and undergoes decomposition. Therefore, it was not possible to obtain a clean ^13^C NMR spectrum. ^19^F NMR (471 MHz, CDCl_3_): δ (ppm) = – 62.5 (m). HRMS (ESI, Orbitrap): C_13_H_14_OF_3_S calcd.: 275.0712, found: 275.0714 [M + H]^+^.

### General Procedure (GP2) for the Synthesis of Cyclobutanones **3a**–**3o**

A flame-dried (2×) 25 mL Schlenk flask was charged with SCP **1a**–**1h** (0.30 mmol, 1.00 equiv), K_3_PO_4_ (0.45 mmol, 1.50 equiv) and the corresponding sulfonium ylide **2a**–**2g** (0.45 mmol, 1.50 equiv). The flask was evacuated and backfilled with argon (2×) before anhydrous Toluene (12 mL) was added. The reaction mixture was degassed by freezing pump thaw (3×), placed into a preheated oil bath at 100 °C and stirred for the indicated time. Afterward the reaction mixture was cooled to r.t., solids were removed by filtration through filter paper and the filtrate was concentrated under reduced pressure. Purification by flash column chromatography (SiO_2_, *n*-pentane/EtOAc) yielded the respective cyclobutanones **3a**–**3o**.

#### 2-Benzoylcyclobutan-1-one **(3a)**

Prepared according to **GP2** from SCP **1a** (64 mg) and sulfonium ylide **2a** (93 mg) for 30 min. Purification by flash column chromatography (SiO_2_, *n*-pentane/EtOAc 19:1) afforded the cyclobutanone **3a** (24 mg, 0.14 mmol, 46%) as a yellow oil. *R*_f_: 0.44 (*n*-pentane/EtOAc 9:1). FTIR (ATR):  [cm^–1^] = 2965, 2943, 2699, 1691, 1673, 1596, 1580, 1449, 1410, 1377, 1320, 1308, 1285, 1231, 1190, 1155, 1071. ^1^H NMR (500 MHz, CDCl_3_): δ (ppm) = 8.10–8.01 (m, 2H), 7.65–7.40 (m, 3H), 5.25–5.14 (m, 1H), 3.25–3.16 (m, 2H), 2.88 (dddd, *J* = 11.7, 9.4, 8.4, 6.4 Hz, 1H), 2.26–2.13 (m, 1H). ^13^C{^1^H} NMR (126 MHz, CDCl_3_): δ (ppm) = 200.9, 190.4, 136.3, 134.0, 129.8, 129.1, 71.0, 46.9, 12.0. HRMS (GC-APCI, QTOF): C_11_H_11_O_2_, calcd.: 175.0754, found: 175.0751 [M + H]^+^.

#### 2-(4-Fluorobenzoyl)cyclobutan-1-one **(3b)**

Prepared according to **GP2** from SCP **1a** (64 mg) and sulfonium ylide **2b** (101 mg) for 30 min. Purification by flash column chromatography (SiO_2_, *n*-pentane/EtOAc 19:1) afforded the cyclobutanone **3b** (26 mg, 0.14 mmol, 45%) as a yellow oil. *R*_f_: 0.33 (*n*-pentane/EtOAc 9:1). FTIR (ATR):  [cm^–1^] = 2970, 2936, 1777, 1668, 1592, 1505, 1410, 1308, 1288, 1212, 1195, 1160, 1144, 1074, 1055, 1033. ^1^H NMR (500 MHz, CDCl_3_): δ (ppm) = 8.16–8.02 (m, 2H), 7.22–7.10 (m, 2H), 5.20–5.07 (m, 1H), 3.27–3.11 (m, 2H), 2.93–2.81 (m, 1H), 2.27–2.12 (m, 1H). ^13^C{^1^H} NMR (126 MHz, CDCl_3_): δ (ppm) = 200.8, 188.7, 166.5 (d, *J* = 255.7 Hz), 132.8 (d, *J* = 2.9 Hz), 132.5 (d, *J* = 9.6 Hz), 116.2 (d, *J* = 22.2 Hz), 71.0, 46.8, 11.9. ^19^F NMR (471 MHz, CDCl_3_): δ (ppm) = – 104.28 (m). HRMS (GC-APCI, QTOF): C_11_H_10_FO_2_, calcd.: 193.0659, found: 193.0656 [M + H]^+^.

#### 2-(4-Chlorobenzoyl)cyclobutan-1-one **(3c)**

Prepared according to **GP2** from SCP **1a** (64 mg) and sulfonium ylide **2c** (108 mg) for 30 min. Purification by flash column chromatography (SiO_2_, *n*-pentane/EtOAc 19:1) afforded the cyclobutanone **3c** (23 mg, 0.11 mmol, 37%) as a yellow oil. *R*_f_: 0.26 (*n*-pentane:EtOAc 9:1). FTIR (ATR):  [cm^–1^] = 2970, 2918, 1777, 1708, 1663, 1587, 1572, 1487, 1401, 1299, 1239, 1215, 1197, 1178, 1145, 1088, 1040, 1008. ^1^H NMR (500 MHz, CDCl_3_): δ (ppm) = 8.07–7.91 (m, 2H), 7.53–7.39 (m, 2H), 5.23–5.05 (m, 1H), 3.35–3.15 (m, 2H), 2.89–2.64 (m, 1H), 2.31–2.07 (m, 1H). ^13^C{^1^H} NMR (126 MHz, CDCl_3_): δ (ppm) = 200.1, 188.6, 140.0, 134.1, 130.6, 128.9, 70.5, 46.3, 11.4. HRMS (GC-APCI, QTOF): C_11_H_10_ClO_2_, calcd.: 209.0364, found: 209.0360 [M + H]^+^.

#### 2-(4-Bromobenzoyl)cyclobutan-1-one **(3d)**

Prepared according to **GP2** from SCP **1a** (64 mg) and sulfonium ylide **2d** (127 mg) for 30 min. Purification by flash column chromatography (SiO_2_, *n*-pentane/EtOAc 19:1) afforded the cyclobutanone **3d** (28 mg, 0.11 mmol, 37%) as a yellow oil. *R*_f_: 0.27 (*n*-pentane:EtOAc 9:1). FTIR (ATR):  [cm^–1^] = 2802, 1695, 1581, 1486, 1450, 1433, 1401, 1372, 1301, 1279, 1225, 1192, 1115, 1071, 1012. ^1^H NMR (500 MHz, CDCl_3_): δ (ppm) = 7.98–7.87 (m, 2H), 7.70–7.56 (m, 2H), 5.20–5.06 (m, 1H), 3.28–3.14 (m, 2H), 2.95–2.80 (m, 1H), 2.20 (dtd, *J* = 11.7, 9.2, 7.9 Hz, 1H). ^13^C{^1^H} NMR (126 MHz, CDCl_3_): δ (ppm) = 200.6, 189.4, 135.1, 132.4, 131.2, 129.4, 71.0, 46.9, 11.9. HRMS (GC-APCI, QTOF): C_11_H_10_BrO_2_, calcd.: 252.9859, found: 252.9854 [M + H]^+^.

#### 2-(4-Methylbenzoyl)cyclobutan-1-one **(3e)**

Prepared according to **GP2** from SCP **1a** (64 mg) and sulfonium ylide **2e** (99 mg) for 30 min. Purification by flash column chromatography (SiO_2_, *n*-pentane/EtOAc 19:1) afforded the cyclobutanone **3e** (19 mg, 0.10 mmol, 34%) as a colorless solid. m.p.: 110–111 °C. *R*_f_: 0.45 (*n*-pentane:EtOAc 9:1). FTIR (ATR):  [cm^–1^] = 2965, 1768, 1667, 1604, 1315, 1297, 1223, 1204, 1180, 1142, 1055, 1009. ^1^H NMR (300 MHz, CDCl_3_): δ (ppm) = 8.02–7.88 (m, 2H), 7.32–7.25 (m, 2H), 5.22–5.10 (m, 1H), 3.25–3.15 (m, 2H), 2.94–2.79 (m, 1H), 2.42 (s, 3H), 2.25–2.09 (m, 1H). ^13^C{^1^H} NMR (101 MHz, CDCl_3_): δ (ppm) = 201.2, 190.0, 145.0, 134.0, 129.9, 129.8, 71.0, 46.9, 22.2, 12.1. HRMS (GC-APCI, QTOF): C_12_H_12_O_2_, calcd.: 188.0832, found: 188.0830 [M]^+^.

#### 2-(4-Methoxybenzoyl)cyclobutan-1-one **(3f)**

Prepared according to **GP2** from SCP **1a** (64 mg) and sulfonium ylide **2f** (106 mg) for 30 min. Purification by flash column chromatography (SiO_2_, *n*-pentane/EtOAc 19:1) afforded the cyclobutanone **3f** (20 mg, 0.10 mmol, 32%) as a colorless solid. m.p.: 118–119 °C. *R*_f_: 0.23 (*n*-pentane:EtOAc 9:1). FTIR (ATR):  [cm^–1^] = 2836, 1706, 1670, 1596, 1573, 1510, 1461, 1415, 1373, 1312, 1289, 1252, 1233, 1195, 1177, 1115, 1077, 1021. ^1^H NMR (400 MHz, CDCl_3_): δ (ppm) = 8.10–7.98 (m, 2H), 6.99–6.92 (m, 2H), 5.21–5.07 (m, 1H), 3.87 (s, 3H), 3.23–3.10 (m, 2H), 2.92–2.79 (m, 1H), 2.25–2.09 (m, 1H). ^13^C{^1^H} NMR (101 MHz, CDCl_3_): δ (ppm) = 201.5, 188.9, 164.4, 132.2, 129.5, 114.4, 70.7, 56.0, 46.8, 12.1. HRMS (GC-APCI, QTOF): C_12_H_13_O_3_, calcd.: 205.0859, found: 205.0853 [M + H]^+^.

#### 2-(4-(Trifluoromethyl)benzoyl)cyclobutan-1-one **(3g)**

Prepared according to **GP2** from SCP **1a** (64 mg) and sulfonium ylide **2g** (123 mg) for 30 min. Purification by flash column chromatography (SiO_2_, *n*-pentane/EtOAc 19:1) afforded the cyclobutanone **3g** (28 mg, 0.12 mmol, 38%) as a yellow solid. m.p.: 88–89 °C. *R*_f_: 0.25 (*n*-pentane:EtOAc 9:1). FTIR (ATR):  [cm^–1^] = 2925, 1784, 1685, 1581, 1513, 1457, 1409, 1319, 1283, 1223, 1167, 1130, 1110, 1063, 1015. ^1^H NMR (700 MHz, CDCl_3_): δ (ppm) = 8.22–8.11 (m, 2H), 7.82–7.70 m, 2H), 5.28–5.12 (m, 1H), 3.31–3.16 (m, 2H), 2.98–2.85 (m, 1H), 2.29–2.16 (m, 1H). ^13^C{^1^H} NMR (176 MHz, CDCl_3_): δ (ppm) = 199.9, 189.2, 138.7 (d, *J* = 1.3 Hz), 134.9 (d, *J* = 32.6 Hz), 129.8, 125.9 (d, *J* = 3.8 Hz), 123.7 (d, *J* = 272.8 Hz), 71.1, 46.7, 11.6. ^19^F NMR (471 MHz, CDCl_3_): δ (ppm) = – 63.21 (m). HRMS (GC-APCI, QTOF): C_12_H_10_F_3_O_2_, calcd.: 243.0633, found: 243.0632 [M + H]^+^.

#### (*R*)-2-Benzoyl-3,3-dimethylcyclobutan-1-one **(3h)**

Prepared according to **GP2** from SCP **1b** (68 mg) and sulfonium ylide **2a** (93 mg) for 30 min. Purification by flash column chromatography (SiO_2_, *n*-pentane/EtOAc 19:1) afforded the cyclobutanone **3h** (19 mg, 0.09 mmol, 31%) as a yellow oil. *R*_f_: 0.39 (*n*-pentane:EtOAc 19:1). FTIR (ATR):  [cm^–1^] = 2954, 1690, 1597, 1580, 1448, 1362, 1324, 1230, 1177, 1116, 1009. ^1^H NMR (400 MHz, CDCl_3_): δ (ppm) = 7.88–7.84 (m, 2H), 7.61–7.56 (m, 1H), 7.51–7.45 (m, 2H), 4.69 (dd, *J* = 2.1, 0.7 Hz, 1H), 2.98–2.95 (m, 2H), 1.60 (s, 3H), 1.28 (s, 3H). ^13^C{^1^H} NMR (101 MHz, CDCl_3_): δ (ppm) = 201.7, 194.1, 136.8, 133.4, 128.6, 128.2, 74.2, 59.0, 32.3, 29.5, 23.8. HRMS (GC-APCI, QTOF): C_13_H_15_O_2_, calcd.: 203.1067, found: 203.1065 [M + H]^+^.

#### (R)-2-(4-Fluorobenzoyl)-3,3-dimethylcyclobutan-1-one **(3i)**

Prepared according to **GP2** from SCP **1b** (68 mg) and sulfonium ylide **2b** (101 mg) for 30 min. Purification by flash column chromatography (SiO_2_, *n*-pentane/EtOAc 19:1) afforded the cyclobutanone **3i** (17 mg, 0.08 mmol, 26%) as a yellow oil. *R*_f_: 0.31 (*n*-pentane:EtOAc 19:1). FTIR (ATR):  [cm^–1^] = 2957, 1690, 1575, 1580, 1447, 1362, 1324, 1232, 1177, 1116, 1009. ^1^H NMR (500 MHz, CDCl_3_): δ (ppm) = 7.93–7.87 (m, 2H), 7.18–7.13 (m, 2H), 4.63 (d, *J* = 1.10 Hz, 1H), 2.97 (d, 2H), 1.60 (s, 3H), 1.29 (s, 3H). ^13^C{^1^H} NMR (126 MHz, CDCl_3_): δ (ppm) = 201.9, 192.9, 166.5 (d, *J* = 256.0 Hz), 133.7 (d, *J* = 3.0 Hz), 131.5 (d, *J* = 9.7 Hz), 116.4 (d, *J* = 22.0 Hz), 74.6, 59.5, 32.9, 30.1, 24.4. ^19^F NMR (471 MHz, CDCl_3_): δ (ppm) = – 104.16 (m). HRMS (GC-APCI, QTOF): C_13_H_14_FO_2_, calcd.: 221.0975, found: 221.0973 [M + H]^+^.

#### (2*R*,3*S*)-2-Benzoyl-3-phenylcyclobutan-1-one **(3j)**

Prepared according to **GP2** from SCP **1c** (82 mg) and sulfonium ylide **2a** (93 mg) for 30 min. Purification by flash column chromatography (SiO_2_, *n*-pentane/EtOAc 19:1) afforded the cyclobutanone **3j** (19 mg, 0.08 mmol, 25%) as a colorless solid. m.p.: 123–124 °C. *R*_f_: 0.49 (*n*-pentane:EtOAc 9:1). FTIR (ATR):  [cm^–1^] = 1784, 1669, 1596, 1449, 1322, 1297, 1270, 1229, 1139, 1080. ^1^H NMR (500 MHz, CDCl_3_): δ (ppm) = 8.01–7.89 (m, 2H), 7.53–7.46 (m, 1H), 7.43–7.38 (m, 2H), 7.29–7.26 (m, 2H), 7.25 (t, *J* = 1.9 Hz, 1H), 7.21–7.15 (m, 2H), 5.08 (dt, *J* = 7.5, 2.5 Hz, 1H), 4.42 (dt, *J* = 9.9, 7.8 Hz, 1H), 3.47 (ddd, *J* = 17.9, 9.8, 2.3 Hz, 1H), 3.35 (ddd, *J* = 18.0, 8.1, 2.6 Hz, 1H). ^13^C{^1^H} NMR (126 MHz, CDCl_3_): δ (ppm) = 199.2, 190.1, 142.4, 136.2, 134.2, 129.9, 129.3, 129.1, 127.4, 127.1, 78.0, 51.9, 30.3. HRMS (GC-APCI, QTOF): C_17_H_15_O_2_, calcd.: 251.1067, found: 251.1066 [M + H]^+^.

#### (2*R*,3*S*)-2-Benzoyl-3-methylcyclobutan-1-one **(3k)**

Prepared according to **GP2** from SCP **1d** (64 mg) and sulfonium ylide **2a** (93 mg) for 30 min. Purification by flash column chromatography (SiO_2_, *n*-pentane/EtOAc 19:1) afforded the cyclobutanone **3k** (28 mg, 0.15 mmol, 50%) as a yellow oil. *R*_f_: 0.27 (*n*-pentane:EtOAc 19:1). FTIR (ATR):  [cm^–1^] = 2965, 1679, 1597, 1447, 1411, 1371, 1292, 1260, 1221, 1179, 1160, 1002. ^1^H NMR (300 MHz, CDCl_3_): δ (ppm) = 8.07–7.99 (m, 2H), 7.64–7.45 (m, 3H), 4.89–4.57 (m, 1H), 3.42–3.20 (m, 2H), 2.93–2.65 (m, 1H), 1.47–1.33 (m, 3H). ^13^C{^1^H} NMR (126 MHz, CDCl_3_): δ (ppm) = 200.4, 190.7, 136.5, 133.9, 129.7, 129.0, 77.2, 52.9, 21.0, 20.8. HRMS (GC-APCI, QTOF): C_12_H_13_O_2_, calcd.: 189.0910, found: 189.0908 [M + H]^+^.

#### (2*R*,3*S*)-2-(4-Fluorobenzoyl)-3-methylcyclobutan-1-one **(3l)**

Prepared according to **GP2** from SCP **1d** (64 mg) and sulfonium ylide **2b** (101 mg) for 30 min. Purification by flash column chromatography (SiO_2_, *n*-pentane/EtOAc 19:1) afforded the cyclobutanone **3l** (35 mg, 0.17 mmol, 57%) as a yellow oil. *R*_f_: 0.23 (*n*-pentane:EtOAc 19:1). FTIR (ATR):  [cm^–1^] = 2976, 2765, 1777, 1670, 1596, 1506, 1446, 1411, 1371, 1285, 1234, 1209, 1156. ^1^H NMR (700 MHz, CDCl_3_): δ (ppm) = 8.09–8.02 (m, 2H), 7.20–7.13 (m, 2H), 4.76–4.55 (m, 1H), 3.38–3.19 (m, 2H), 2.90–2.68 (m, 1H), 1.47–1.30 (m, 3H). ^13^C{^1^H} NMR (176 MHz, CDCl_3_): δ (ppm) = 199.8, 188.6, 166.0 (d, *J* = 255.9 Hz), 132.5 (d, *J* = 3.0 Hz), 132.0 (d, *J* = 9.5 Hz), 115.8 (d, *J* = 22.0 Hz), 76.7, 52.4, 20.5, 20.4. ^19^F NMR (659 MHz, CDCl_3_): δ (ppm) = – 104.33 (m). HRMS (GC-APCI, QTOF): C_12_H_12_FO_2_, calcd.: 207.0816, found: 207.0814 [M + H]^+^.

#### (1*S*,6*R*,8*R*)-8-Benzoylbicyclo[4.2.0]octan-7-one **(3m)**

Prepared according to **GP2** from SCP **1e** (76 mg) and sulfonium ylide **2a** (93 mg) for 30 min. Purification by flash column chromatography (SiO_2_, *n*-pentane/EtOAc 49:1) afforded cyclobutanone **3m** (37 mg, 0.16 mmol, 54%) as a pale-yellow solid. On a 1 mmol scale: Prepared according to **GP2**. A flame-dried (2×) 100 mL Schlenk flask was charged with SCP **1e** (252 mg), K_3_PO_4_ (318 mg) and corresponding sulfonium ylide **2a** (310 mg). The flask was evacuated and backfilled with argon (2×) before anhydrous Toluene (40 mL) was added. The reaction mixture was degassed by freezing pump thaw (1×), placed into a preheated oil bath at 100 °C and stirred for the 30 min. Afterward the reaction mixture was cooled to r.t., solids were removed by filtration through filter paper and the filtrate was concentrated under reduced pressure. Purification by flash column chromatography (SiO_2_, *n*-pentane/EtOAc 49:1) yielded respective cyclobutanone **3m** (136 mg, 0.60 mmol, 60%) as a pale-yellow solid. m.p.: 62–63 °C. *R*_f_: 0.29 (*n*-pentane:EtOAc 19:1). FTIR (ATR):  [cm^–1^] = 2929, 2854, 1761, 1663, 1596, 1581, 1448, 1340, 1326, 1309, 1285, 1264, 1221, 1192, 1159, 1137, 1034, 1024. ^1^H NMR (300 MHz, CDCl_3_): δ (ppm) = 8.21–7.97 (m, 2H), 7.68–7.39 (m, 3H), 4.69 (dd, *J* = 3.6, 1.7 Hz, 1H), 3.54 (dd, *J* = 10.2, 7.9 Hz, 1H), 3.28–3.00 (m, 1H), 2.29–2.09 (m, 1H), 2.03–1.83 (m, 1H), 1.68–1.51 (m, 2H), 1.51–1.46 (m, 1H), 1.41–1.11 (m, 3H). ^13^C{^1^H} NMR (126 MHz, CDCl_3_): δ (ppm) = 200.5, 190.8, 136.2, 133.3, 128.9, 128.6, 76.7, 56.9, 27.6, 24.9, 22.3, 22.2, 21.1. HRMS (GC-APCI, QTOF): C_15_H_17_O_2_, calcd.: 229.1223, found: 229.1225 [M + H]^+^.

#### (1*S*,6*R*,8*R*)-8-(4-Fluorobenzoyl)bicyclo[4.2.0]octan-7-one **(3n)**

Prepared according to **GP2** from SCP **1e** (76 mg) and sulfonium ylide **2b** (101 mg) for 30 min. Purification by flash column chromatography (SiO_2_, *n*-pentane/EtOAc 49:1) afforded the cyclobutanone **3n** (30 mg, 0.12 mmol, 41%) as a colorless solid. m.p.: 50–51 °C. *R*_f_: 0.30 (*n*-pentane:EtOAc 19:1). FTIR (ATR):  [cm^–1^] = 2983, 2929, 2855, 1763, 1675, 1595, 1504, 1447, 1409, 1351, 1318, 1279, 1262, 1215, 1190, 1154, 1101, 1059, 1033, 1010. ^1^H NMR (500 MHz, CDCl_3_): δ (ppm) = 8.16–8.04 (m, 2H), 7.19–7.13 (m, 2H), 4.64 (dd, *J* = 3.7, 1.8 Hz, 1H), 3.51 (ddt, *J* = 10.7, 7.4, 2.2 Hz, 1H), 3.15 (dddd, *J* = 11.0, 8.6, 7.5, 3.6 Hz, 1H), 2.23–2.11 (m, 1H), 2.00–1.88 (m, 1H), 1.67–1.51 (m, 2H), 1.51–1.47 (m, 1H), 1.36–1.24 (m, 3H). ^13^C{^1^H} NMR (126 MHz, CDCl_3_): δ (ppm) = 200.8, 189.4, 166.1 (d, *J* = 255.6 Hz), 132.8 (d, *J* = 2.8 Hz), 131.9 (d, *J* = 9.3 Hz), 116.0 (d, *J* = 22.2 Hz), 76.8, 57.1, 27.8, 25.0, 22.5, 22.5, 21.4. ^19^F NMR (471 MHz, CDCl_3_): δ (ppm) = – 104.46 (m). HRMS (GC-APCI, QTOF): C_15_H_16_FO_2_, calcd.: 247.1129, found: 247.1126 [M + H]^+^.

#### (1*S*,6*R*,8*R*)-8-(4-Bromobenzoyl)bicyclo[4.2.0]octan-7-one **(3o)**

Prepared according to **GP2** from SCP **1e** (76 mg) and sulfonium ylide **2d** (128 mg) for 30 min. Purification by flash column chromatography (SiO_2_, *n*-pentane/EtOAc 49:1) afforded cyclobutanone **3o** (28 mg, 0.09 mmol, 30%) as a colorless solid. m.p.: 87–88 °C. *R*_f_: 0.39 (*n*-pentane:EtOAc 19:1). FTIR (ATR):  [cm^–1^] = 2937, 2849, 1771, 1666, 1577, 1395, 1322, 1281, 1217, 1192, 1066, 1036, 1002. ^1^H NMR (700 MHz, CDCl_3_): δ (ppm) = 7.97–7.90 (m, 2H), 7.67–7.56 (m, 2H), 4.63 (dd, *J* = 3.7, 1.8 Hz, 1H), 3.58–3.45 (m, 1H), 3.20–3.09 (m, 1H), 2.24– 2.12 (m, 1H), 2.01–1.91 (m, 1H), 1.64–1.59 (m, 1H), 1.55 (dddd, *J* = 13.1, 10.6, 9.2, 6.3 Hz, 2H), 1.36–1.24 (m, 3H). ^13^C{^1^H} NMR (176 MHz, CDCl_3_): δ (ppm) = 200.5, 190.0, 135.1, 132.2, 130.7, 129.0, 76.9, 57.2, 27.8, 25.0, 22.5, 22.5, 21.4. HRMS (GC-APCI, QTOF): C_15_H_16_BrO_2_, calcd.: 307.0328, found: 307.0328 [M + H]^+^.

#### (1*S*,6*R*,8*S*)-8-Benzoyl-8-ethylbicyclo[4.2.0]octan-7-one **(4)**

A flame-dried (2×) microwave vial was charged with cyclobutanone **3m** (23 mg, 0.10 mmol, 1.00 equiv) and K_2_CO_3_ (22 mg, 0.16 mmol, 1.60 equiv). The vial was evacuated and backfilled with argon (2×) before anhydrous acetone (1.4 mL) was added. The reaction mixture was placed into preheated oil bath at 50 °C, then EtI (78 mg, 0.50 mmol, 0.04 mL, 5.00 equiv) was added dropwise to the reaction mixture and stirred for 24 h. Upon completion, the reaction mixture was cooled to r.t., the solids were removed by filtration through filter paper and the mixture was concentrated in *vacuo*. Purification by flash column chromatography (SiO_2_, *n*-pentane/EtOAc 49:1) yielded ethyl-substituted cyclobutanone **4** (8 mg, 0.03 mmol, 31%) as a colorless oil. *R*_f_: 0.38 (*n*-pentane:EtOAc 19:1). FTIR (ATR):  [cm^–1^] = 2932, 2857, 1777, 1667, 1595, 1449, 1412, 1238. ^1^H NMR (300 MHz, CDCl_3_): δ (ppm) = 8.23–7.73 (m, 2H), 7.70–7.35 (m, 3H), 3.58 (t, *J* = 8.7 Hz, 1H), 2.72 (td, *J* = 9.9, 7.2 Hz, 1H), 2.45–2.24 (m, 1H), 2.19–2.08 (m, 2H), 2.07–1.93 (m, 1H), 1.51–1.38 (m, 3H), 1.28–0.98 (m, 3H), 0.91 (t, *J* = 7.5 Hz, 3H). ^13^C{^1^H} NMR (126 MHz, CDCl_3_): δ (ppm) = 207.0, 196.3, 135.6, 132.9, 129.3, 128.3, 80.6, 52.1, 34.9, 31.2, 27.3, 22.4, 21.9, 20.4, 10.3. HRMS (GC-APCI, QTOF): C_17_H_21_O_2_, calcd.: 257.1547, found: 257.1543 [M + H]^+^.

#### *N*-Methyl-2-(2-oxo-2-phenylethyl)cyclohexane-1-carboxamide **(5)**

A microwave vial was charged with cyclobutanone **3m** (46 mg, 0.20 mmol, 1.00 equiv). Methylamine (40% in H_2_O, 0.80 mmol, 0.06 mL, 4.00 equiv) was added and the reaction mixture was stirred for 4 h at r.t. The mixture was extracted twice with CH_2_Cl_2_. The combined organic phases were dried over MgSO_4_, filtered and concentrated under vacuum. Purification by flash column chromatography (SiO_2_, *n*-pentane/EtOAc 1:1) yielded cyclohexane **5**. (26 mg, 0.10 mmol, 50%) as a pale-yellow solid. m.p.: 108–109 °C. *R*_f_: 0.30 (*n*-pentane:EtOAc 1:1). FTIR (ATR):  [cm^–1^] = 3292, 2939, 2859, 1687, 1632, 1597, 1559, 1448, 1326, 1217, 1008. ^1^H NMR (700 MHz, CDCl_3_): δ (ppm) = 7.98–7.93 (m, 2H), 7.58–7.41 (m, 3H), 5.61 (s, 1H), 3.04 (dd, *J* = 6.8, 1.4 Hz, 2H), 2.74 (d, *J* = 4.8 Hz, 3H), 2.55 (dtd, *J* = 11.1, 7.0, 4.3 Hz, 1H), 2.46 (dt, *J* = 8.4, 4.4 Hz, 1H), 1.87–1.57 (m, 5H), 1.51–1.34 (m, 3H). ^13^C{^1^H} NMR (176 MHz, CDCl_3_): δ (ppm) = 200.5, 175.3, 137.4, 133.2, 128.7, 128.3, 45.7, 39.5, 34.0, 29.5, 26.5, 26.2, 23.8, 23.0. HRMS (ESI, Orbitrap): C_16_H_22_NO_2_, calcd.: 260.1645, found: 260.1641 [M + H]^+^.

#### (1*S*,6*R*,8*S*)-8-Benzoyl-8-cinnamylbicyclo[4.2.0]octan-7-one **(7)**

In a flame-dried (2×) 25 mL Schlenk flask, cyclobutanone **3m** (68 mg, 0.30 mmol, 1.00 equiv), K_2_CO_3_ (66 mg, 0.48 mmol, 1.60 equiv) and cinnamyl sulfonium salt **6**^14^ (171 mg, 0.60 mmol, 2.00 equiv) were added. The flask was evacuated and backfilled with argon (2×) before anhydrous acetone (14 mL) was added. The reaction mixture was placed into preheated oil bath at 45 °C and the reaction mixture was stirred until full consumption of the starting material was indicated by TLC analysis. Upon completion, the reaction mixture was cooled to r.t., the solids were removed by filtration through filter paper and the mixture was concentrated under vacuum. Purification by flash column chromatography (SiO_2_, *n*-pentane/EtOAc 49:1) yielded cyclobutanone **7** (70 mg, 0.20 mmol, 68%) as a colorless solid. m.p.: 88–87 °C. *R*_f_: 0.32 (*n*-pentane:EtOAc 19:1). FTIR (ATR):  [cm^–1^] = 2927, 2852, 1767, 1660, 1596, 1579, 1493, 1445, 1299, 1259, 1212, 1180, 1156, 1118, 1075, 1027. ^1^H NMR (300 MHz, CDCl_3_): δ (ppm) = 8.04–7.99 (m, 2H), 7.60–7.43 (m, 3H), 7.39–7.26 (m, 3H), 7.26–7.13 (m, 2H), 6.29 (dt, *J* = 15.7, 1.2 Hz, 1H), 6.08 (ddd, *J* = 15.7, 7.9, 6.9 Hz, 1H), 3.64–3.44 (m, 1H), 3.17–2.92 (m, 2H), 2.79 (td, *J* = 9.8, 7.3 Hz, 1H), 2.21–1.99 (m, 2H), 1.50 (td, *J* = 12.7, 6.3 Hz, 3H), 1.24–0.96 (m, 3H). ^13^C{^1^H} NMR (126 MHz, CDCl_3_): δ (ppm) = 206.5, 196.7, 136.8, 136.1, 134.1, 133.1, 129.8, 128.6, 128.5, 127.7, 126.5, 123.7, 80.2, 52.4, 41.2, 34.3, 27.5, 22.6, 22.1, 20.6. HRMS (ESI, Orbitrap): C_24_H_24_O_2_Na, calcd.: 367.1669, found: 367.1667 [M + Na]^+^.

## Data Availability

The data underlying this study are available in the published article and its Supporting Information.

## Supporting Information Available

Detailed experimental procedures and analytical data for all new compounds (PDF)
