# Supplementary material for: Formal Carbene Insertion into Cyclopropanones: Access to 2-Aroyl Cyclobutanones via Sulfonium Ylides
Source: J Org Chem. 2025 Apr 29;90(18):6162–8. doi: 10.1021/acs.joc.5c00167 (PMC12070404; doi:10.1021/acs.joc.5c00167)
Supplement: Supplementary file 1 — jo5c00167_si_001.pdf [file jo5c00167_si_001.pdf]

**Supporting Information**

# Formal Carbene Insertion into Cyclopropanones: Access to 2-Aroyl Cyclobutanones via Sulfonium Ylides

**Ishika Agrawal, Marvin Lange, Arthur Semmelmaier, Heinrich F. von Köller and Daniel B. Werz***

Albert-Ludwigs-Universität Freiburg

Institute of Organic Chemistry

Alberstraße 21, 79104 Freiburg, Germany

(*corresponding author: daniel.werz@chemie.uni-freiburg.de)

## Table of Contents

**General Experimental ........................................ S-2.**

**NMR Spectra of Starting Materials ........................................ S-3.**

**NMR Spectra of Cyclobutanones ........................................ S-13.**

**Follow-up Reactions: NMR Spectra ........................................ S-33.**

**Decomposition of Cyclobutanone ........................................ S-36.**

**Stereospecificity Experiments ........................................ S-38.**

**Optimization of Reaction Conditions ........................................ S-41.**

**Computational Studies ........................................ S-44.**

**Crystal Structure Determinations ........................................ S-48.**

**References ........................................ S-50.**

## General Experimental

Air- and moisture-sensitive reactions were conducted in oven-dried or flame-dried glassware, septum-capped under atmospheric pressure of argon. Solvents used for column chromatography were distilled prior to use. Anhydrous solvents were obtained either from a solvent purification system ($CH_2Cl_2$, $Et_2O$, THF, MeCN, toluene) and stored in flame-dried flasks over activated (4 Å) molecular sieves under argon or were purchased (Thermo Scientific) anhydrous over molecular sieves (MeOH, EtOH, acetone, DMF, $CHCl_3$, $CH_2Cl_2$, EtOAc, pyridine, 1,4-dioxane). Commercially available compounds were used without further purification unless specified otherwise.

For all purifications by flash column chromatography, Silica 60 (40 – 63 μm pore size) from Macherey-Nagel was used. Thin-layer chromatography was carried out on Silica gel 60 F254 aluminum sheets from Merck. For the detection of spots, UV light at 254 nm was used.

NMR spectra for proton ($^1H$), carbon ($^{13}C$), and fluorine ($^{19}F$) were recorded on Bruker instruments at 500 MHz DRX 500 or 400 MHz (Advance II 400) using the residual signals from $CDCl_3$, δ = 7.26 ppm and δ = 77.16 ppm as internal reference for $^1H$ and $^{13}C$ chemical shifts, respectively. Additionally, tetramethylsilane (TMS; $\delta$ = 0.00 ppm; 0.03%) was added to NMR samples. The following abbreviations denote multiplicities in $^1H$, $^{13}C$, and $^{19}F$ NMR chemical shifts: s = singlet, d = doublet, t = triplet, q = quartet, m = multiplet. The chemical shifts δ reported in parts per million (ppm).

High-resolution mass spectroscopy (ESI-HRMS and APCI-HRMS) were carried out on an Thermo Scientific Exactive Orbitrap instrument. ATR-FTIR spectroscopy was carried out on a Spectrum Two FT-IR Spectrometer from Perkin Elmer, with samples measured neat on a diamond ATR crystal. Transmission-bands are reported in $cm^{-1}$.

Melting points of solid products were determined using the open capillary method on a Schorpp MPM-HV2 apparatus. Specific reaction conditions are given in the following procedures.

Enantiomeric excess was determined using an Agilent 1100 series system with a G1311A quarternary pump. A Lux Cellulose-2 column (3 μm; 150 x 4.6 mm) was used with *n*-heptane/*i*-PrOH eluent mixtures. Detection was conducted at 240 nm using a variable wavelength detector (VWD).

## NMR Spectra of Starting Materials 2a-g: Sulfonium Ylides

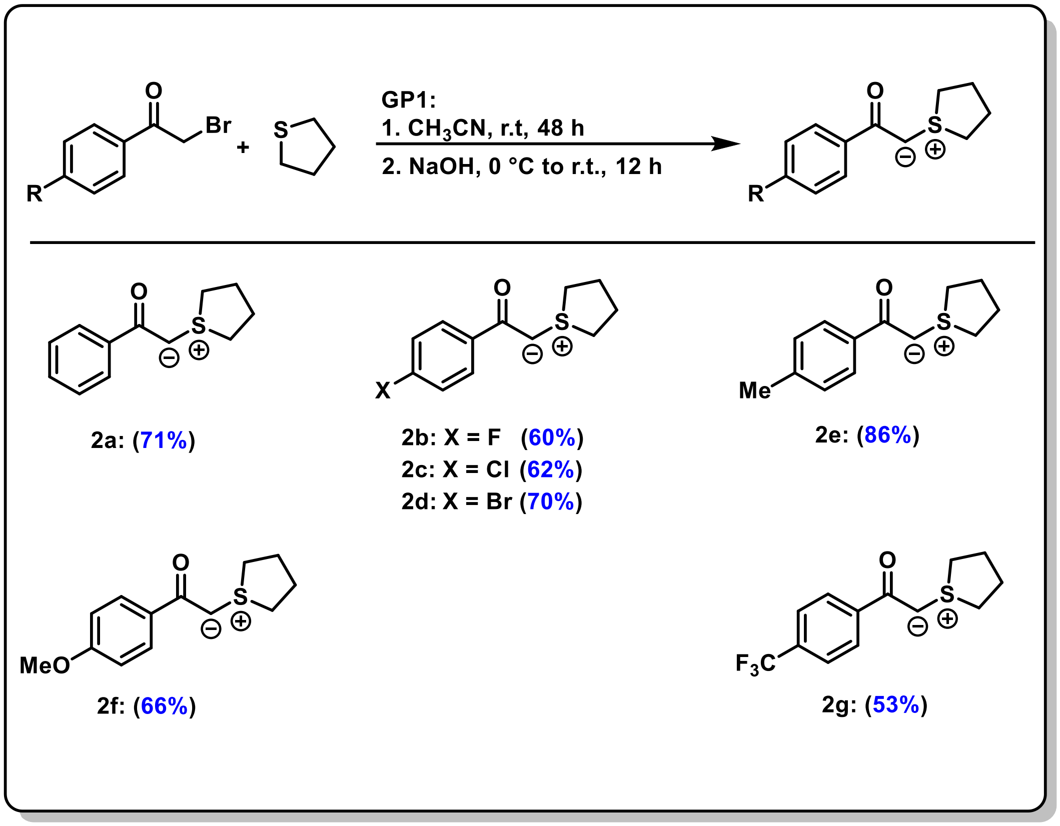

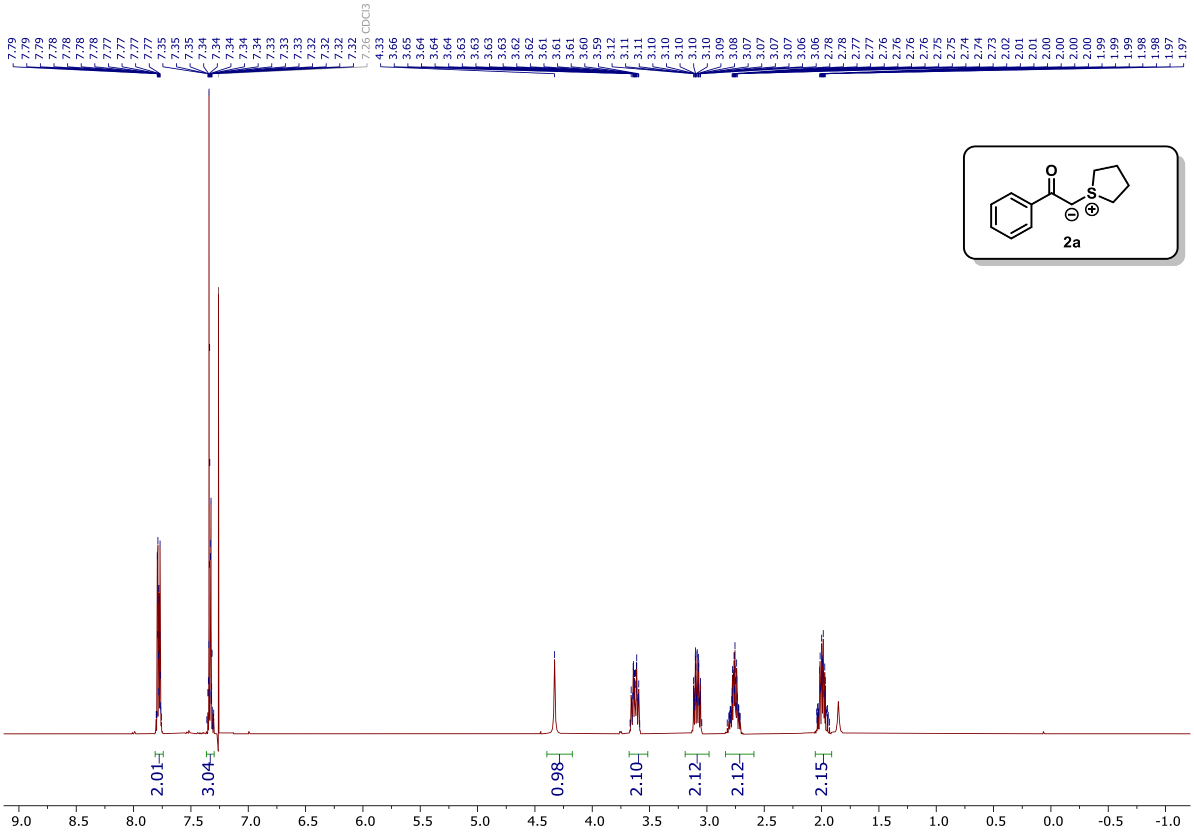

**¹H-NMR** (400 MHz, $CDCl_3$)

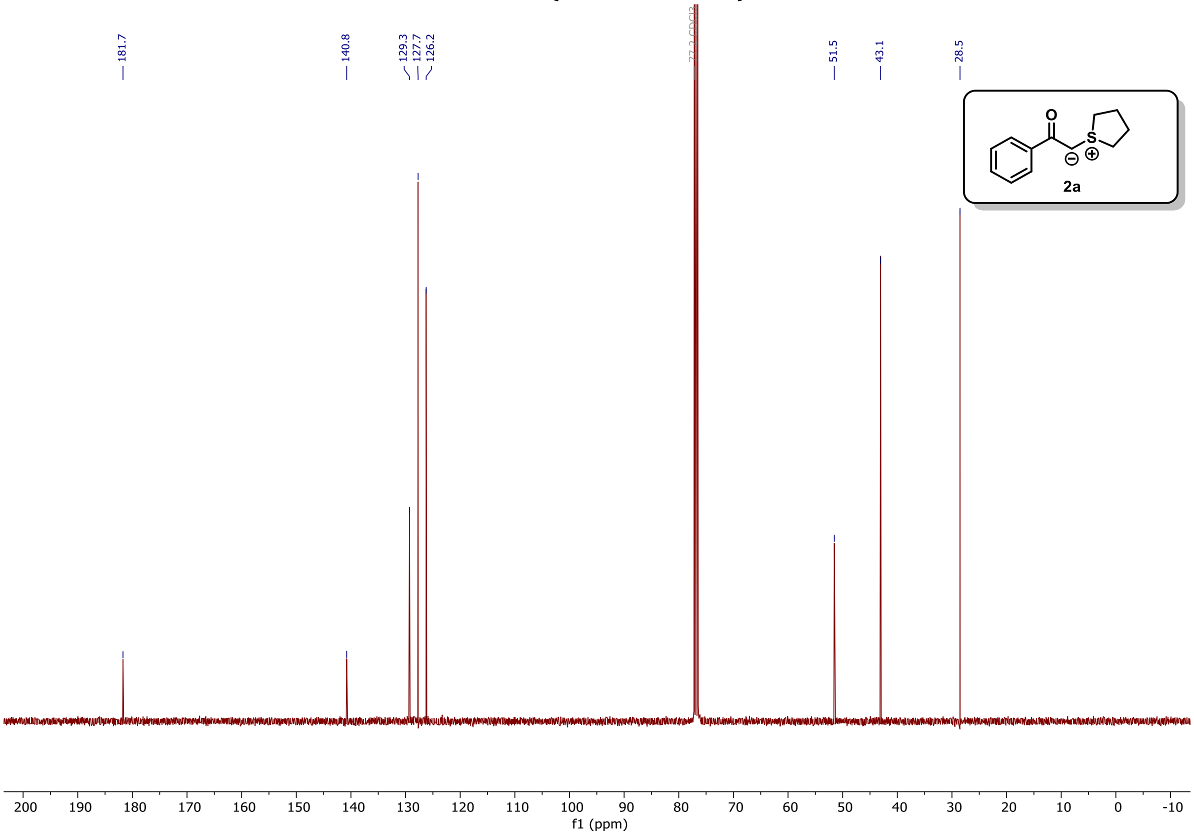

**¹³C{¹H}-NMR** (101 MHz, $CDCl_3$)

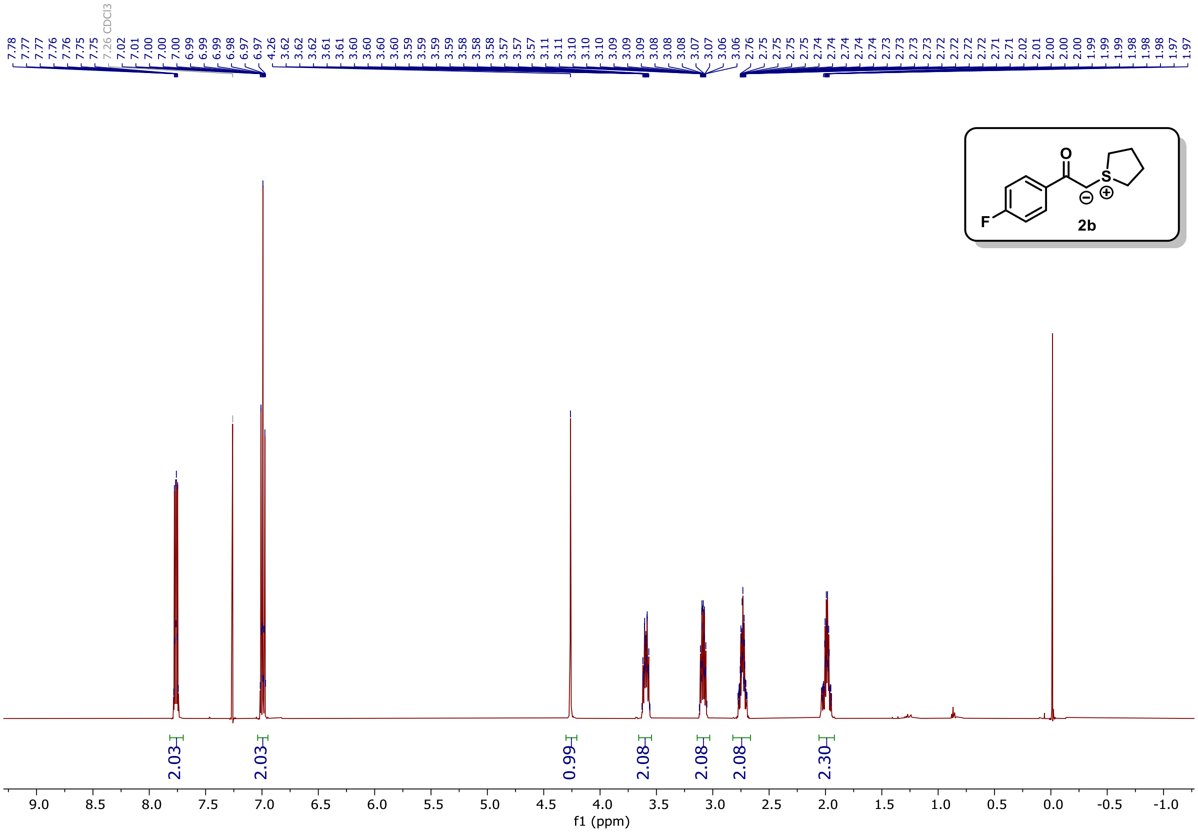


**¹H-NMR** (500 MHz, $CDCl_3$)

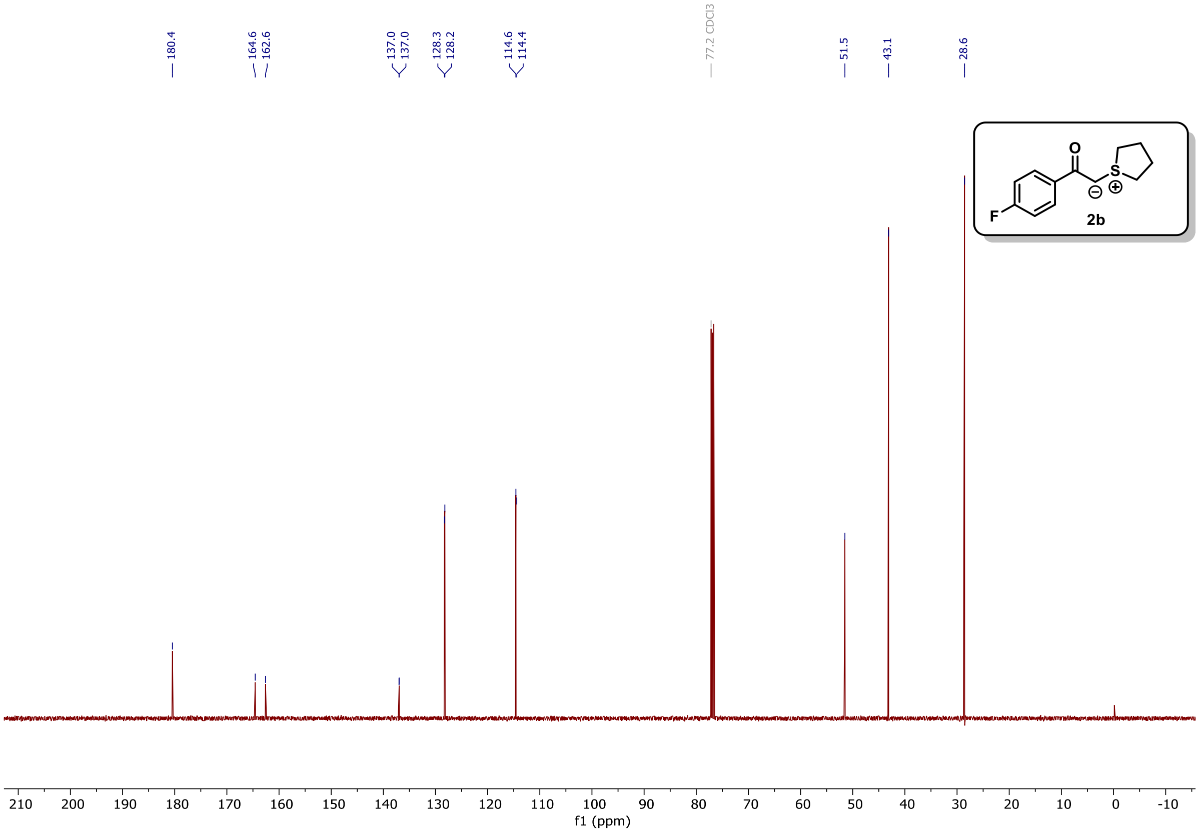


**$^{13}C\{^1H\}$-NMR** (126 MHz, $CDCl_3$)

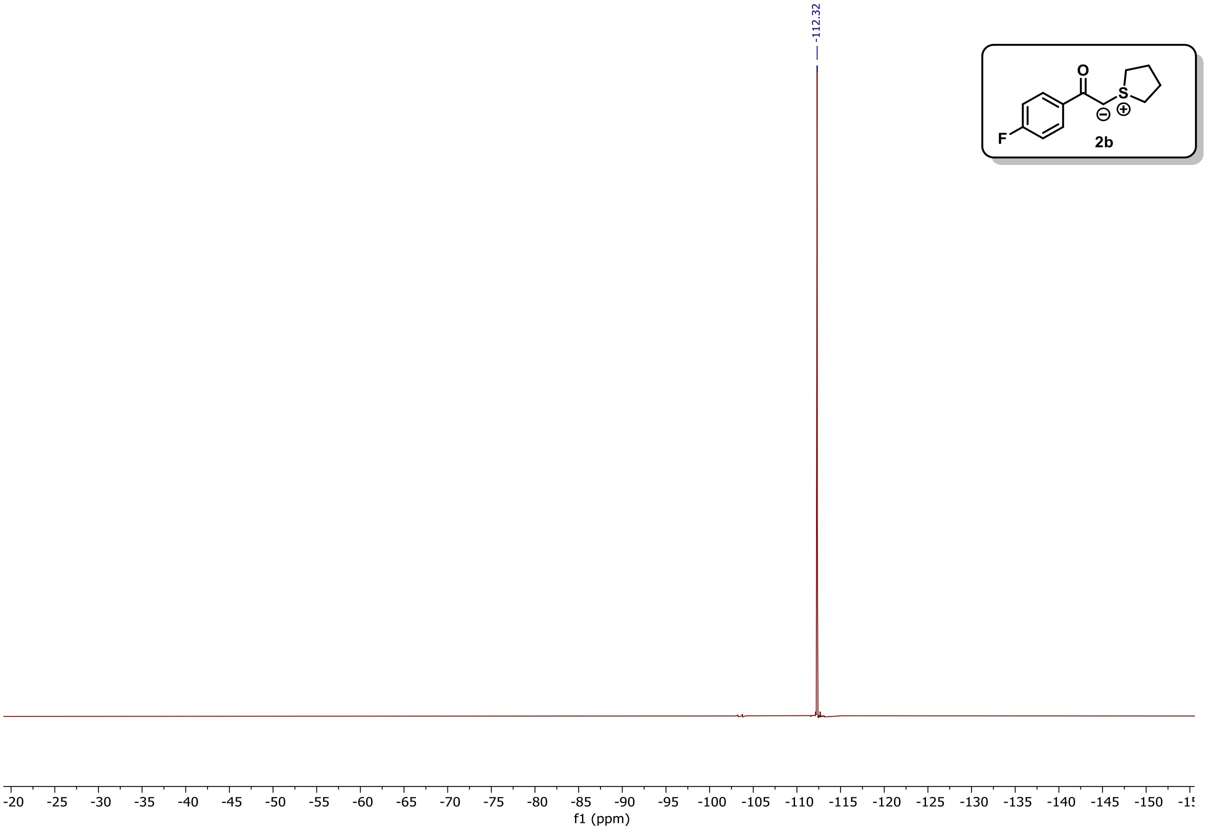


**$^{19}$F-NMR** (471 MHz, $CDCl_3$)

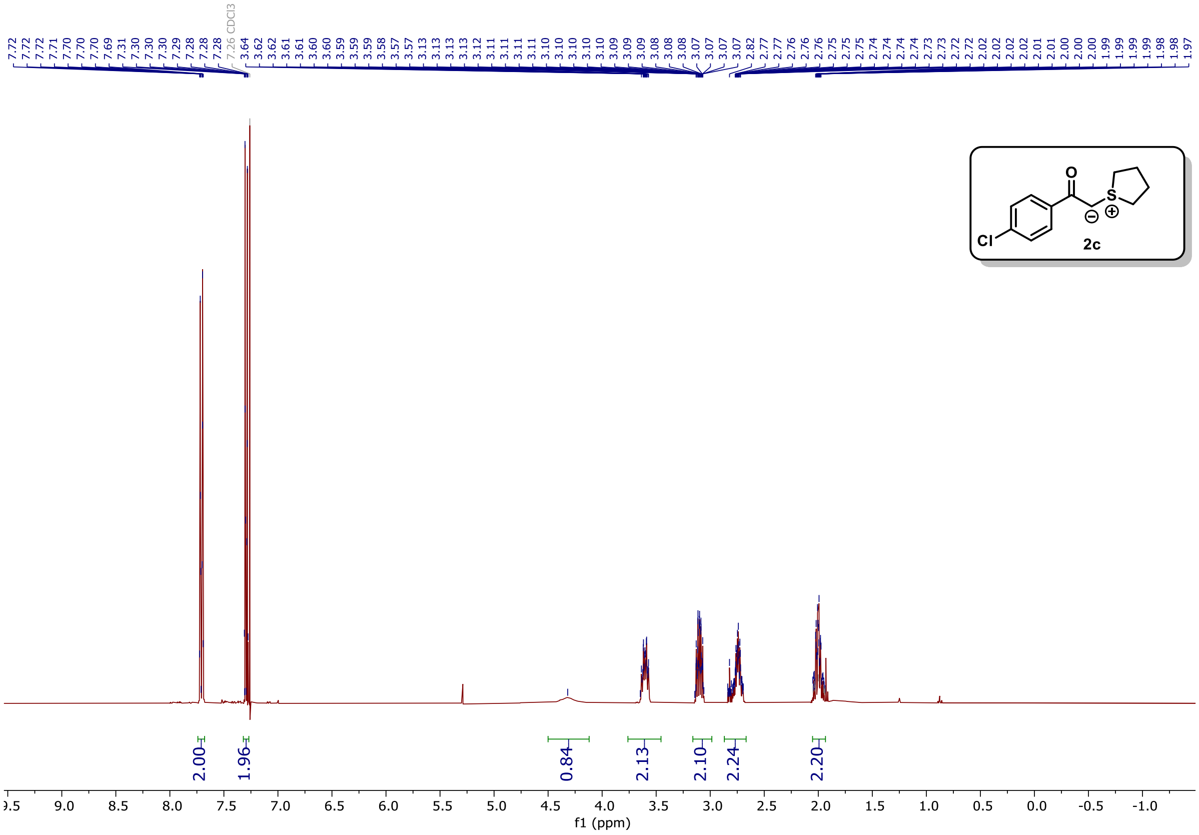


**¹H-NMR** (400 MHz, $CDCl_3$)

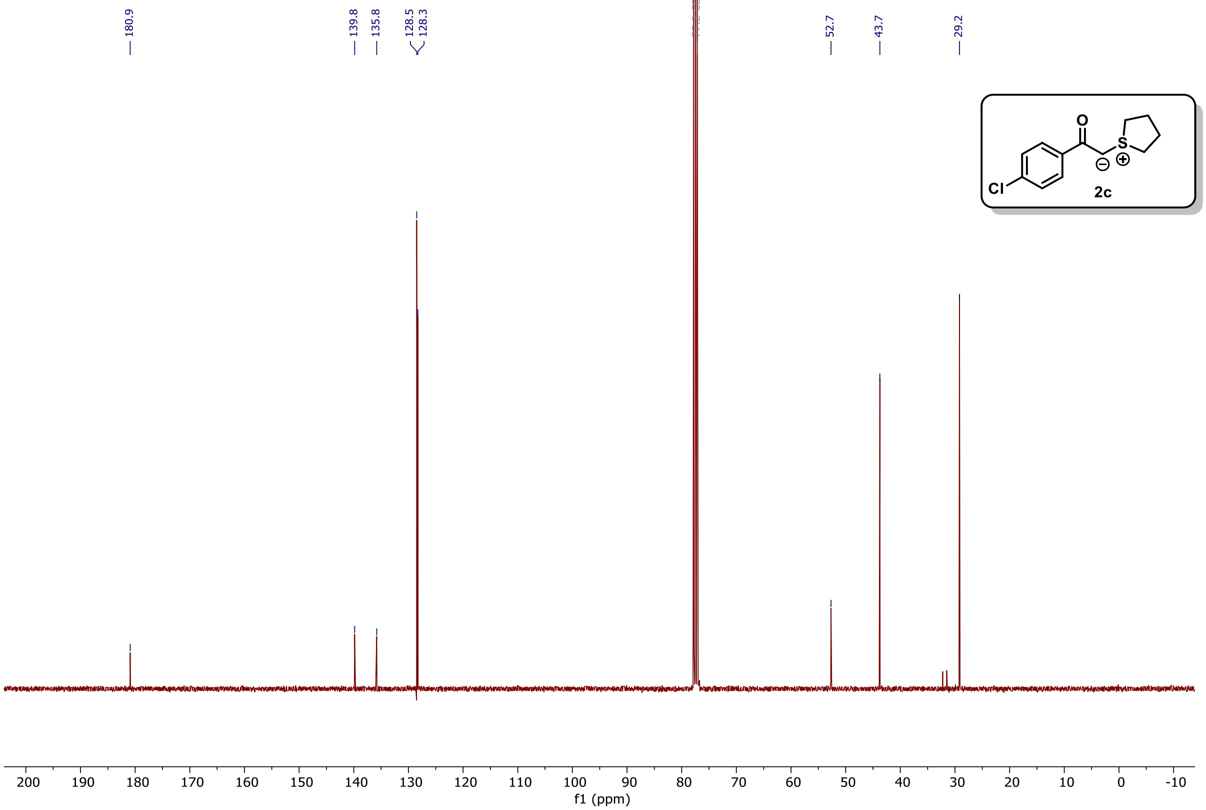


**$^{13}C\{^{1}H\}$-NMR** (101 MHz, $CDCl_3$)

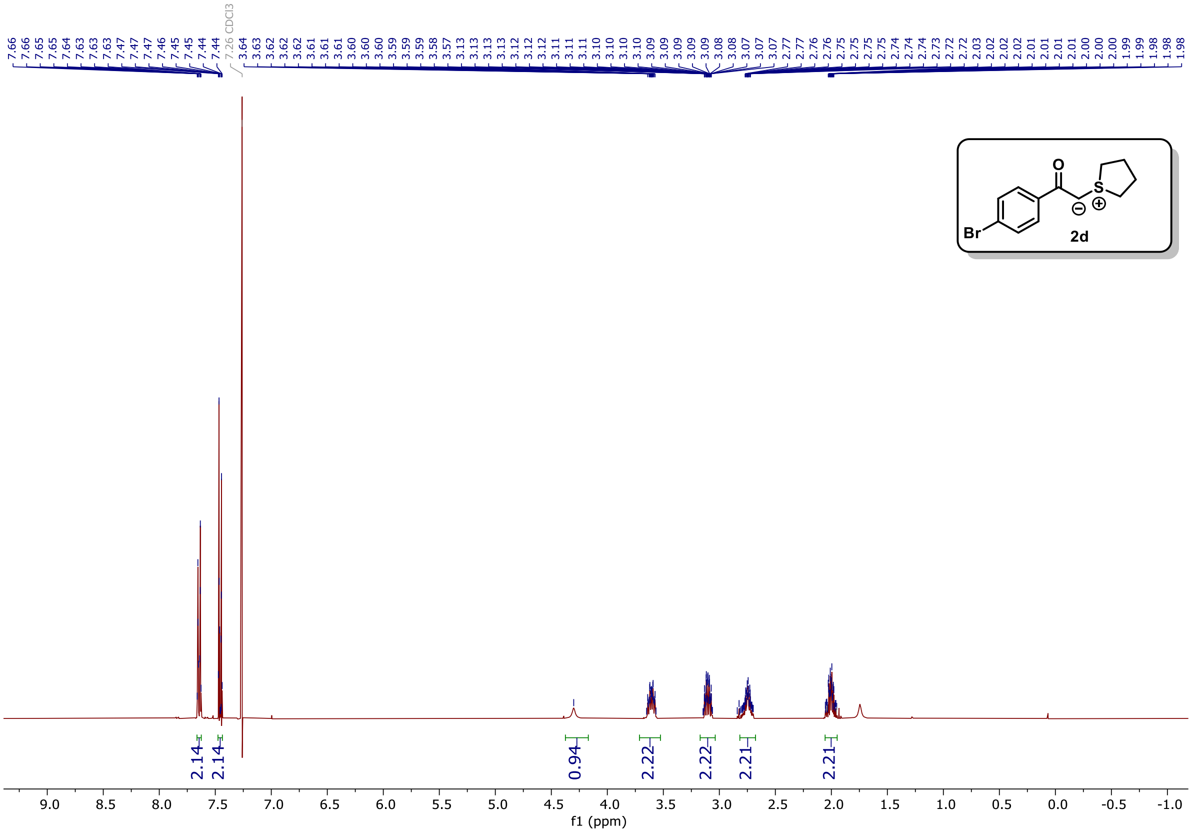


$^1$**H-NMR** (400 MHz, $CDCl_3$)

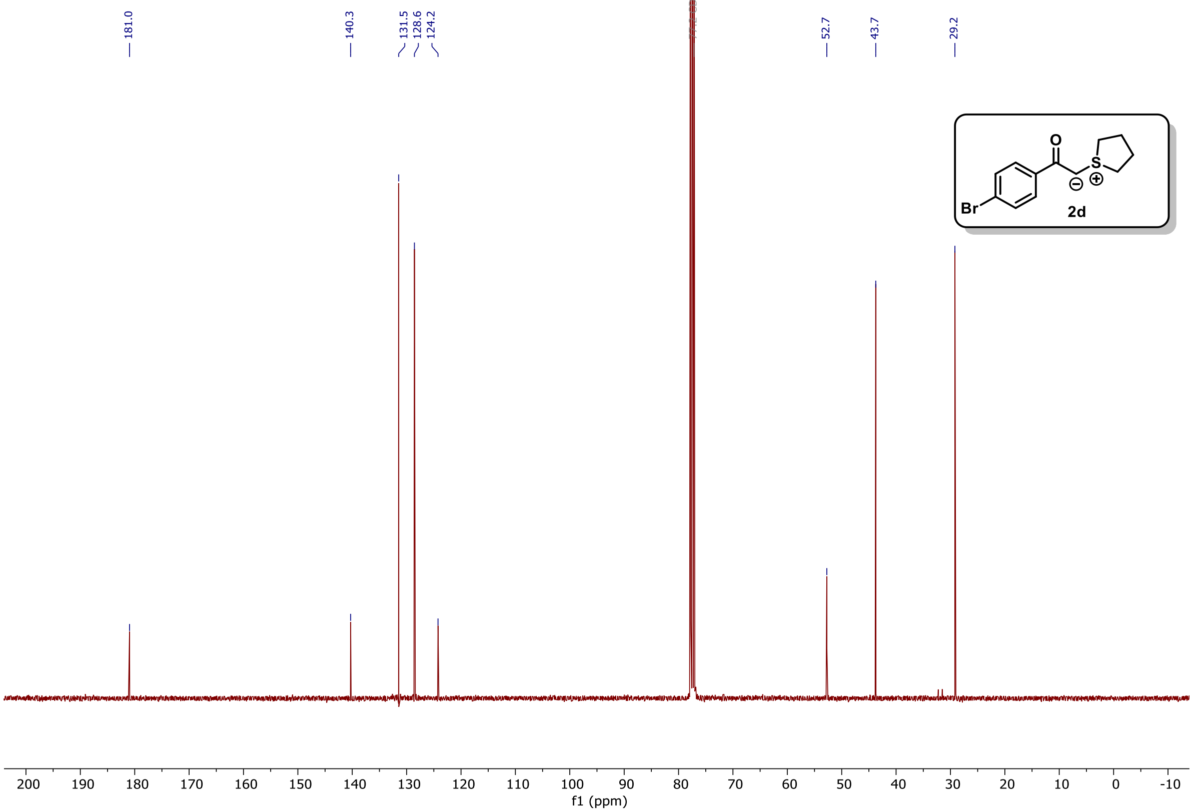


$^{13}$**C{$^1$H}-NMR** (101 MHz, $CDCl_3$)

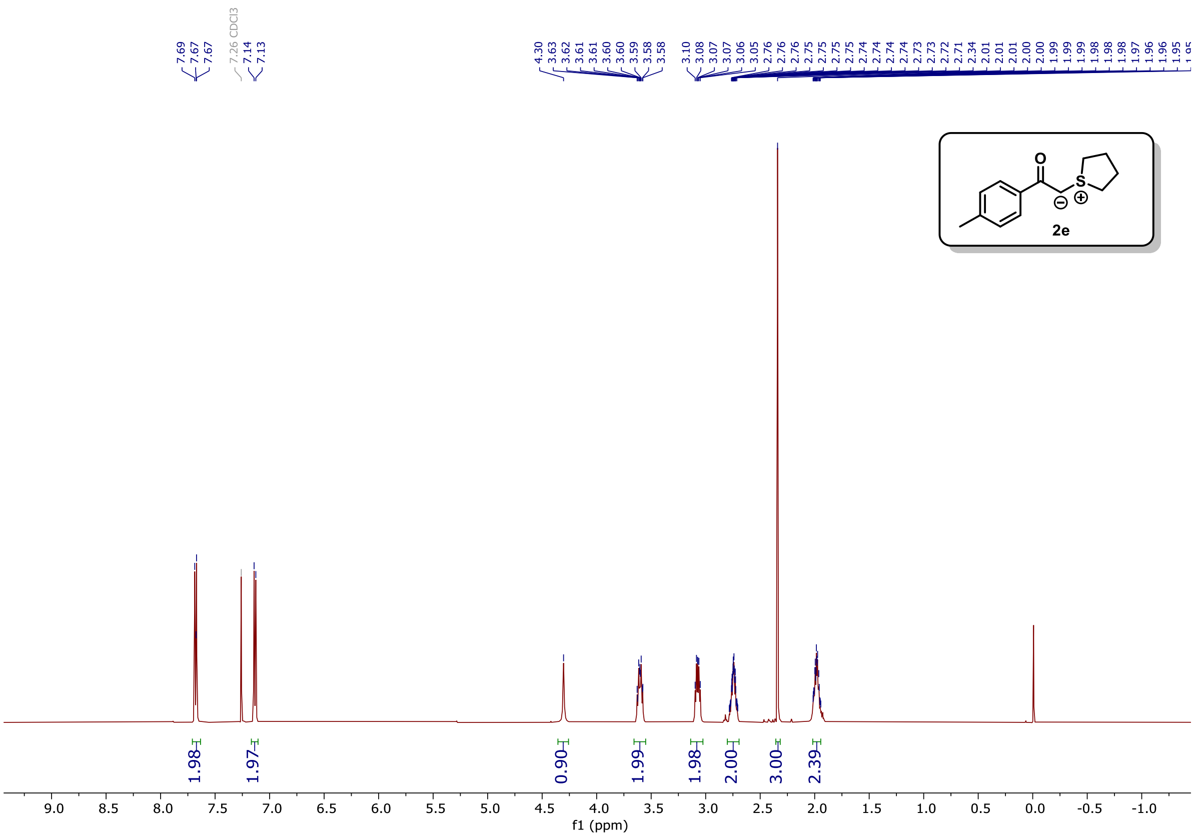


**$^{1}$H-NMR** (500 MHz, $CDCl_3$)

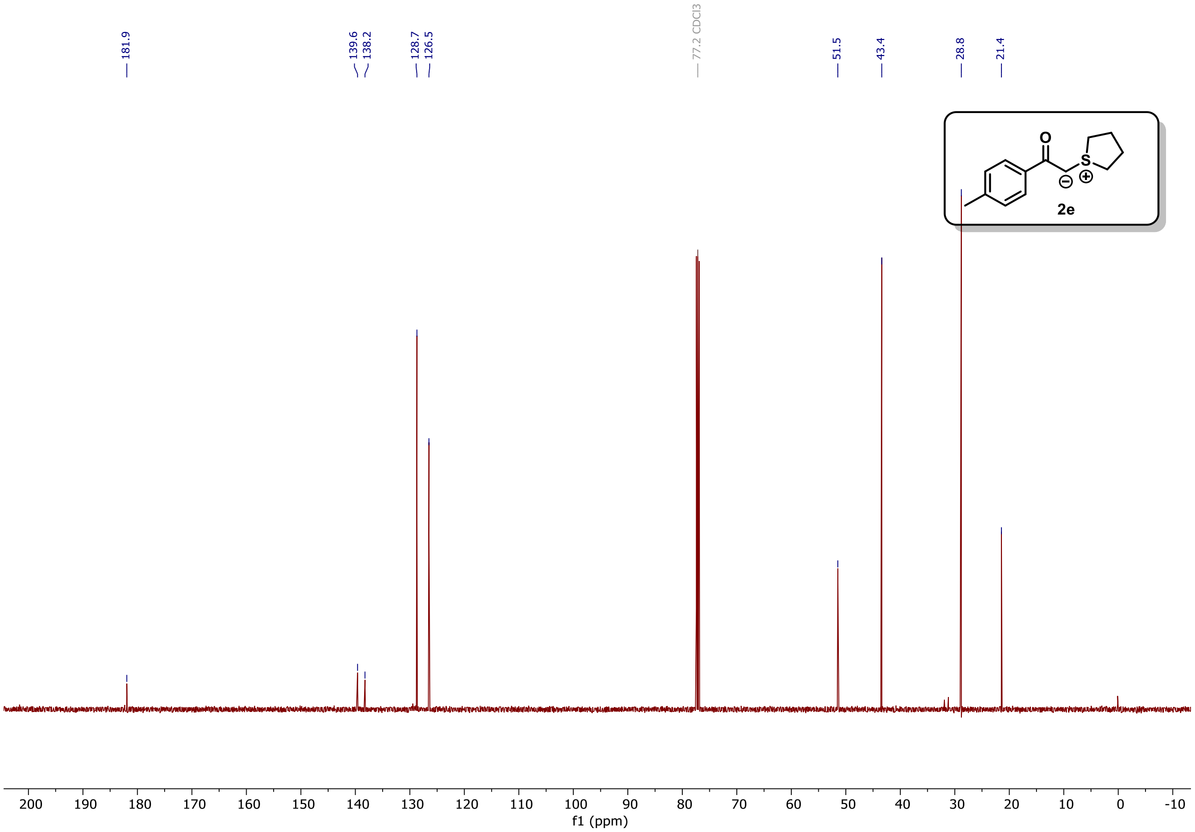


**$^{13}$C{$^{1}$H}-NMR** (126 MHz, $CDCl_3$)

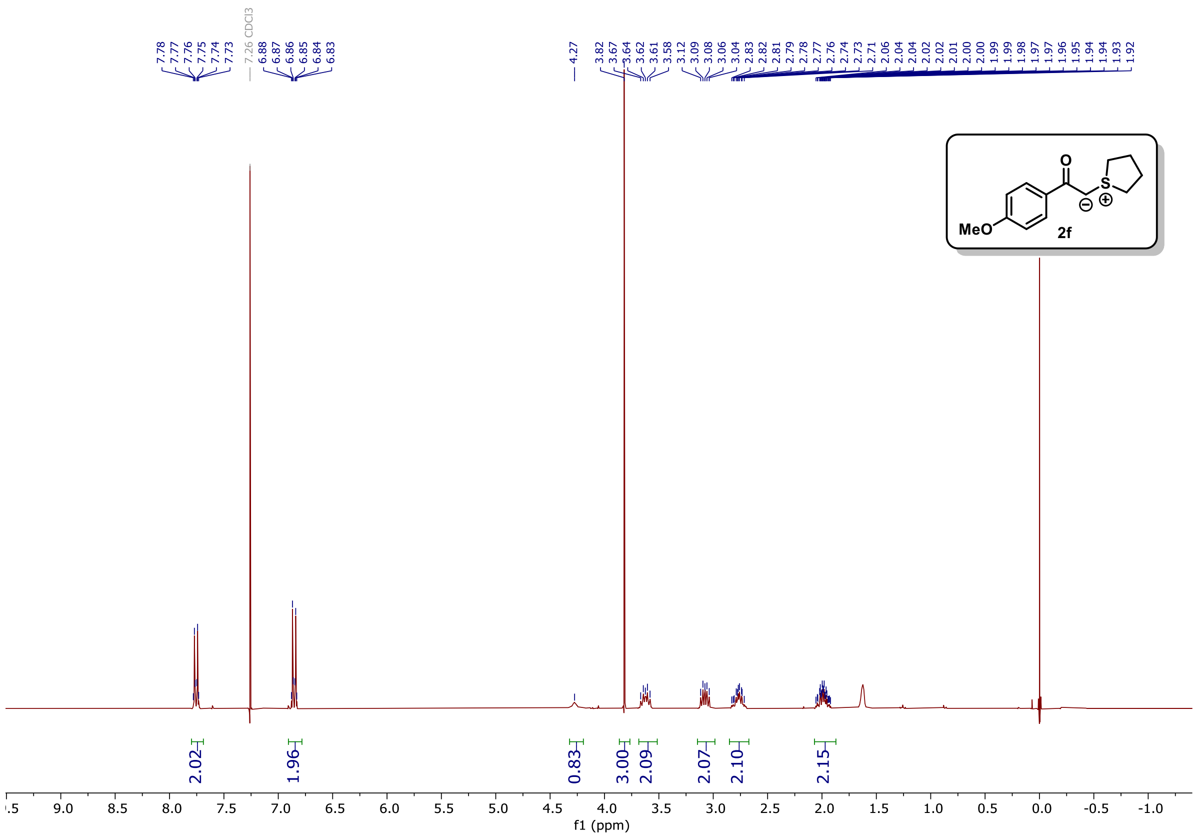


**¹H-NMR** (300 MHz, CDCl₃)

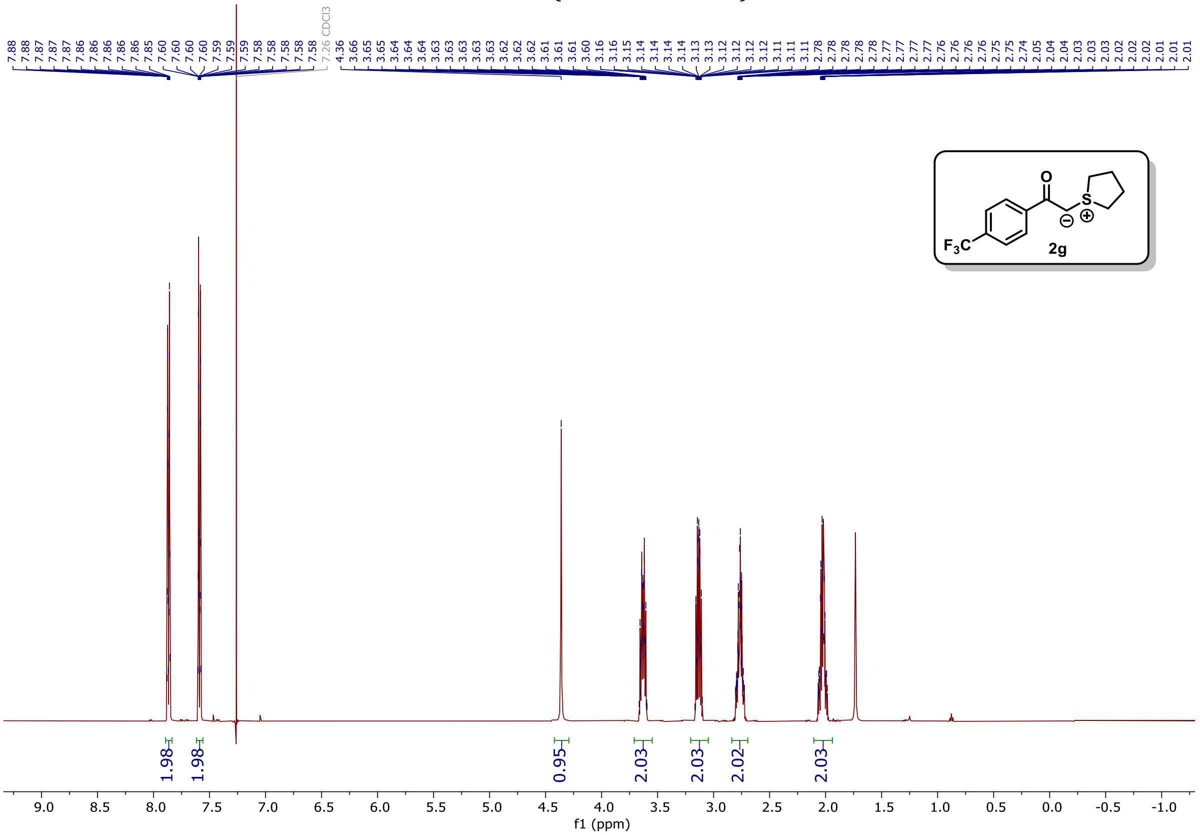


**¹H-NMR** (500 MHz, CDCl₃)

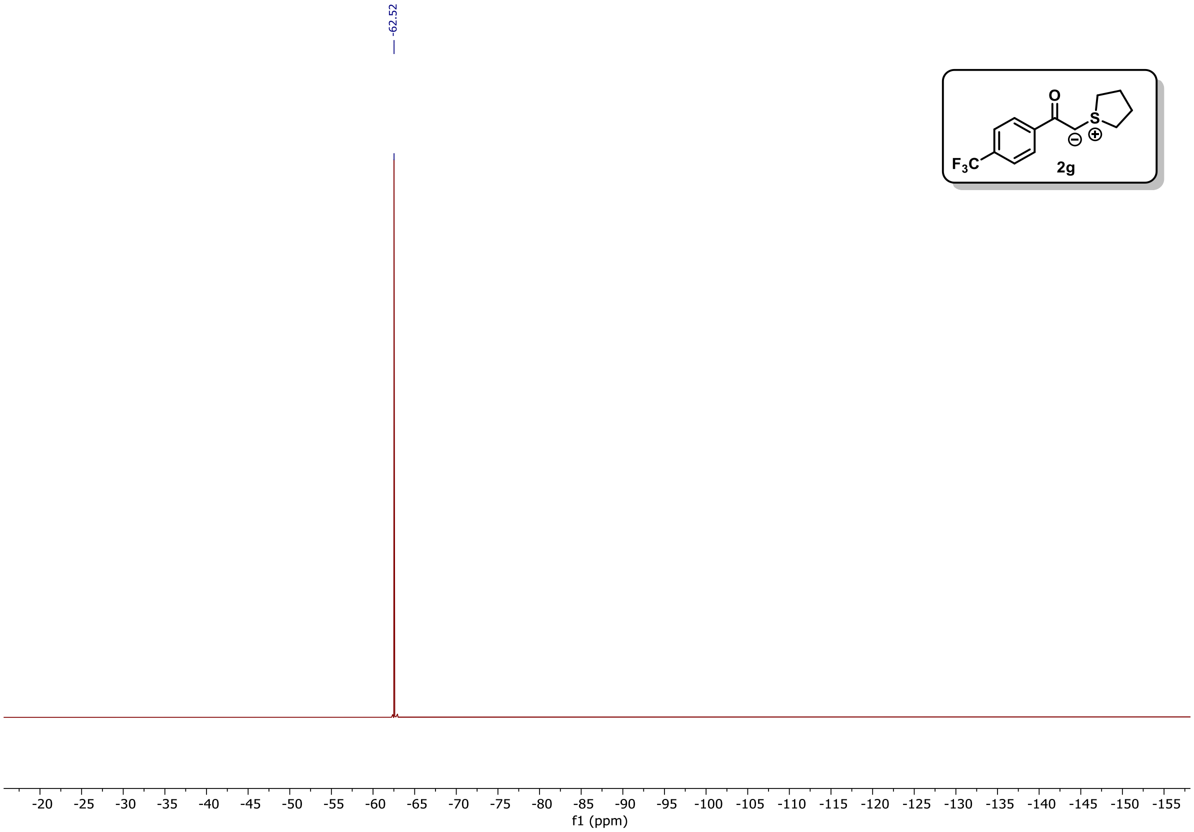


**$^{19}$F-NMR** (471 MHz, $CDCl_3$)

**$^{13}$C NMR:** Sulfonium ylide **2f**, **2g** shows low stability in solution and undergoes decomposition. Therefore, it was not possible to obtain a clean **$^{13}$C{$^{1}$H}-NMR** spectrum.

# NMR Spectra of Cyclobutanones 3a-o

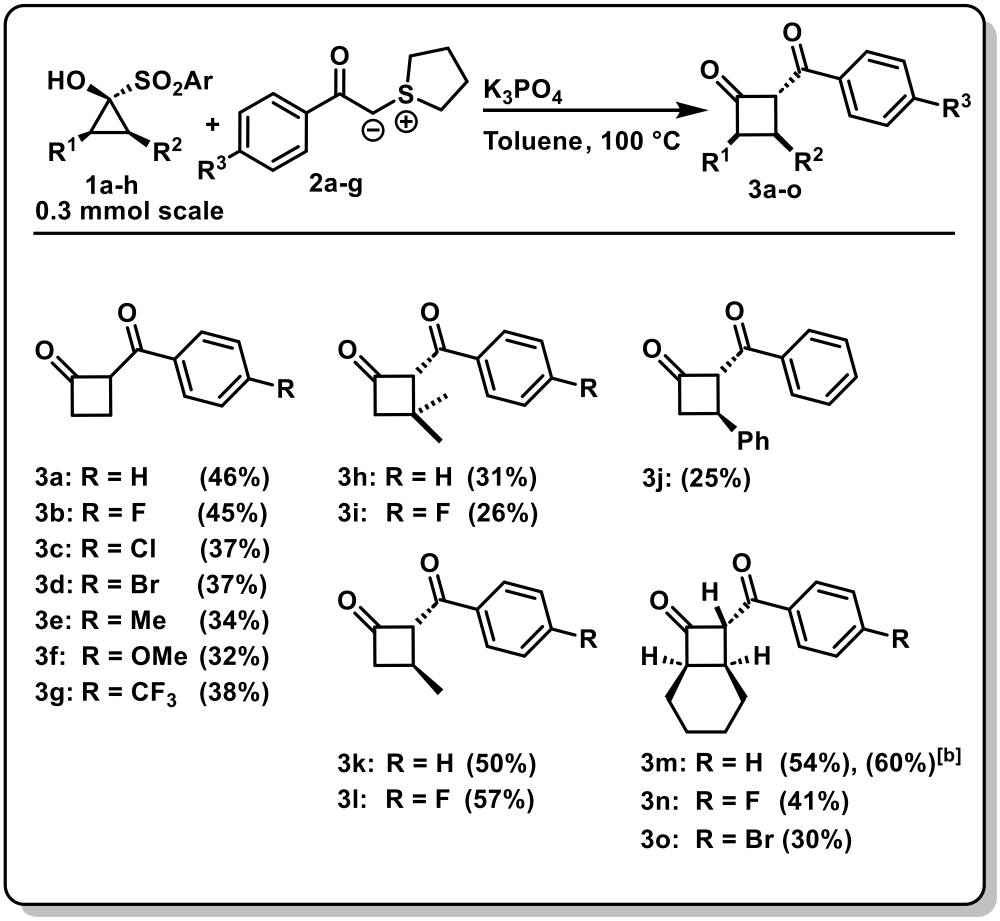

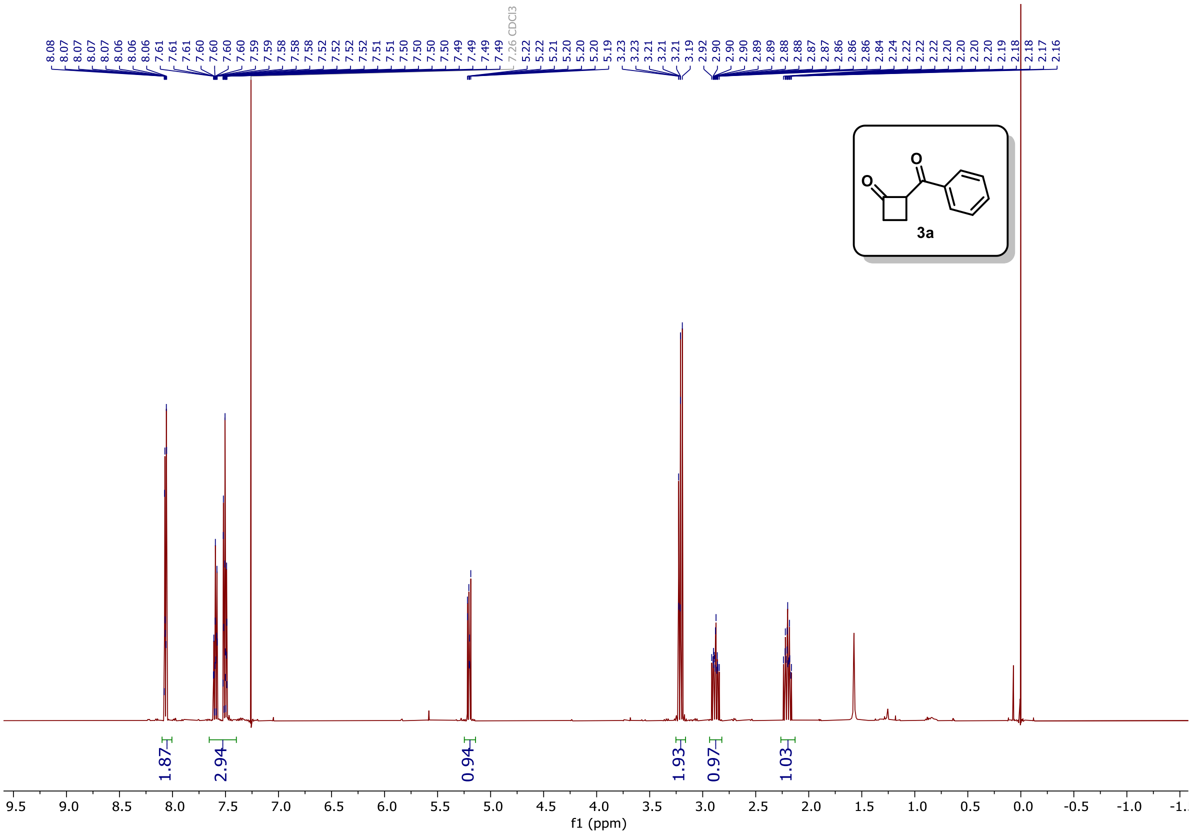

**¹H-NMR** (500 MHz, CDCl₃)

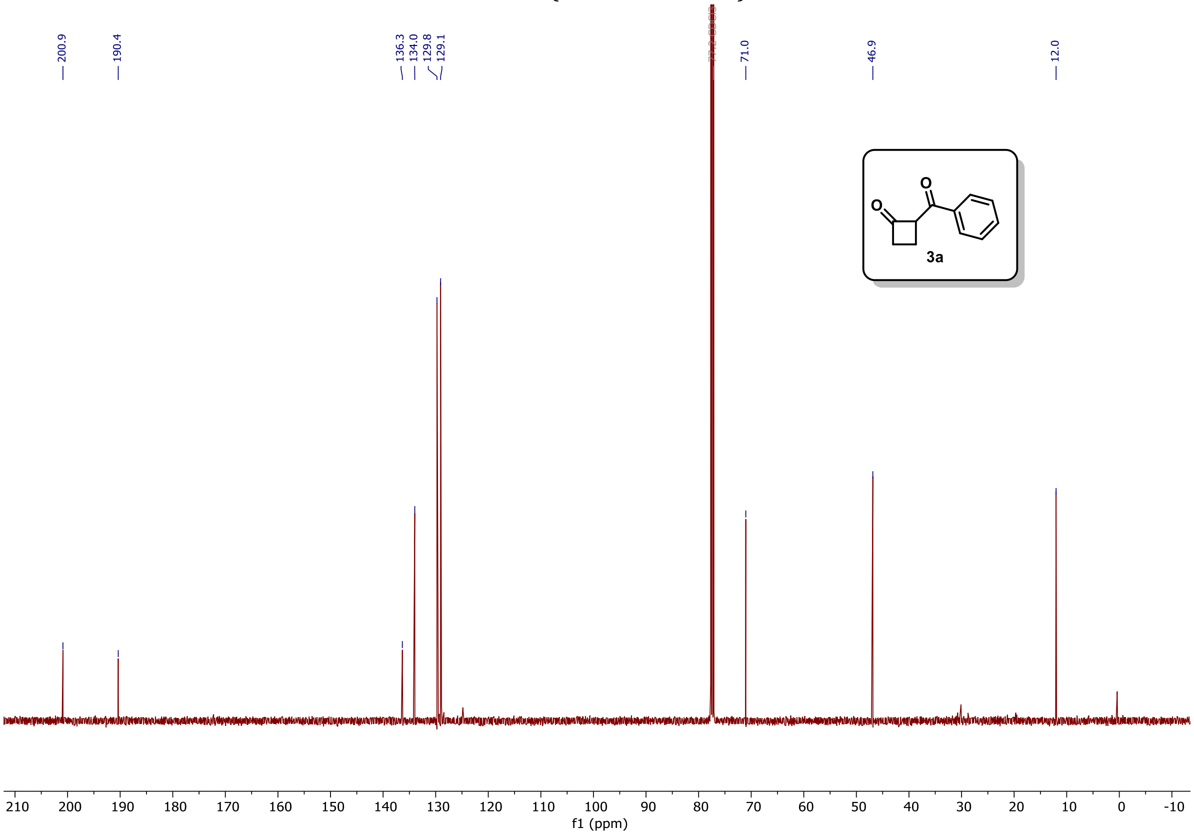

**¹³C{¹H}-NMR** (126 MHz, CDCl₃)

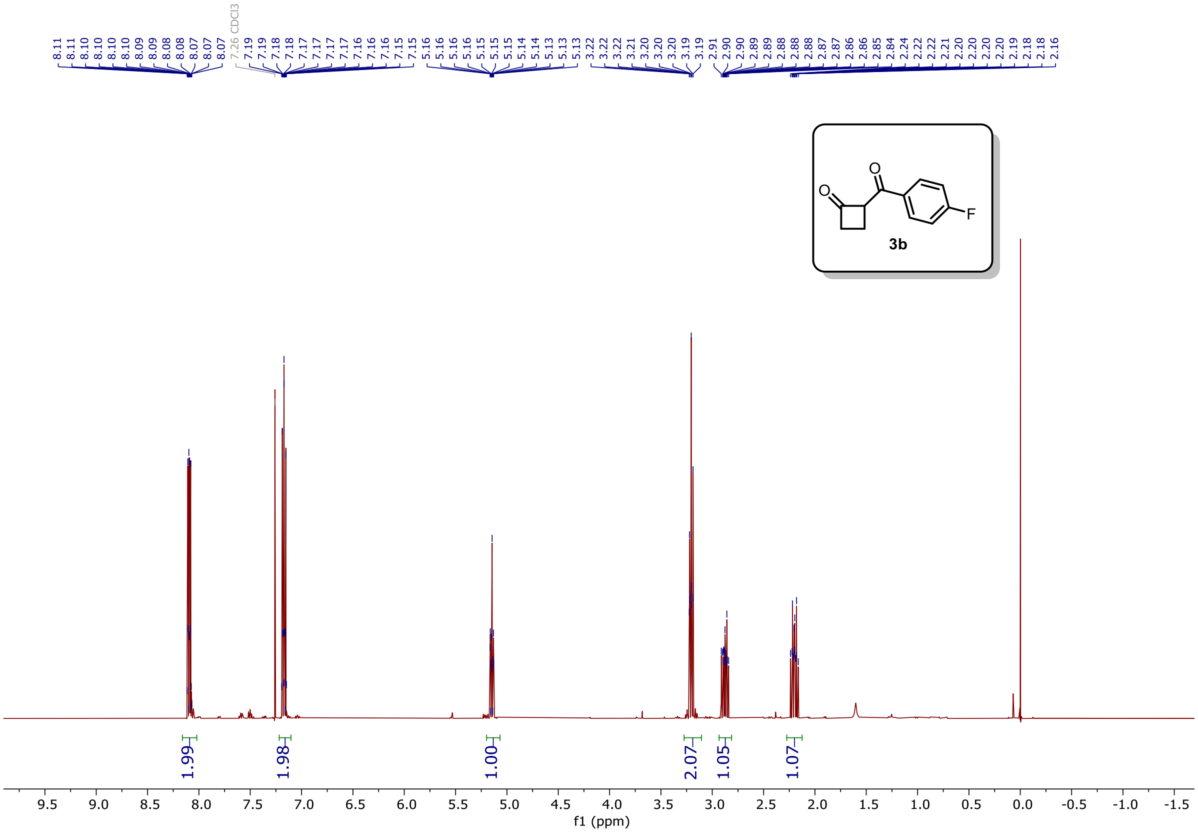


**¹H-NMR** (500 MHz, $CDCl_3$)

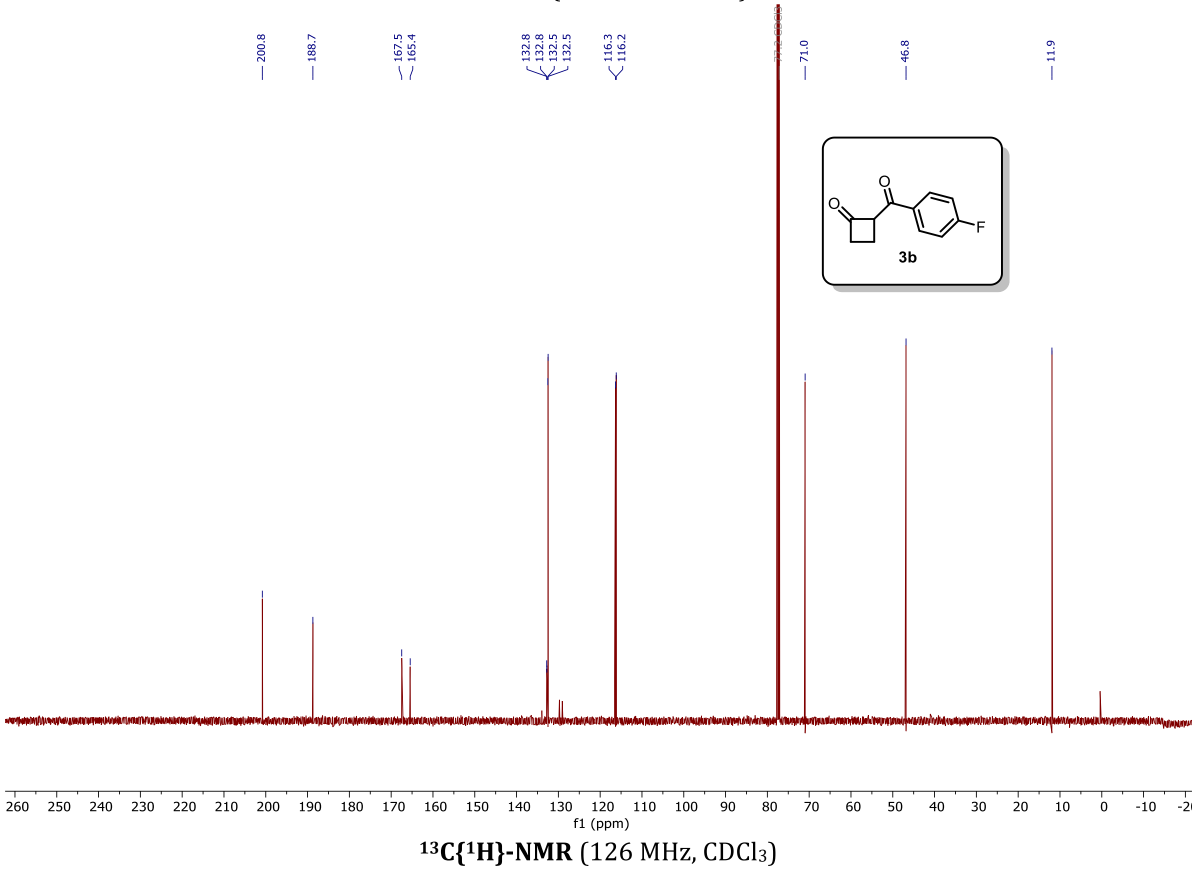


**¹³C{¹H}-NMR** (126 MHz, $CDCl_3$)

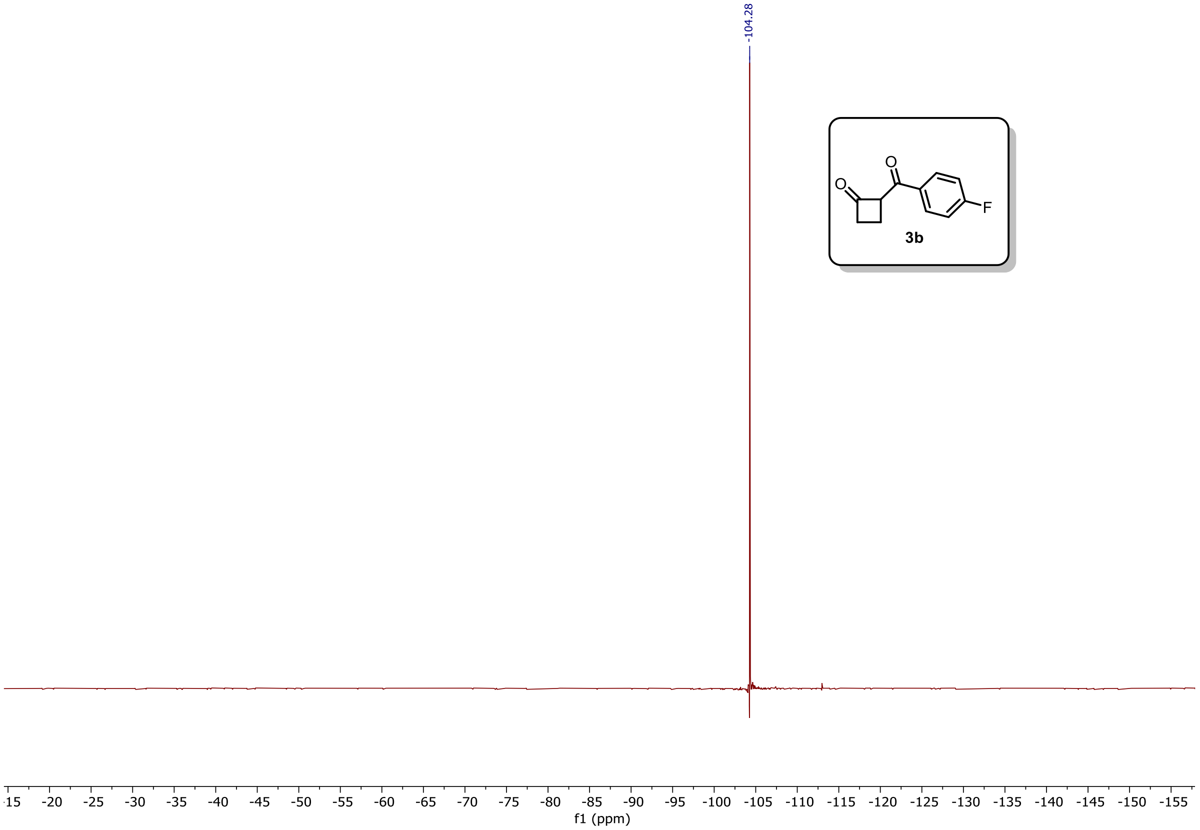


**$^{19}$F NMR** (471 MHz, $CDCl_3$)

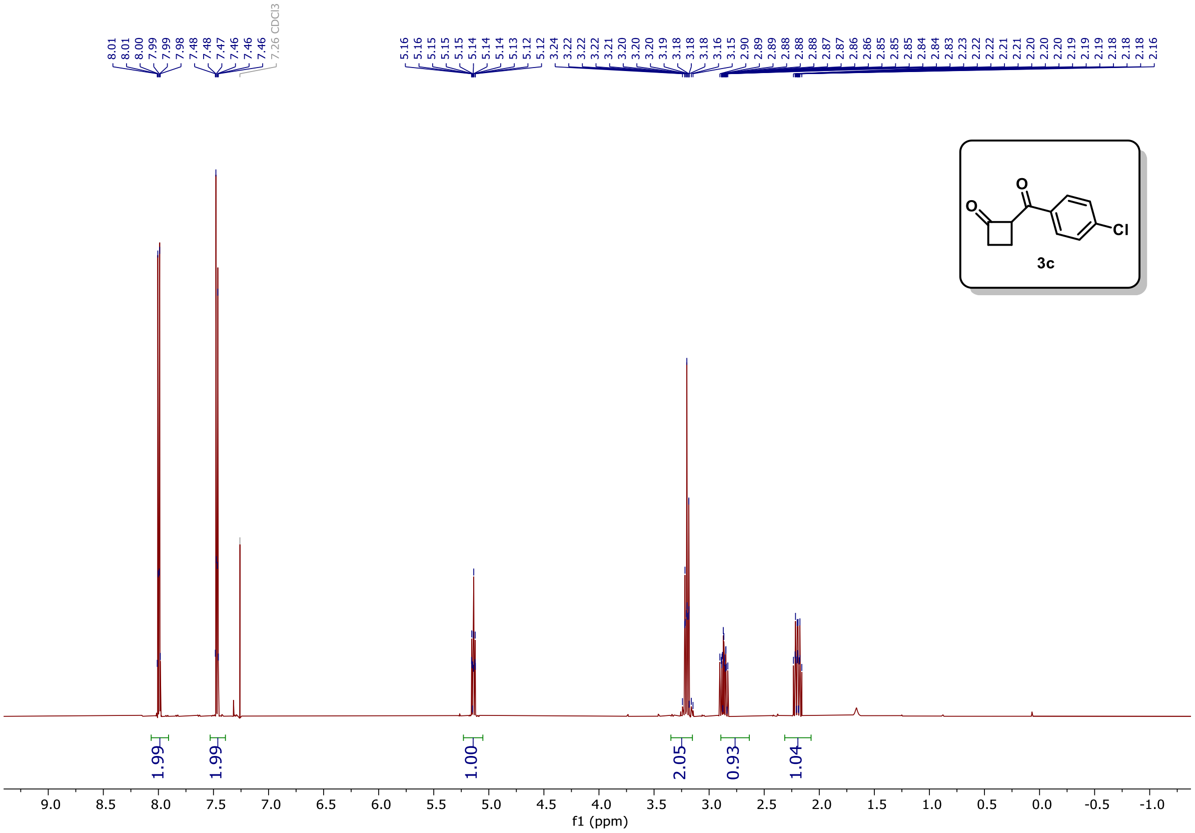


**$^{1}$H-NMR** (500 MHz, $CDCl_3$)

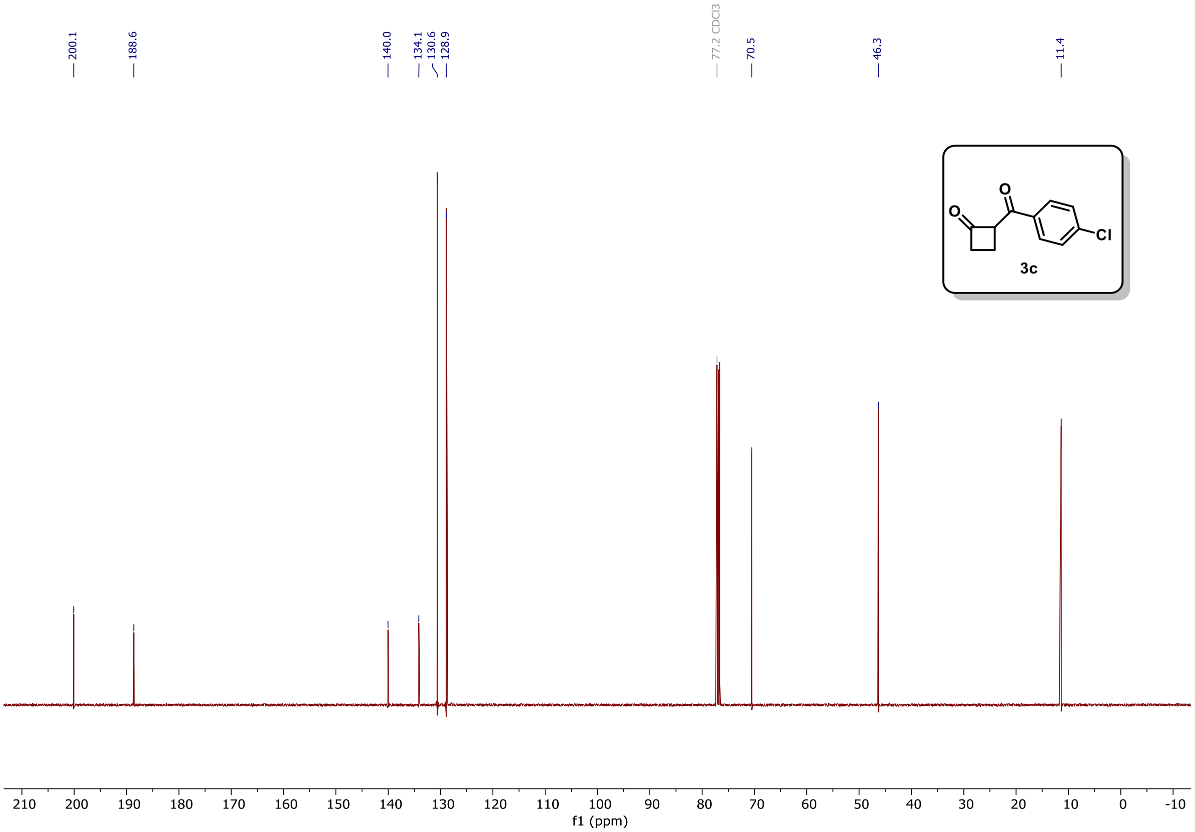


**$^{13}$C{$^{1}$H}-NMR** (126 MHz, $CDCl_3$)

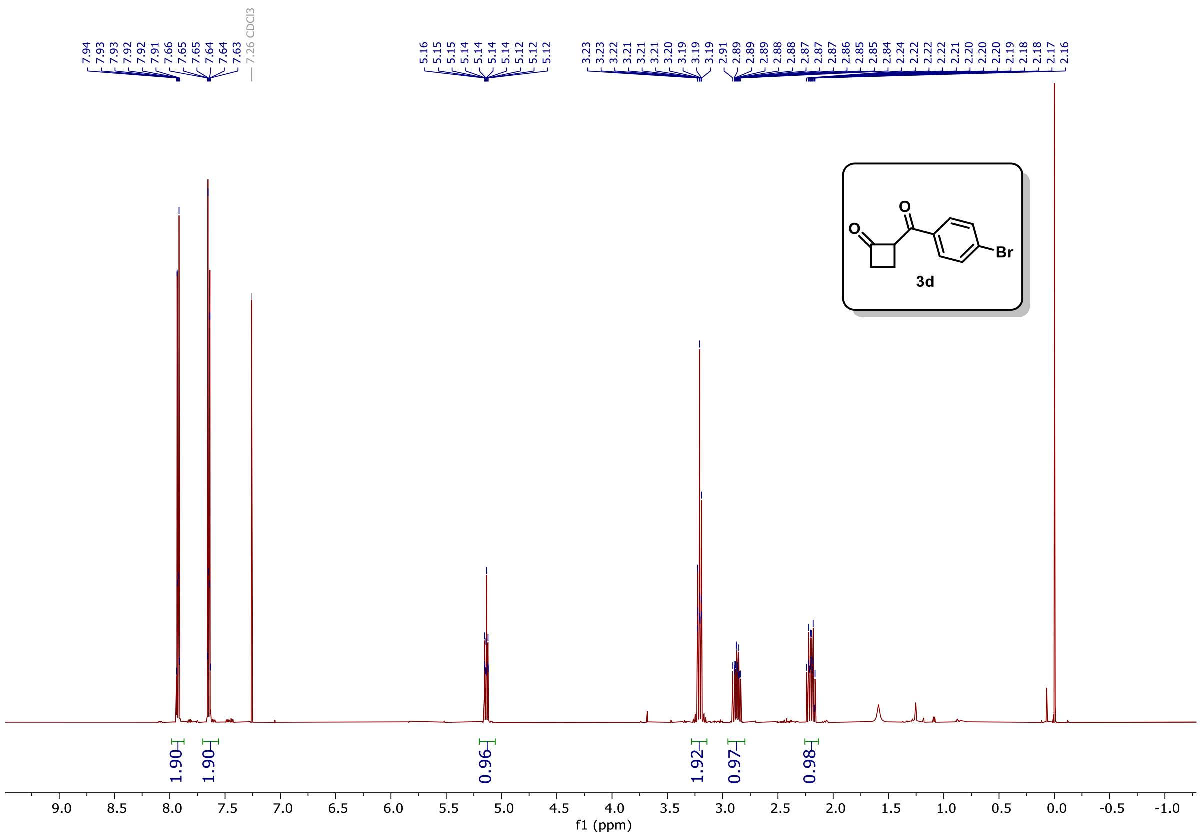


**$^{1}$H-NMR** (500 MHz, $CDCl_3$)

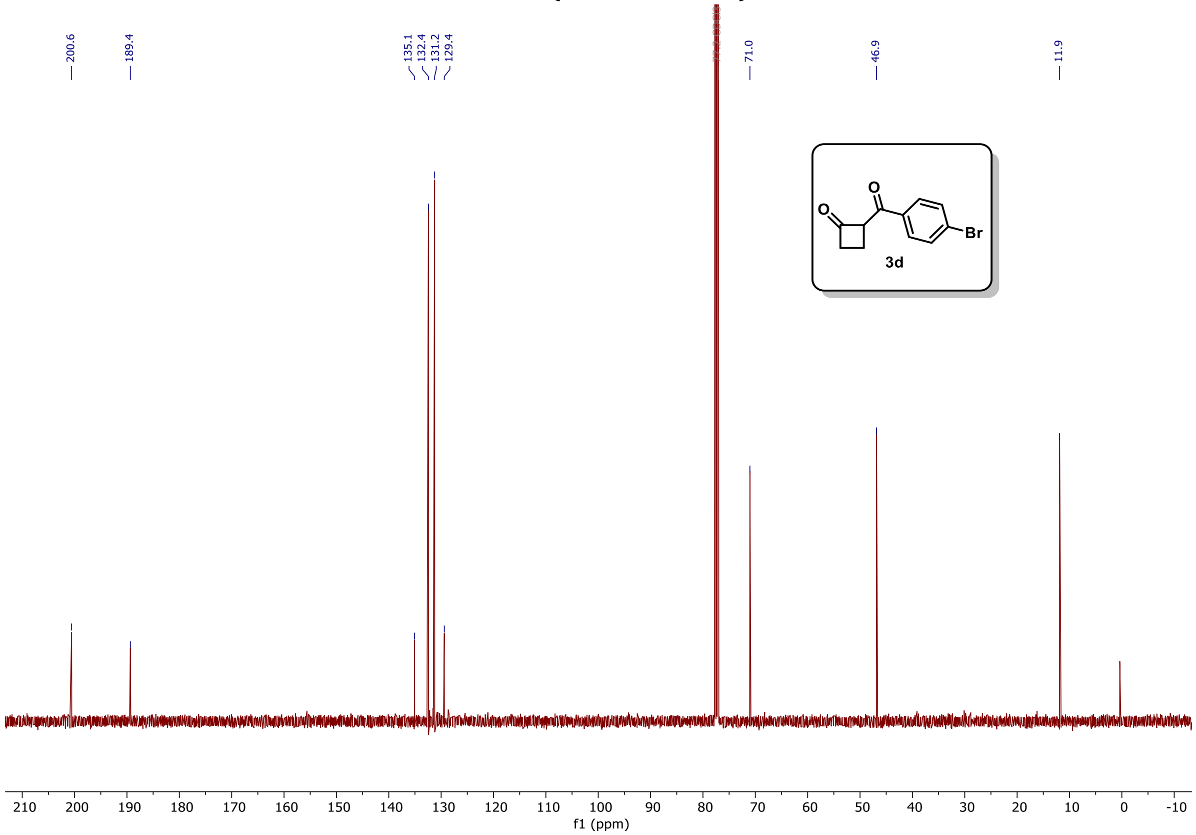


**$^{13}$C{$^{1}$H}-NMR** (126 MHz, $CDCl_3$)

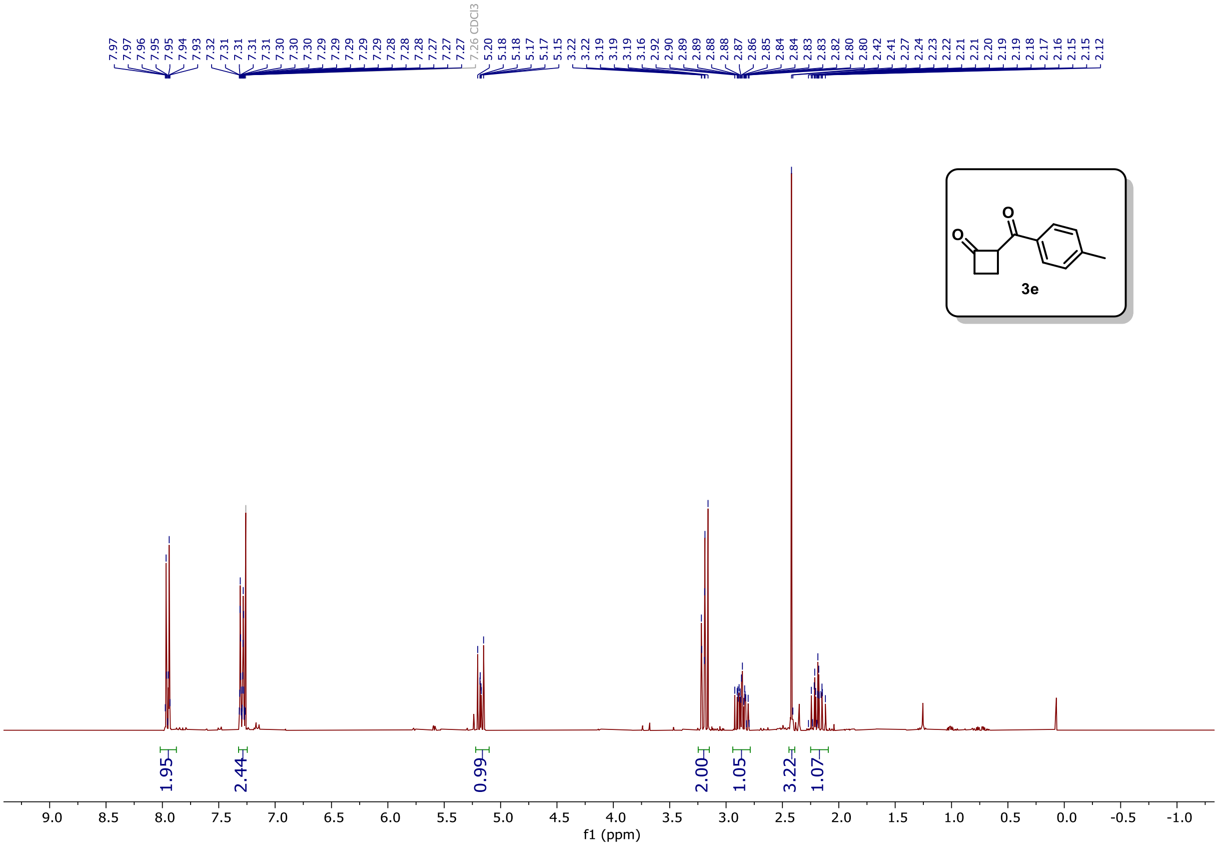


**$^{1}$H-NMR** (300 MHz, $CDCl_3$)

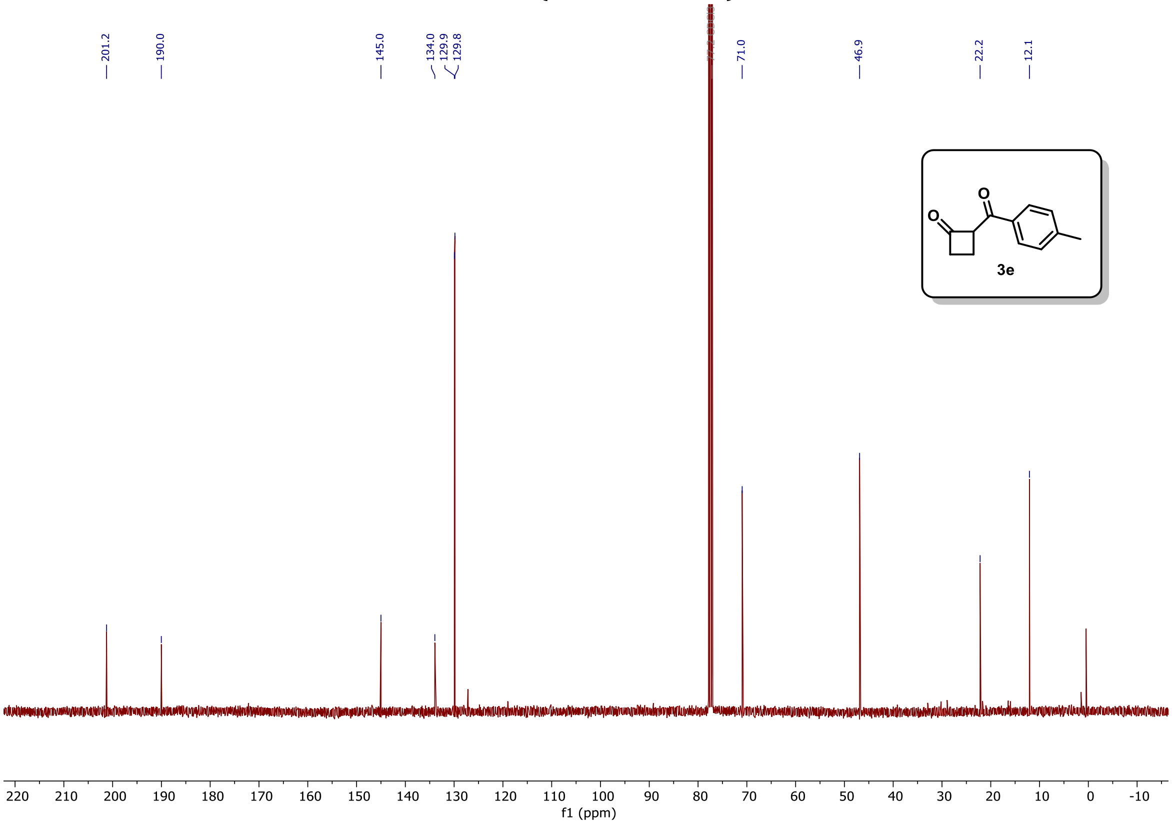


**$^{13}$C{$^{1}$H}-NMR** (101 MHz, $CDCl_3$)

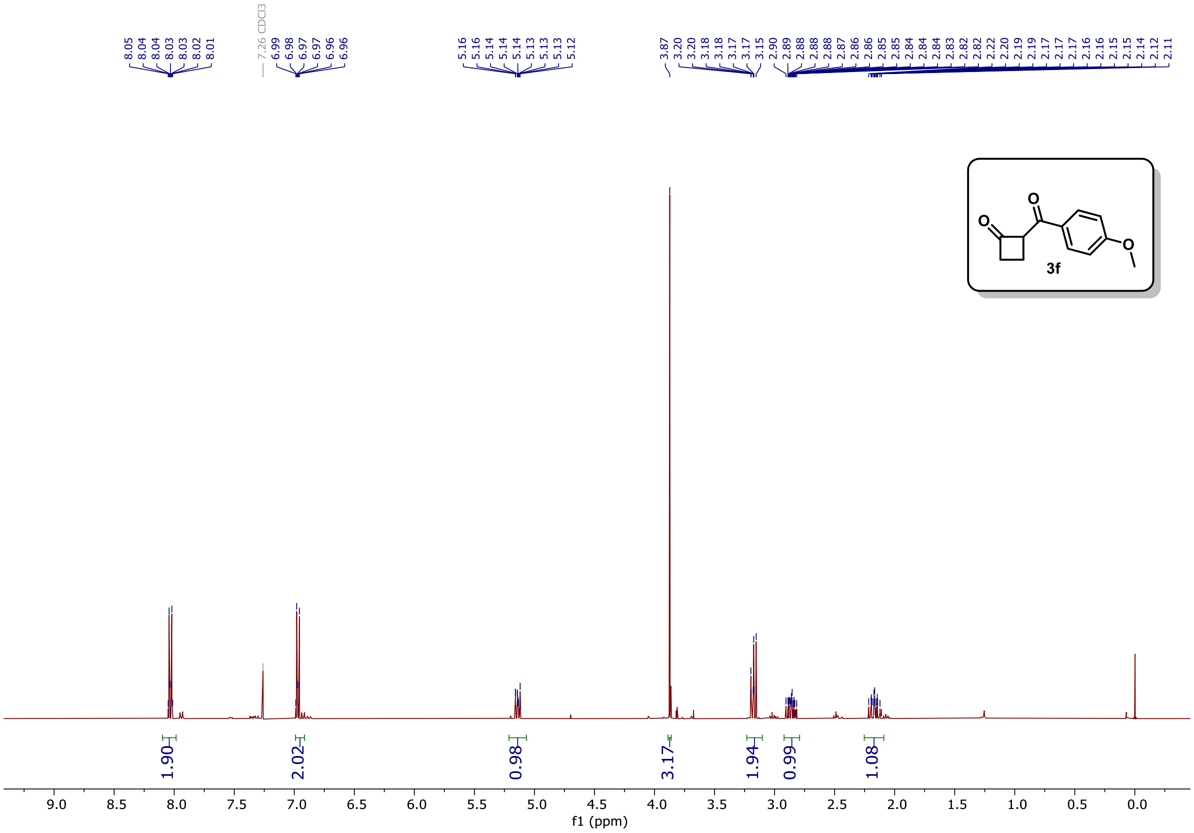


**¹H-NMR** (400 MHz, $CDCl_3$)

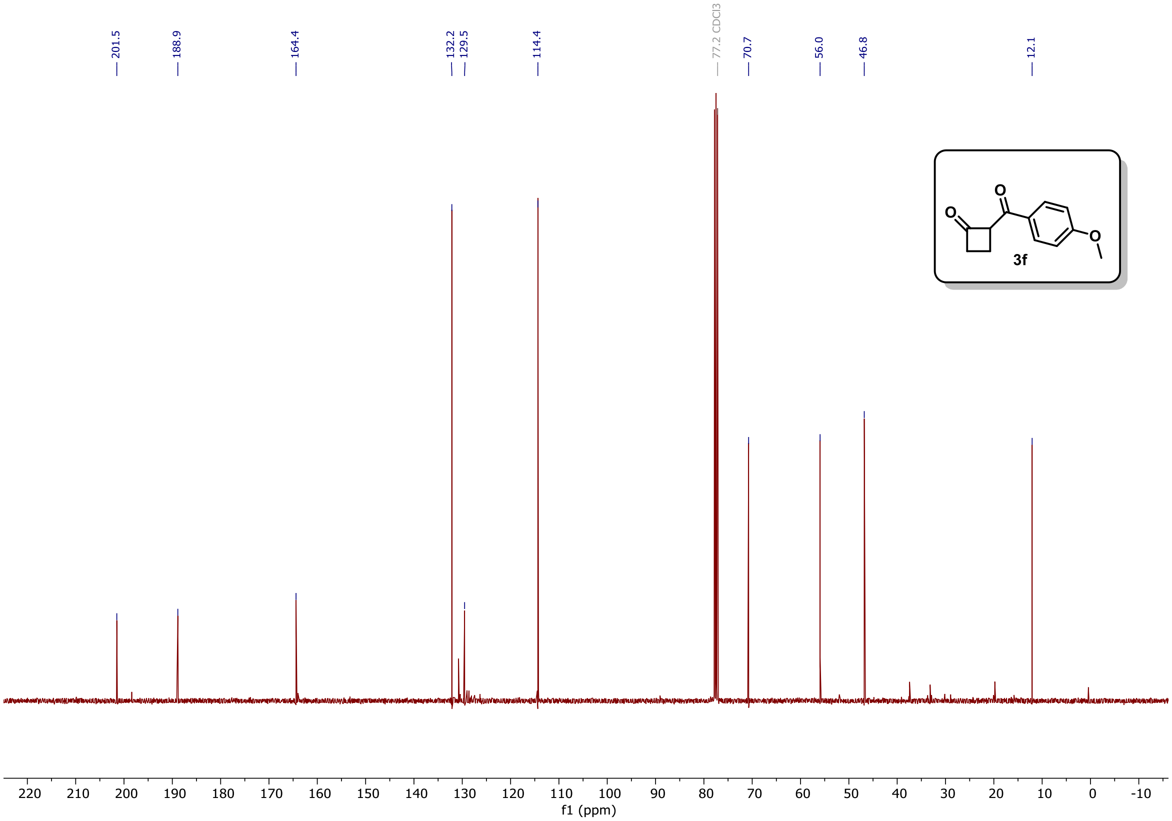


**$^{13}C\{^1H\}$-NMR** (101 MHz, $CDCl_3$)

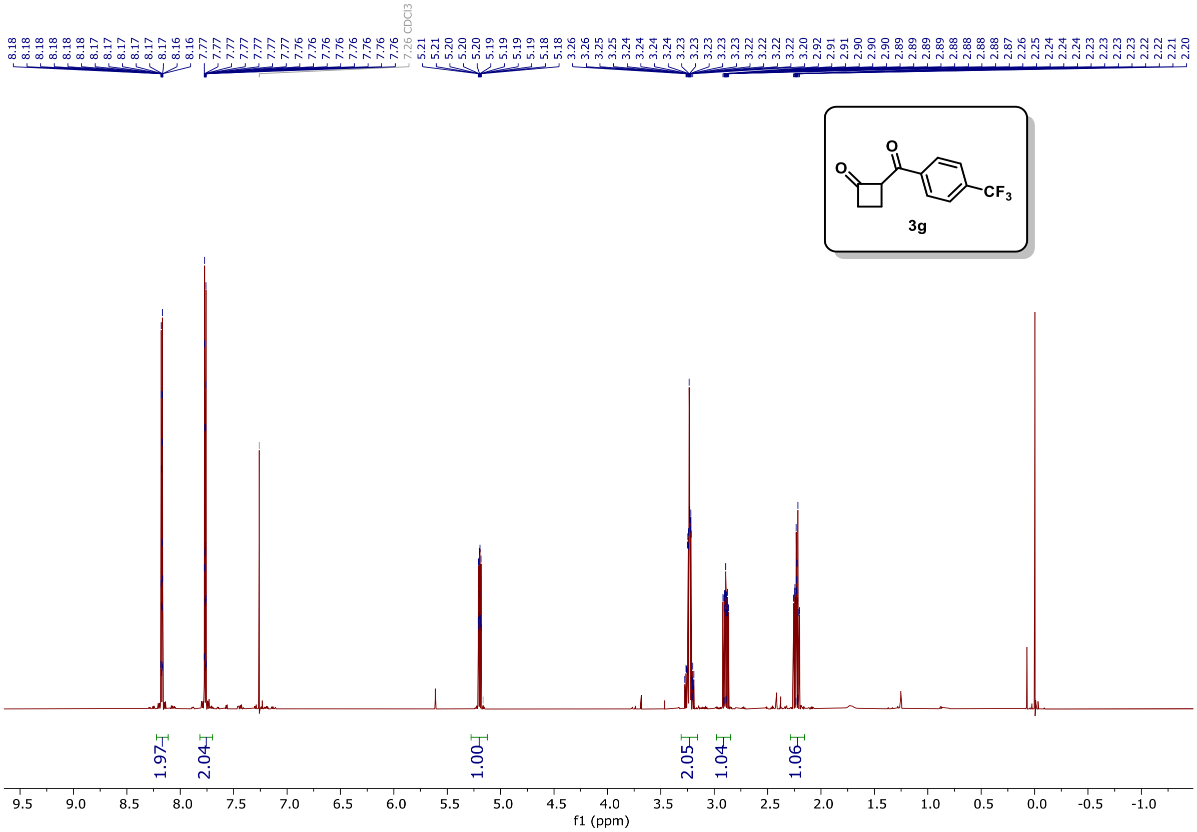


**$^{1}$H NMR** (700 MHz, $CDCl_3$)

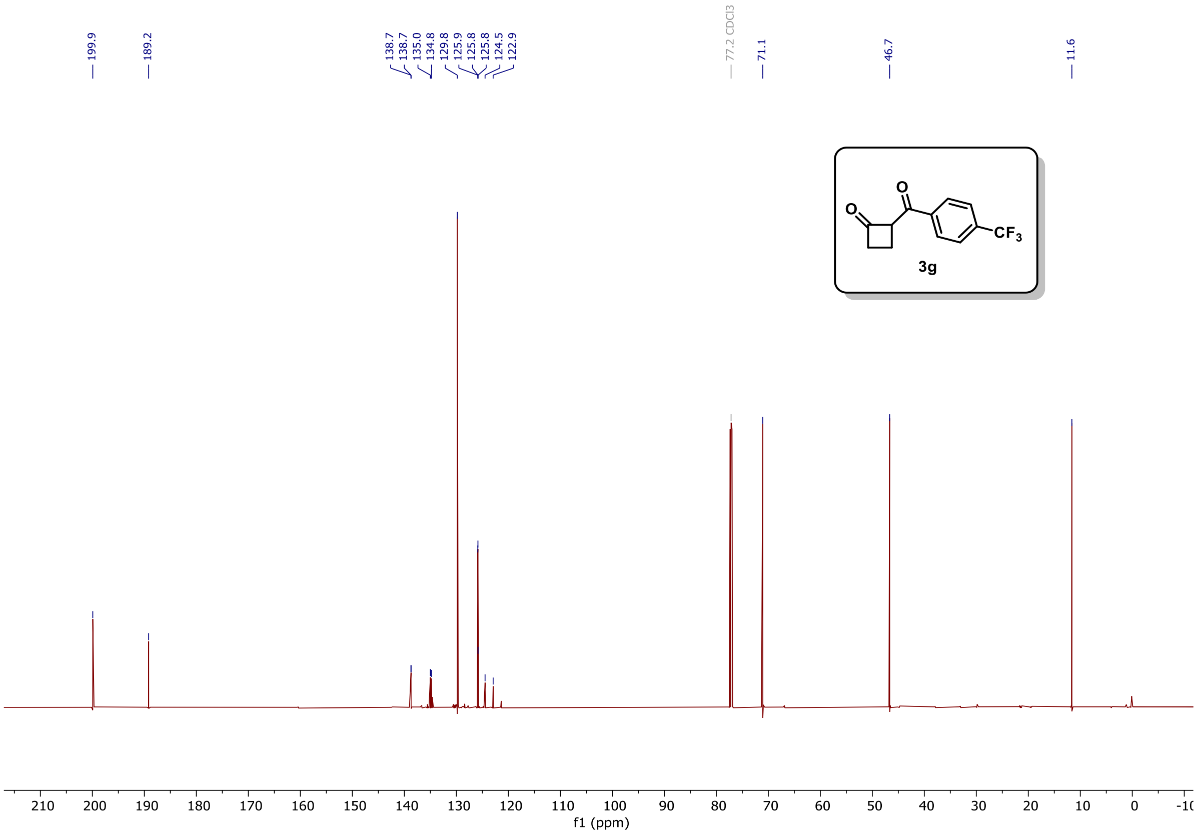


**$^{13}$C{$^{1}$H}-NMR** (176 MHz, $CDCl_3$)

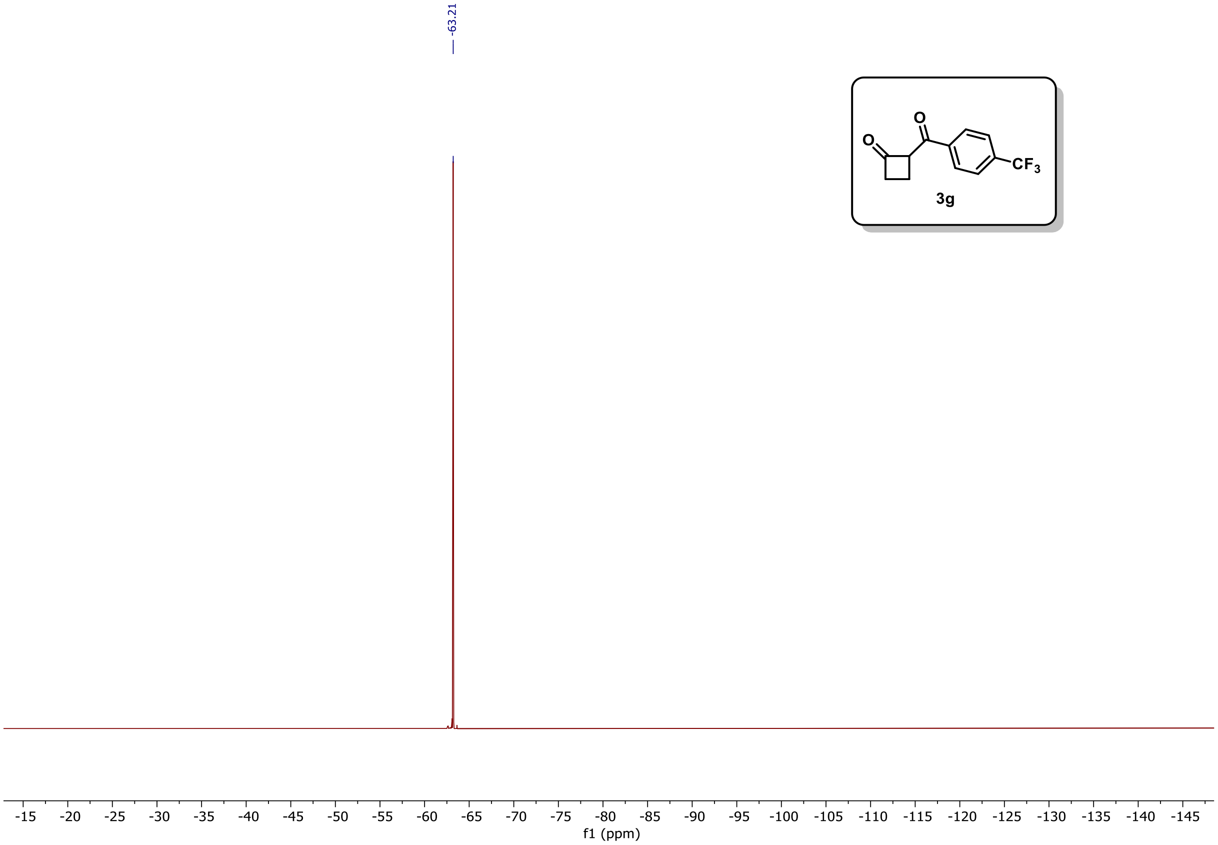


**$^{19}F$ NMR** (471 MHz, $CDCl_3$)

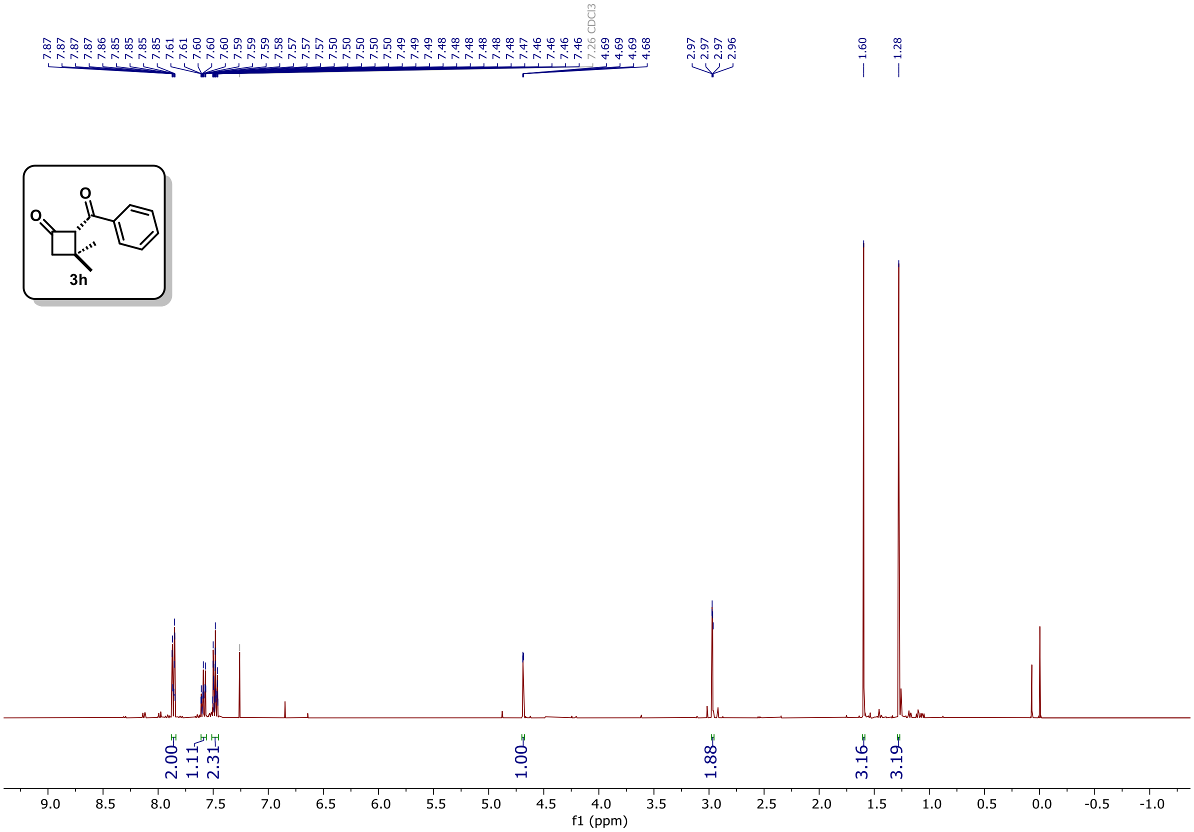


**¹H-NMR** (400 MHz, $CDCl_3$)

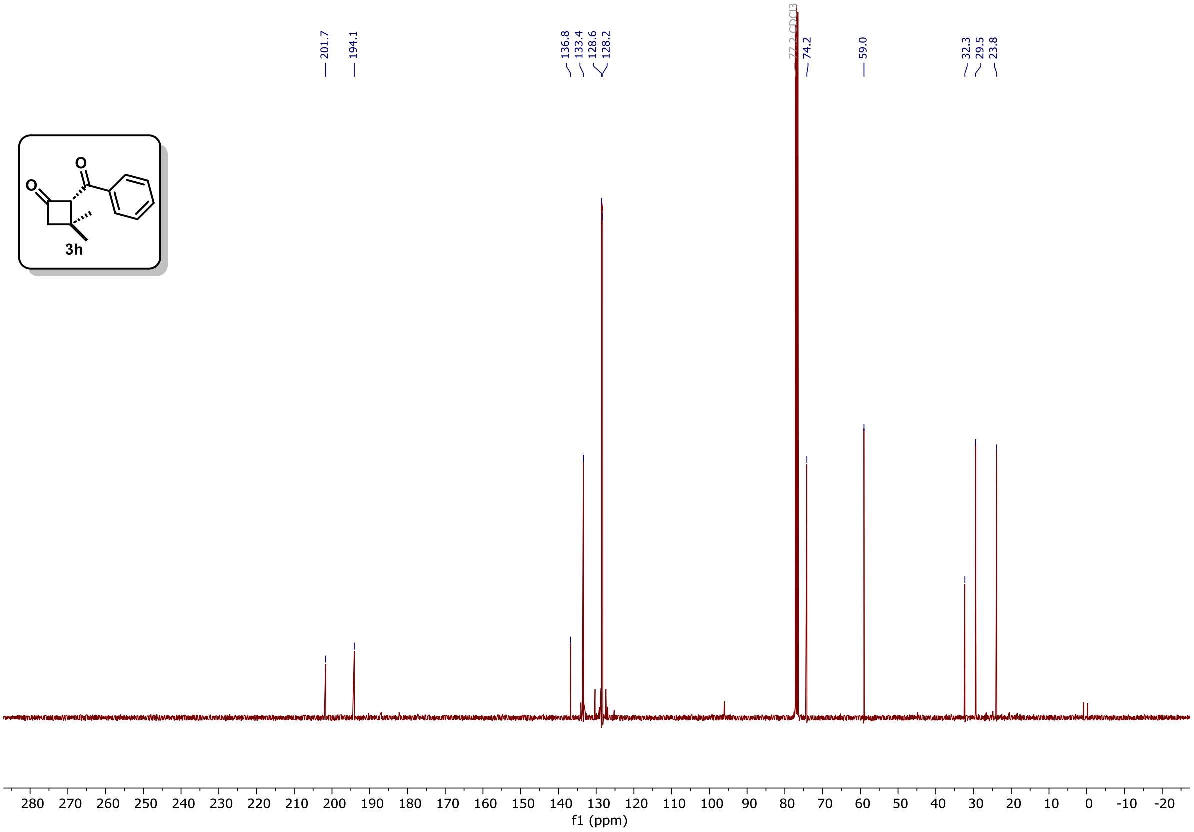


**$^{13}$C{$^1$H}-NMR** (101 MHz, $CDCl_3$)

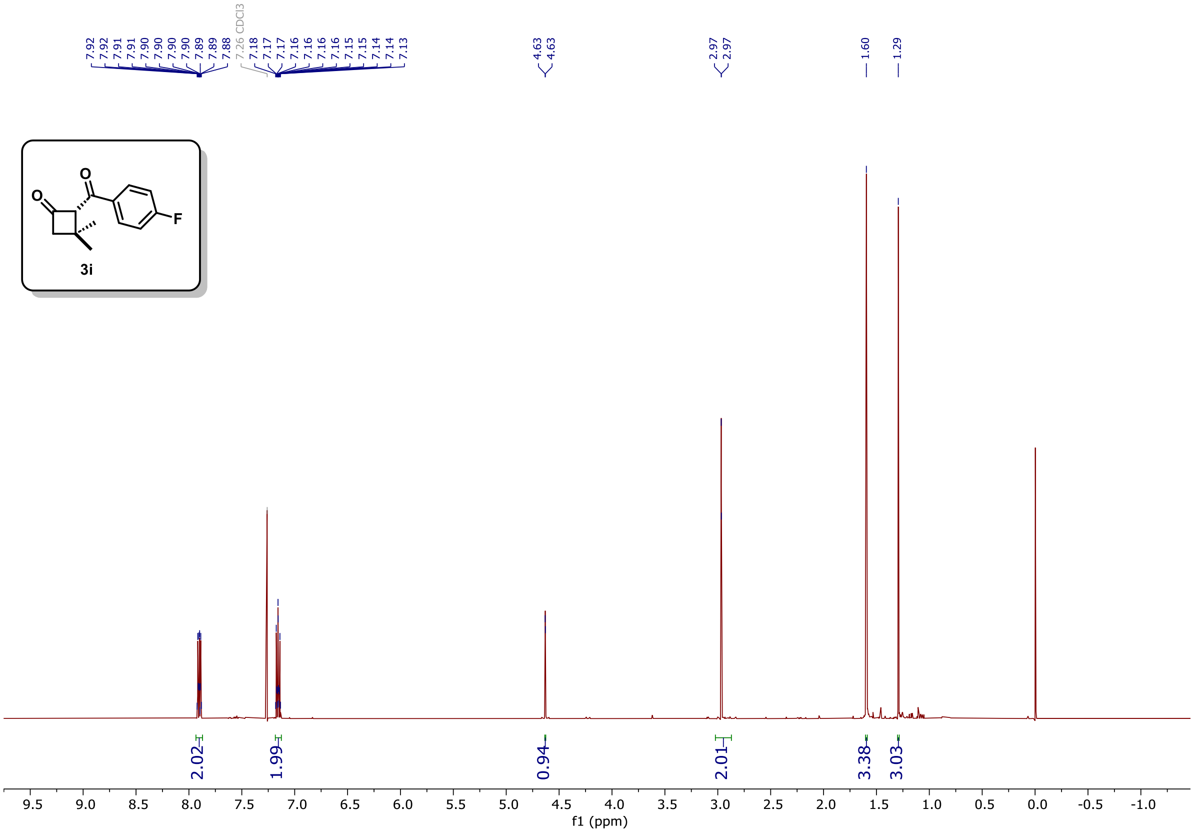


**$^1$H-NMR** (500 MHz, $CDCl_3$)

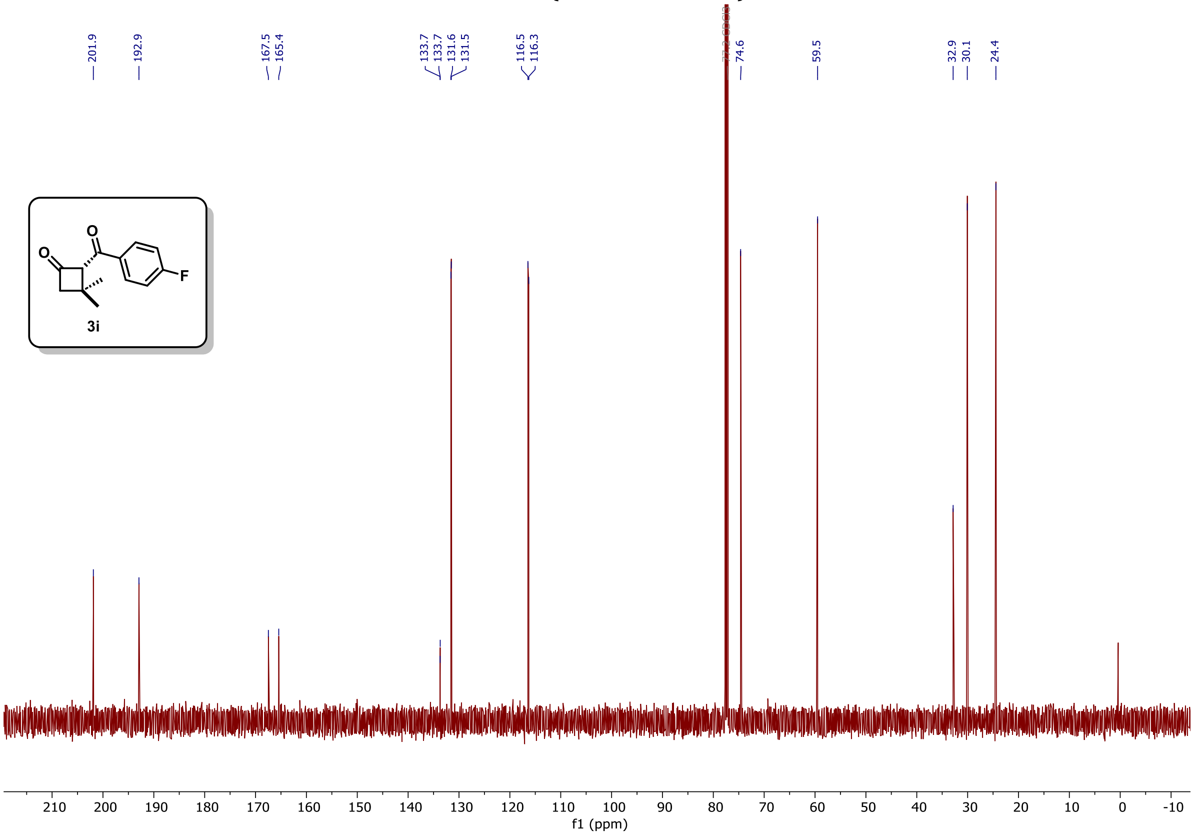


**$^{13}C\{^1H\}$-NMR** (126 MHz, $CDCl_3$)

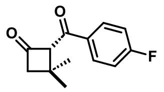

**3i**

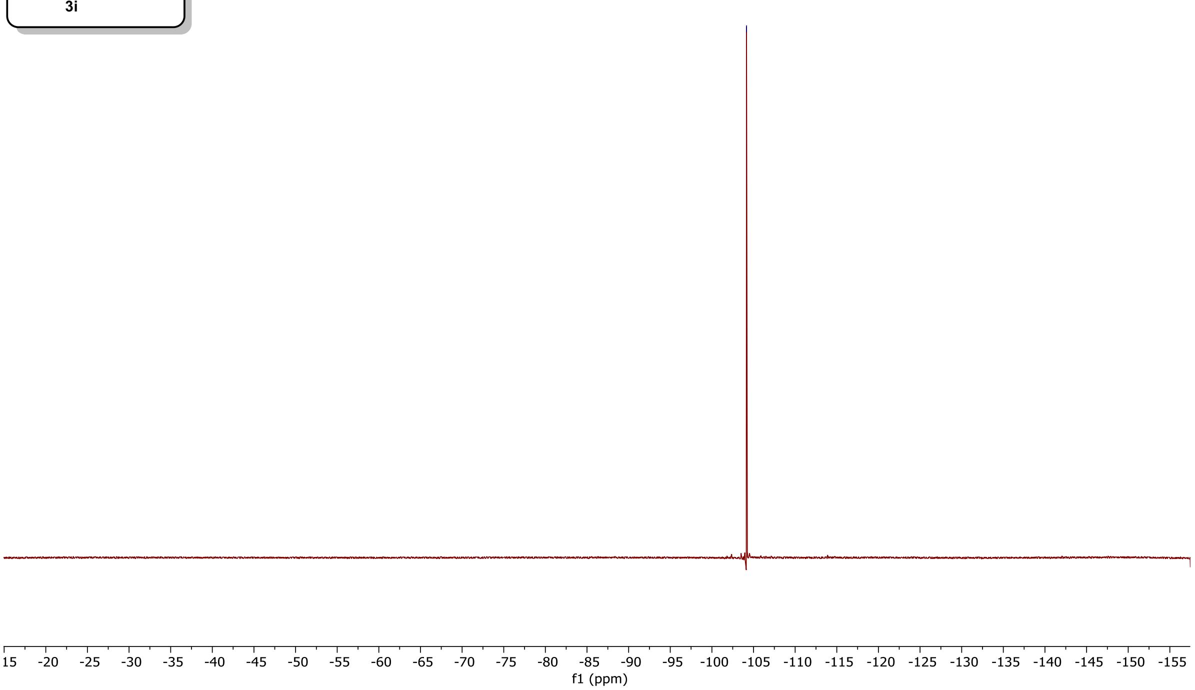


$^{19}$**F NMR** (471MHz, $CDCl_3$)

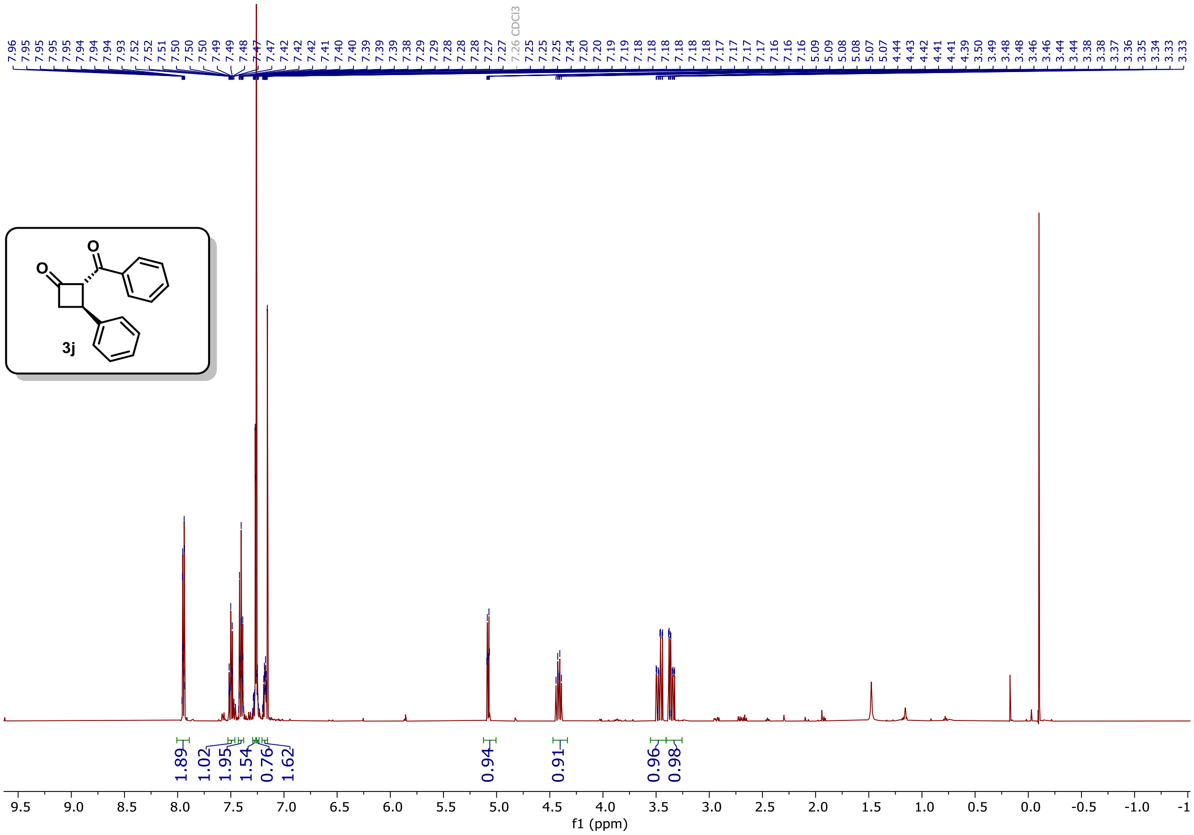


**$^{1}$H-NMR** (500 MHz, $CDCl_3$)

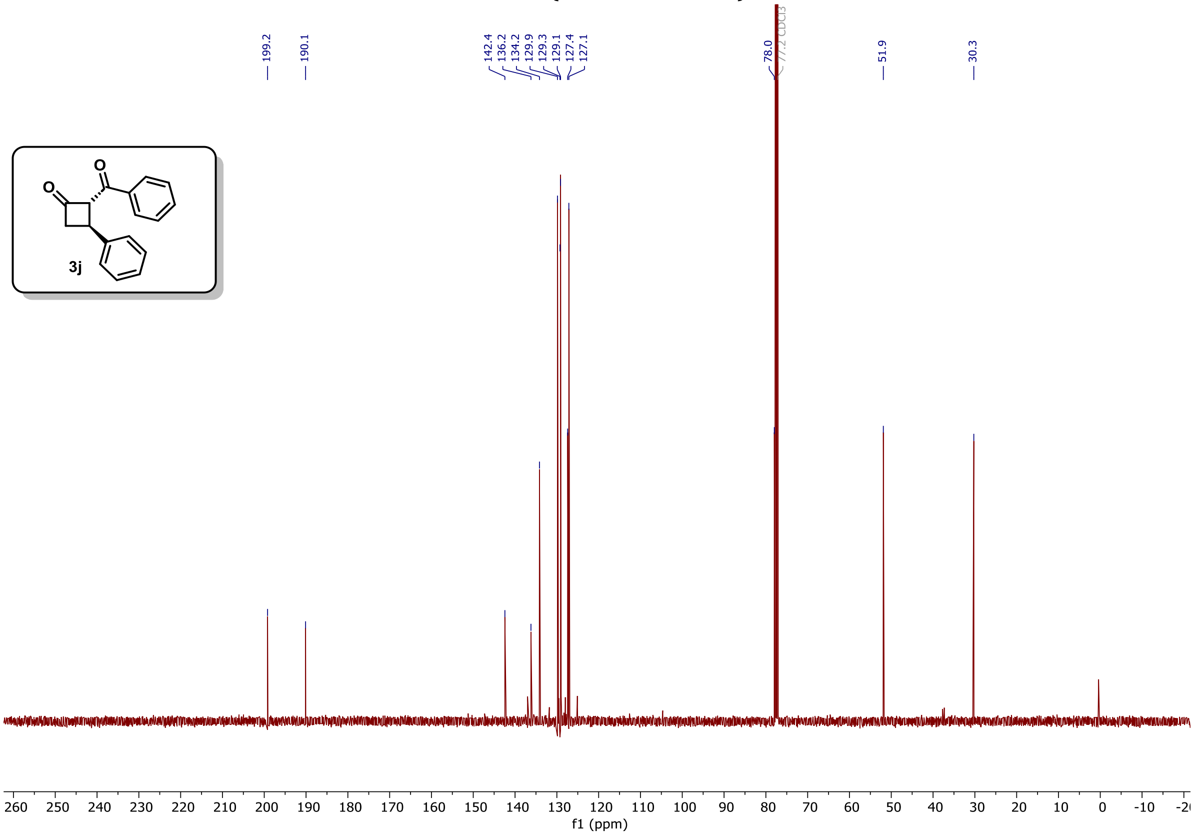


**$^{13}$C{$^{1}$H}-NMR** (126 MHz, $CDCl_3$)

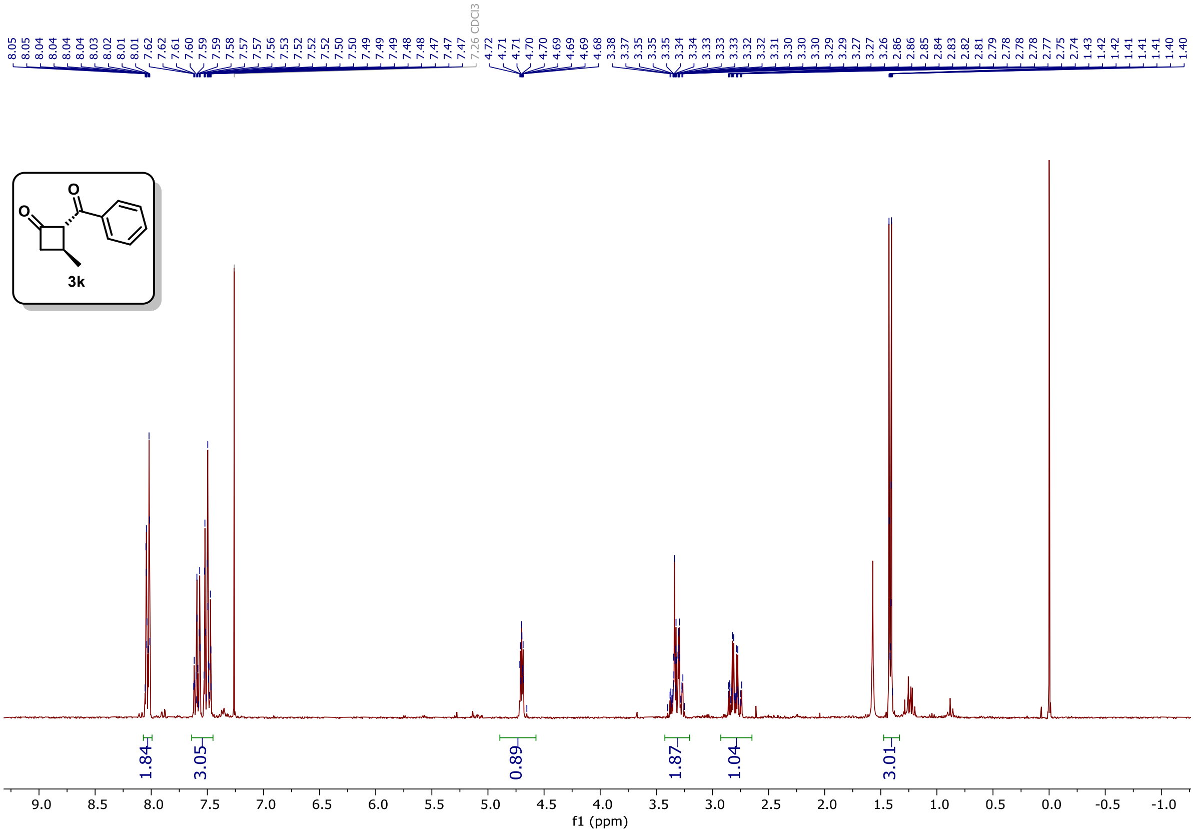


**$^{1}$H-NMR** (300 MHz, $CDCl_3$)

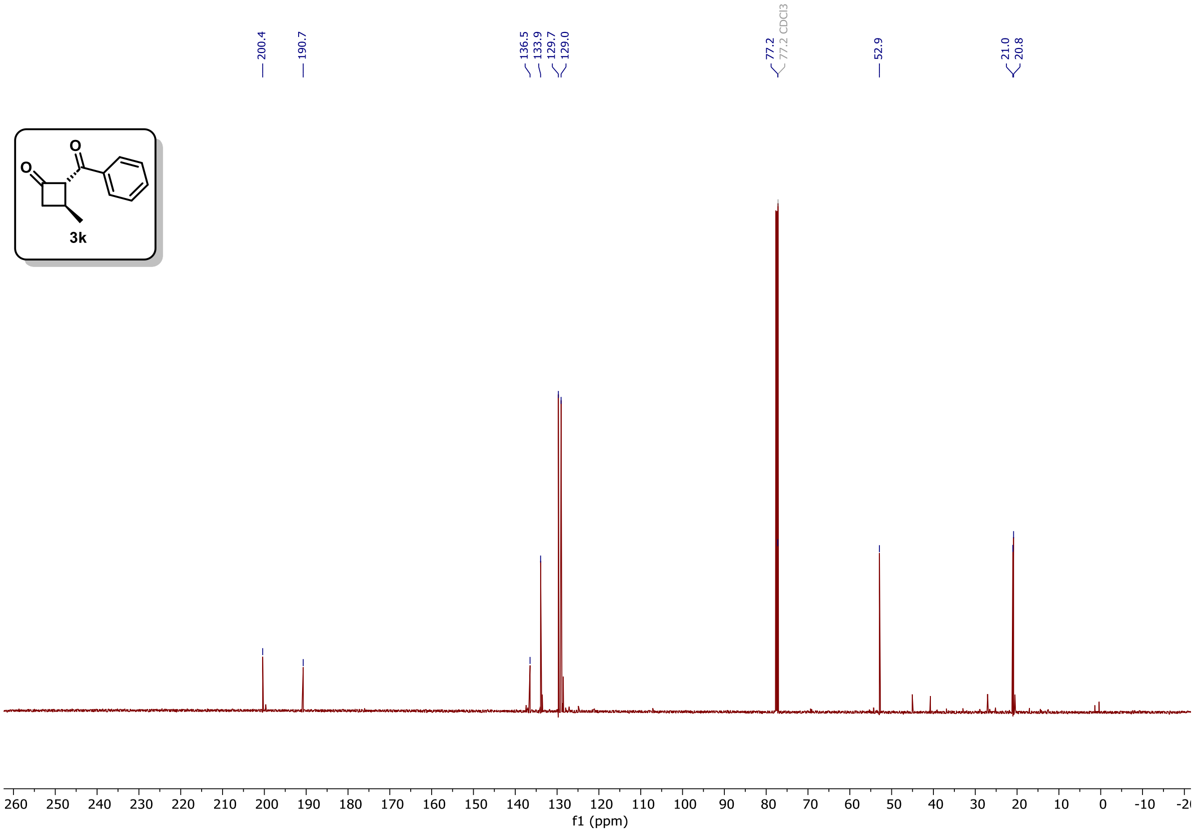


**$^{13}$C{$^{1}$H}-NMR** (126 MHz, $CDCl_3$)

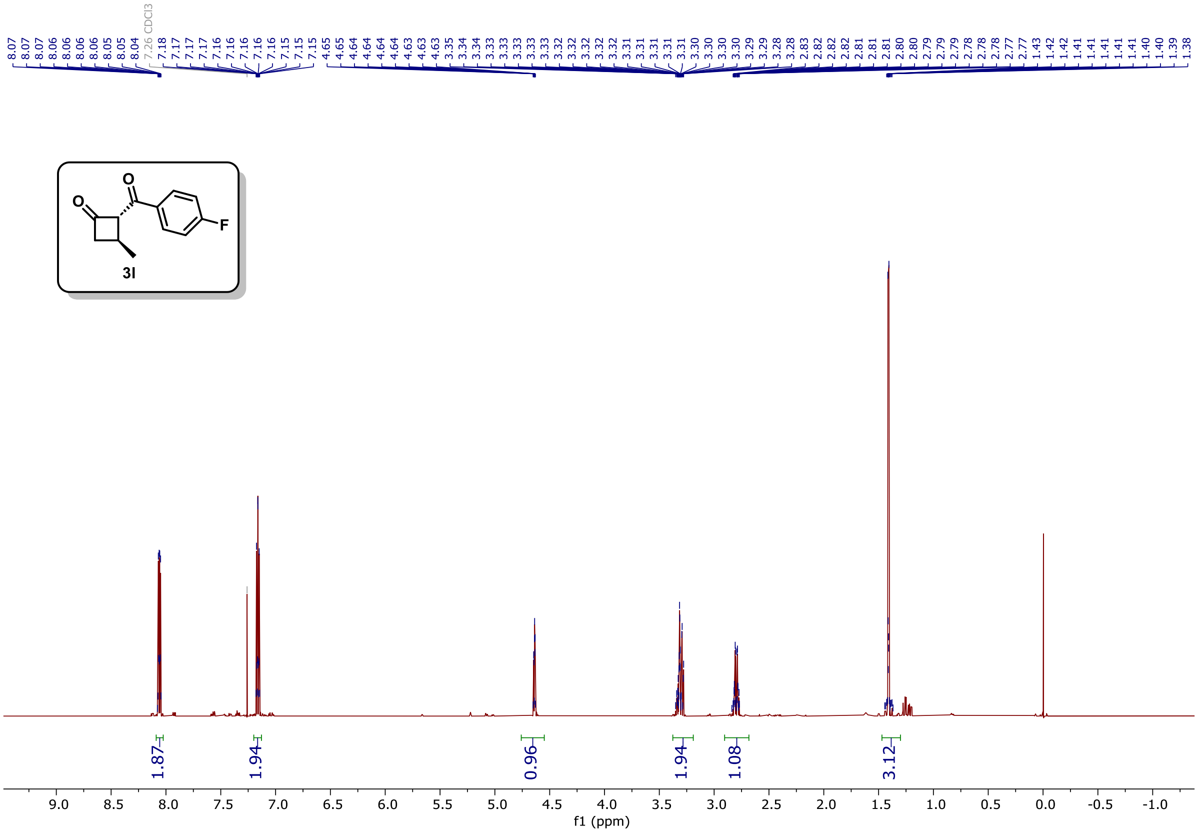


**$^{1}$H-NMR** (700 MHz, $CDCl_3$)

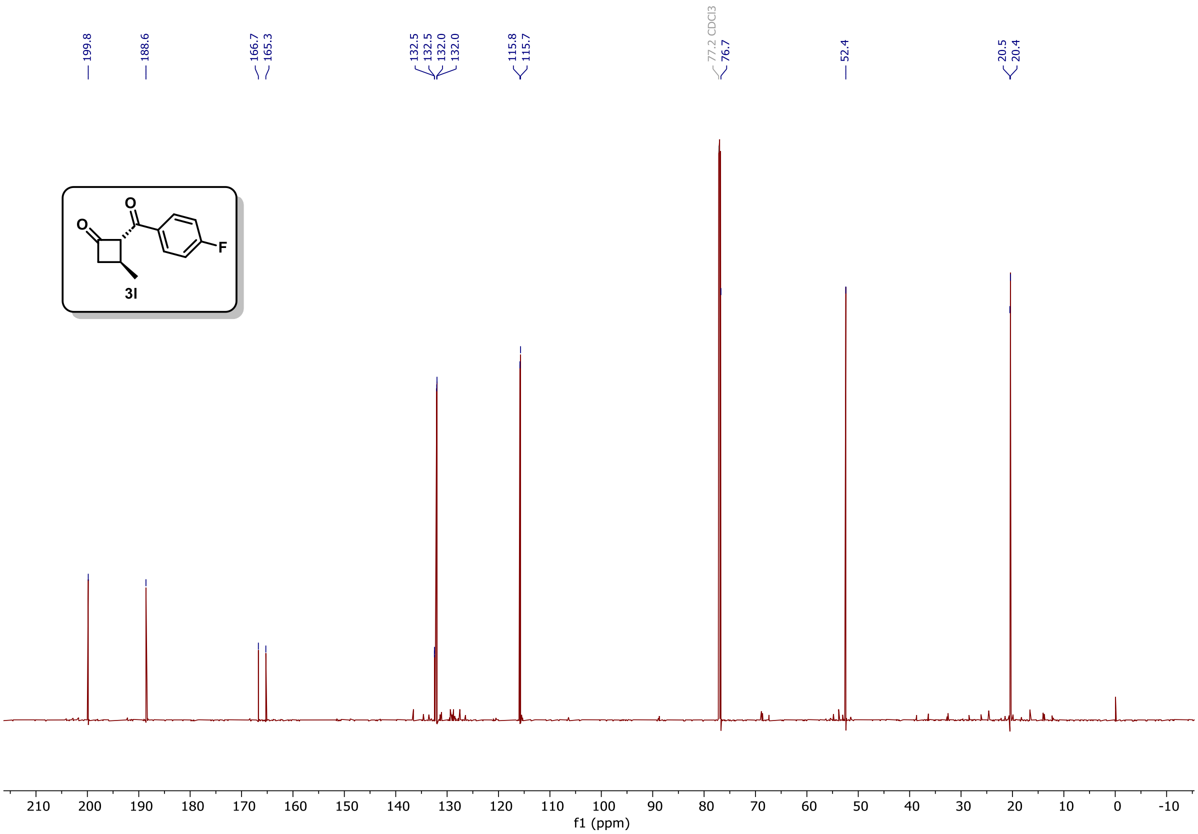


**$^{13}$C{$^{1}$H}-NMR** (176 MHz, $CDCl_3$)

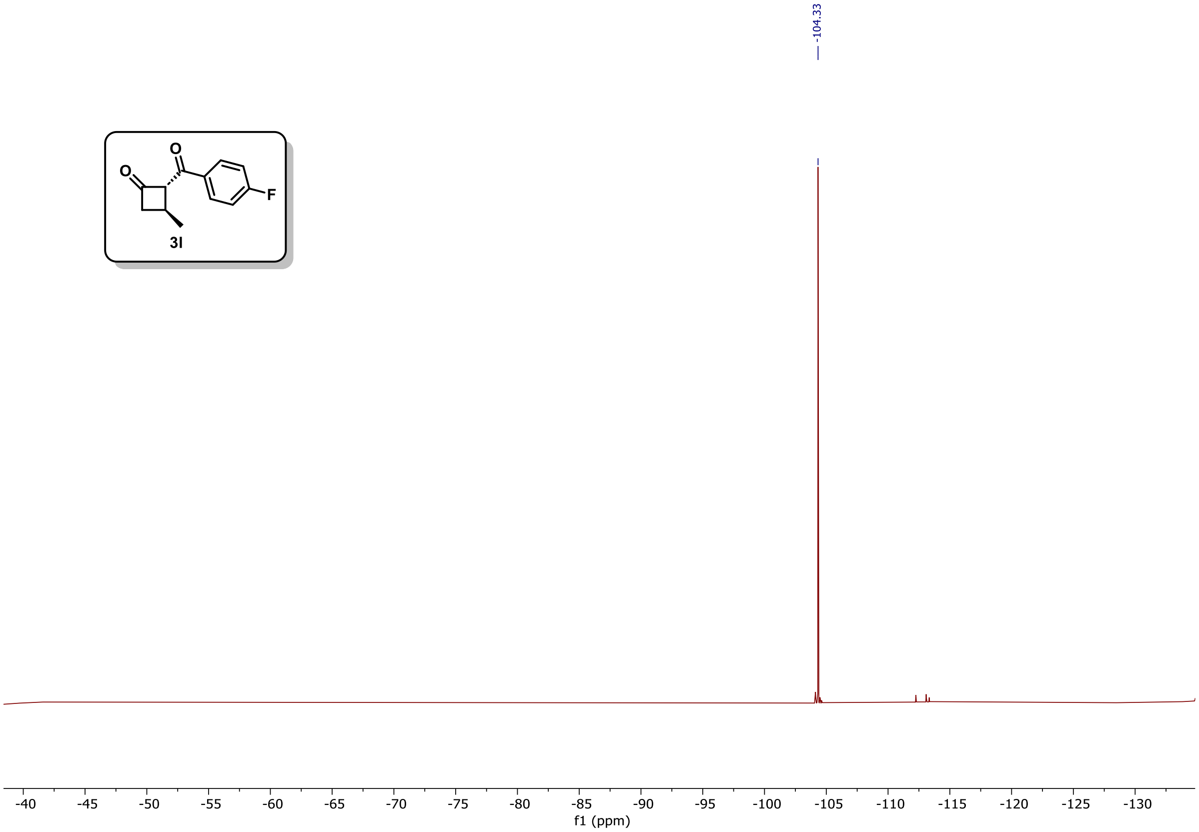


**$^{19}$F NMR** (659 MHz, $CDCl_3$)

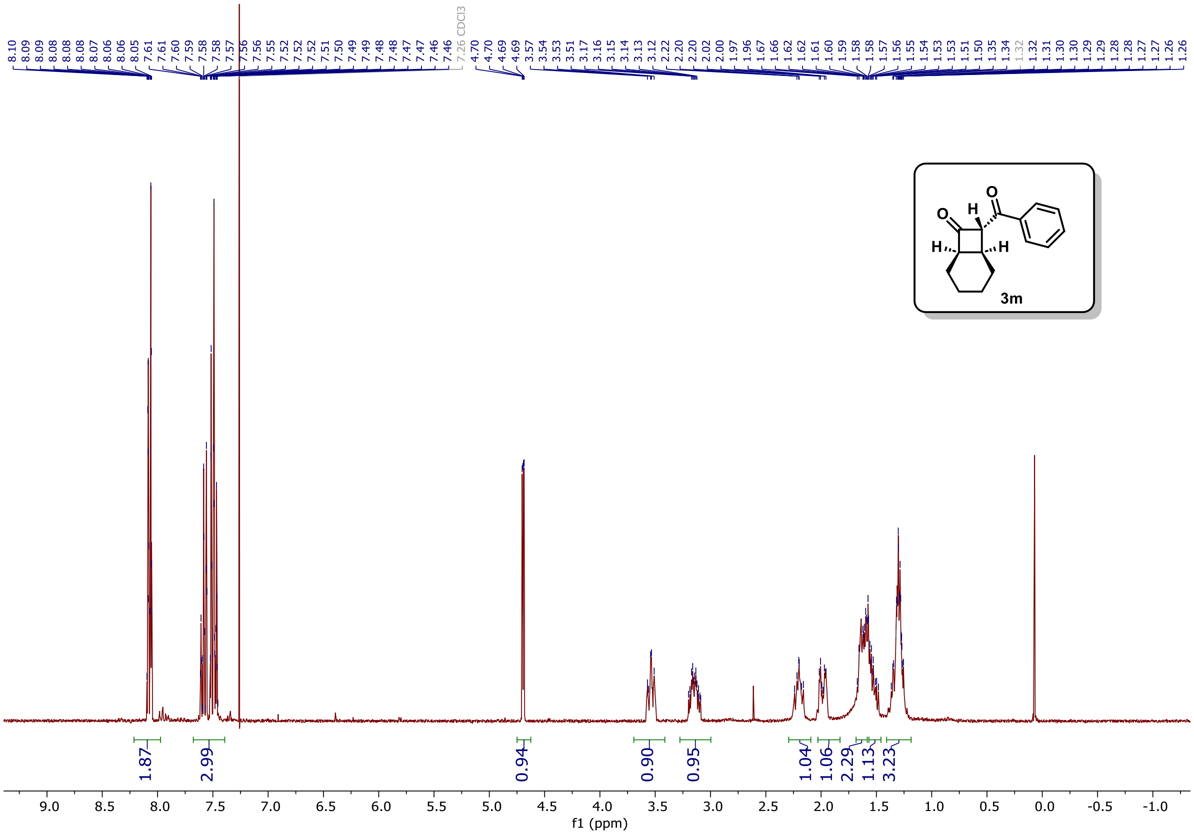


**¹H-NMR** (300 MHz, $CDCl_3$)

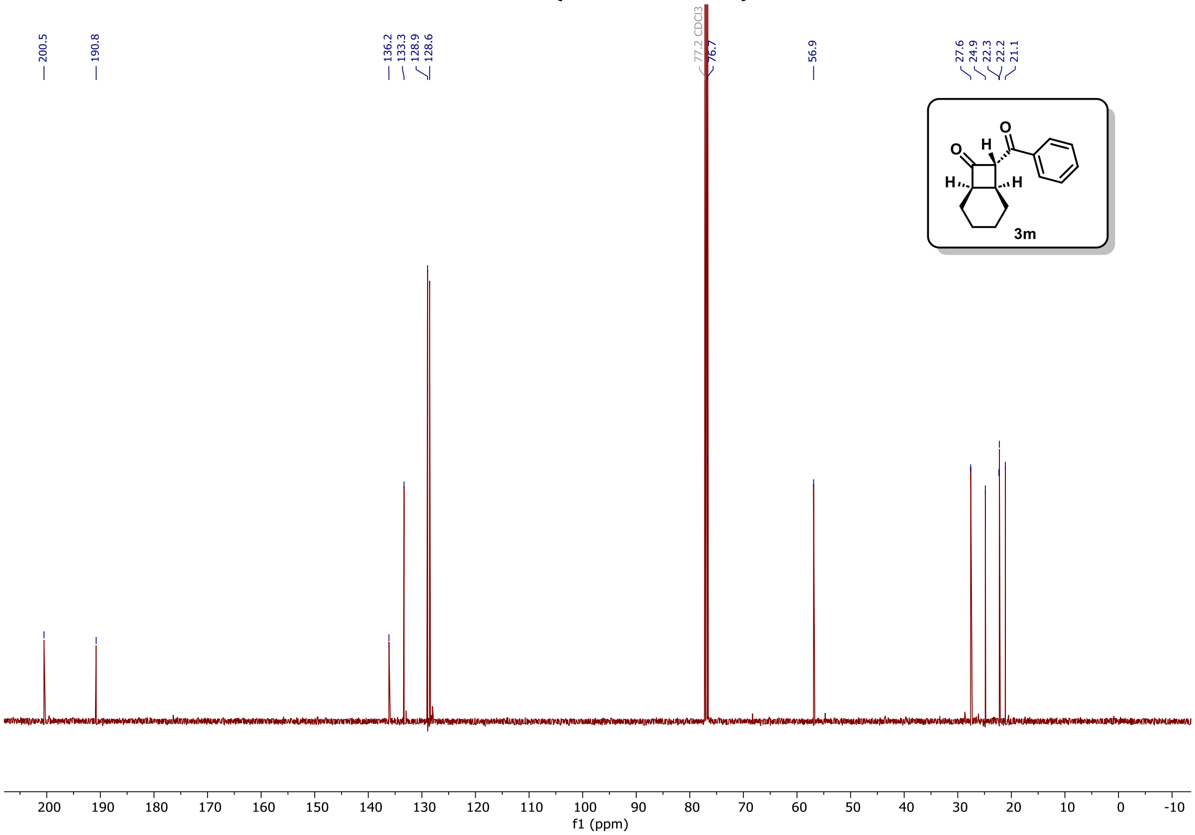


**$^{13}C\{^{1}H\}$-NMR** (126 MHz, $CDCl_3$)

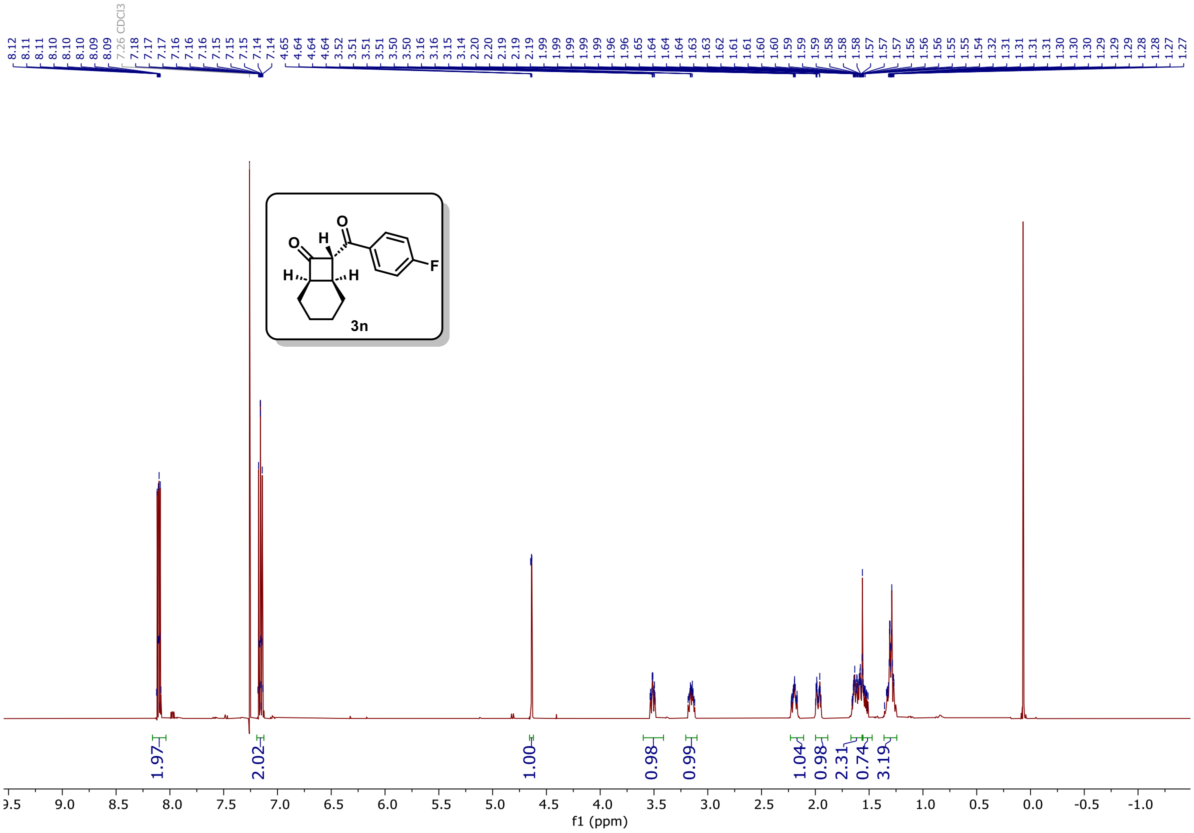


**$^{1}$H-NMR** (500 MHz, $CDCl_3$)

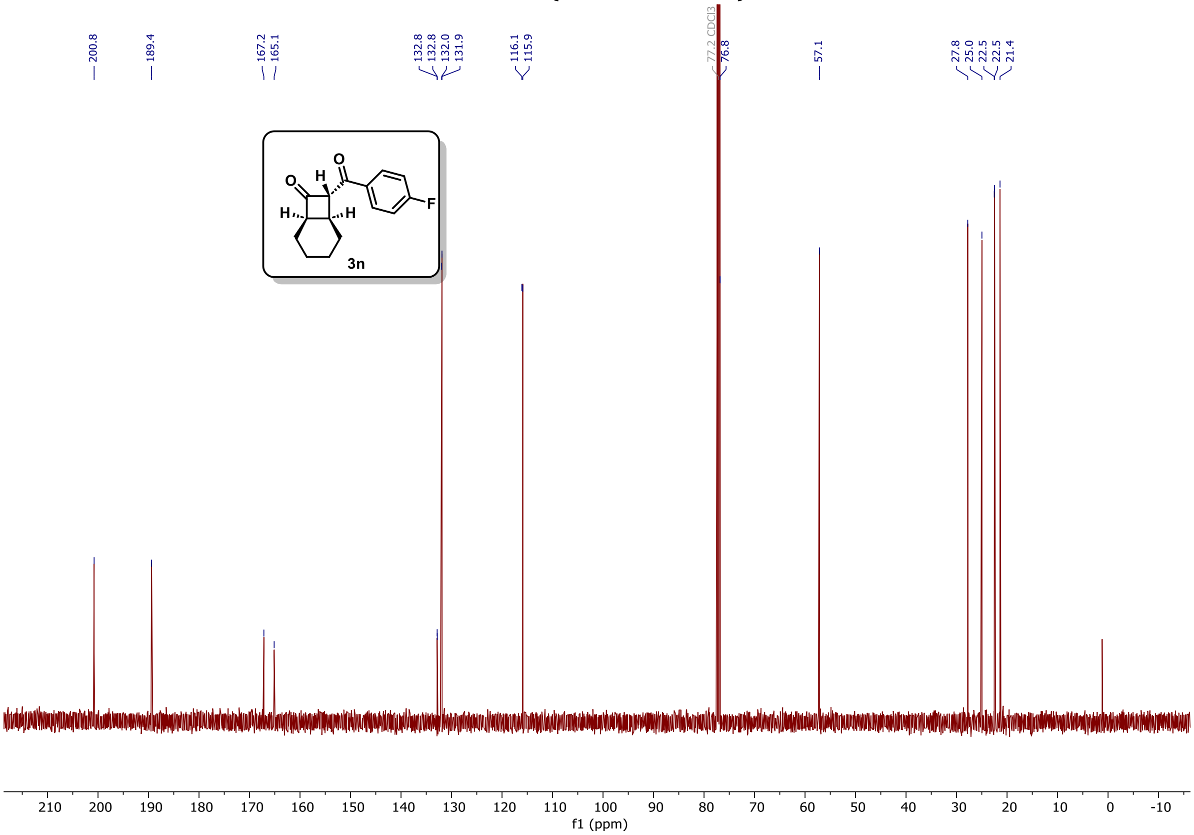


**$^{13}$C{$^{1}$H}-NMR** (126 MHz, $CDCl_3$)

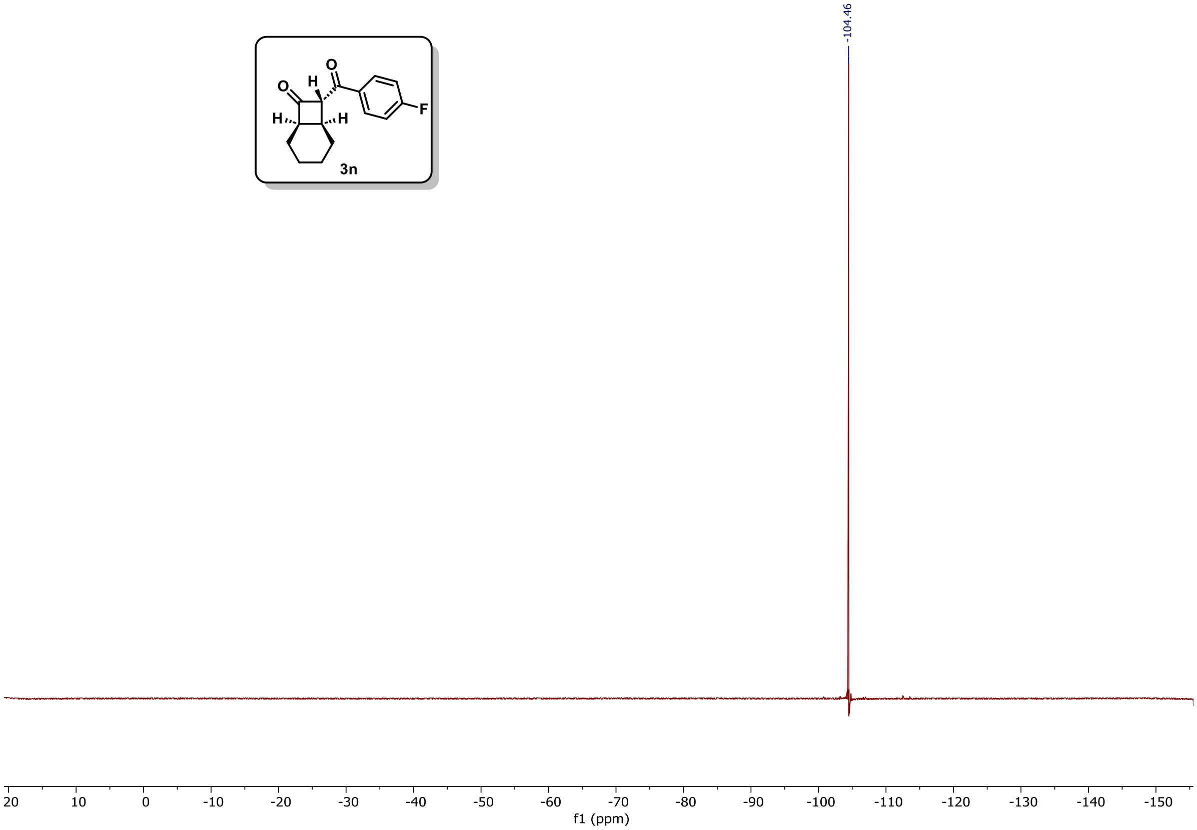


**$^{19}$F NMR** (471 MHz, $CDCl_3$)

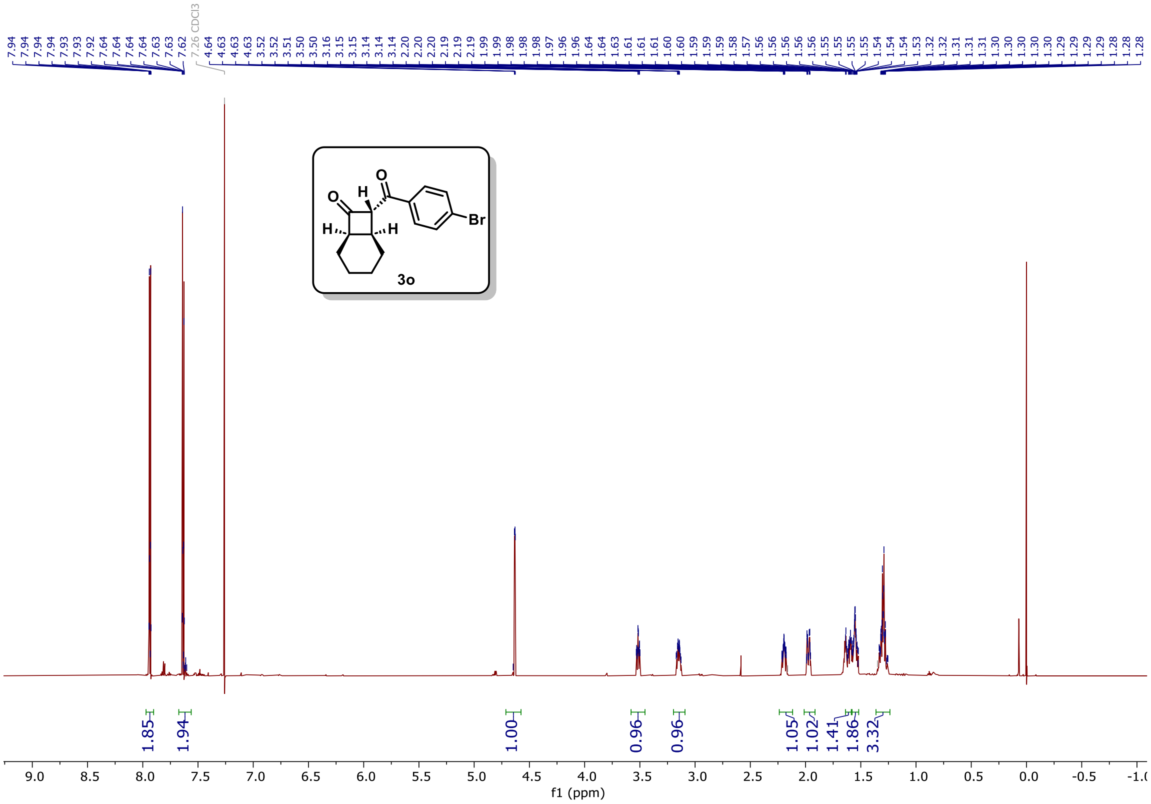


**$^1$H-NMR** (700 MHz, $CDCl_3$)

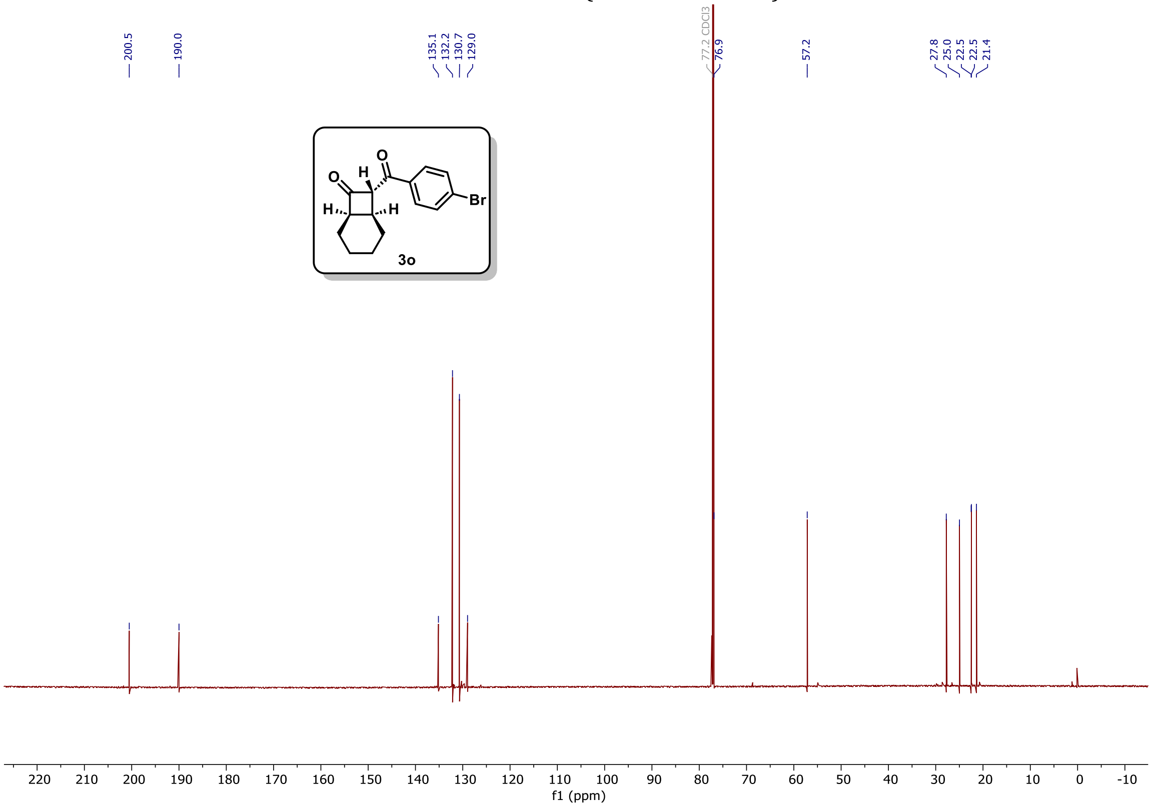


**$^{13}C\{^1H\}$-NMR** (176 MHz, $CDCl_3$)

# Follow up reactions:  NMR Spectra:

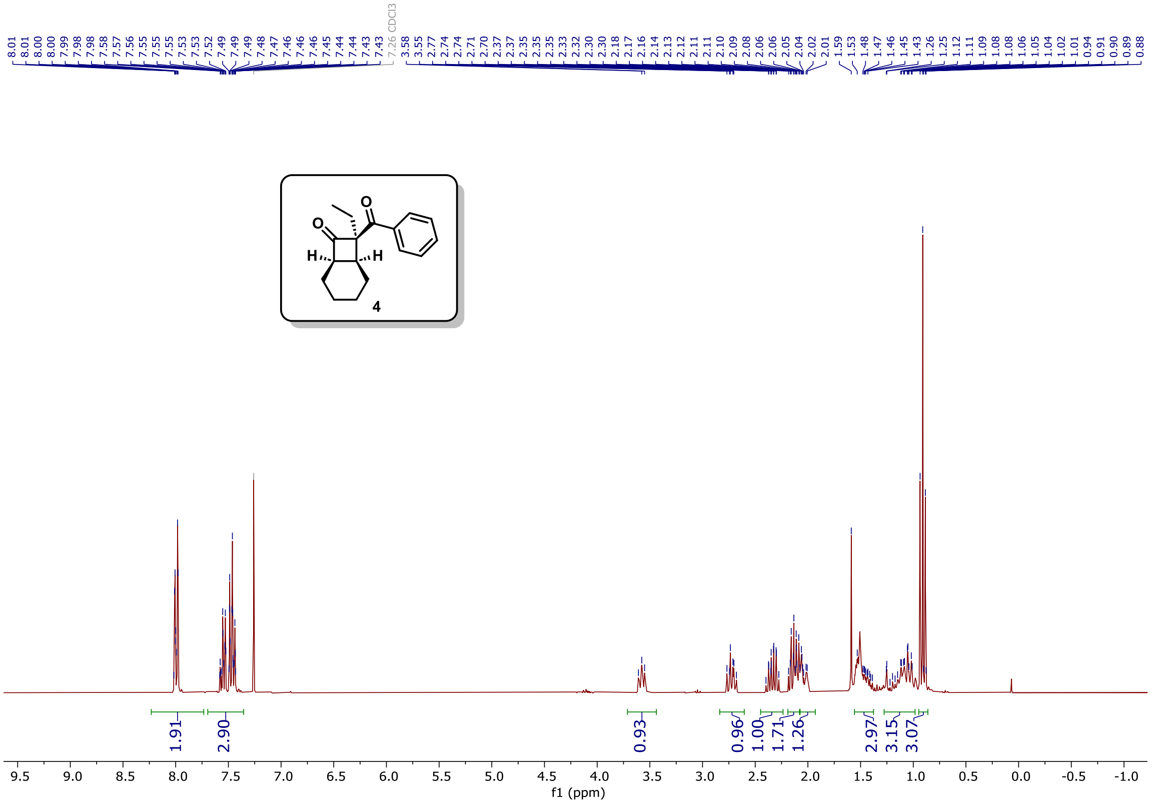

**$^1$H-NMR** (300 MHz, $CDCl_3$)

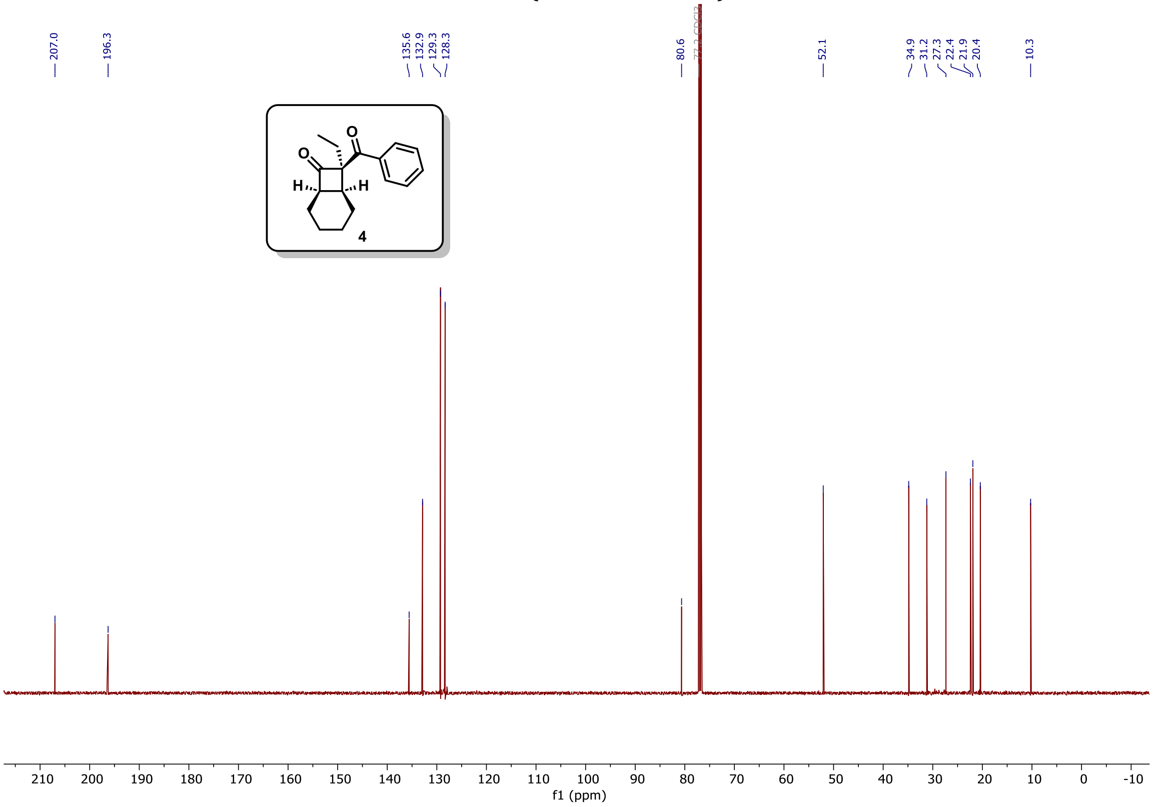


**$^{13}$C{$^1$H}-NMR** (126 MHz, $CDCl_3$)

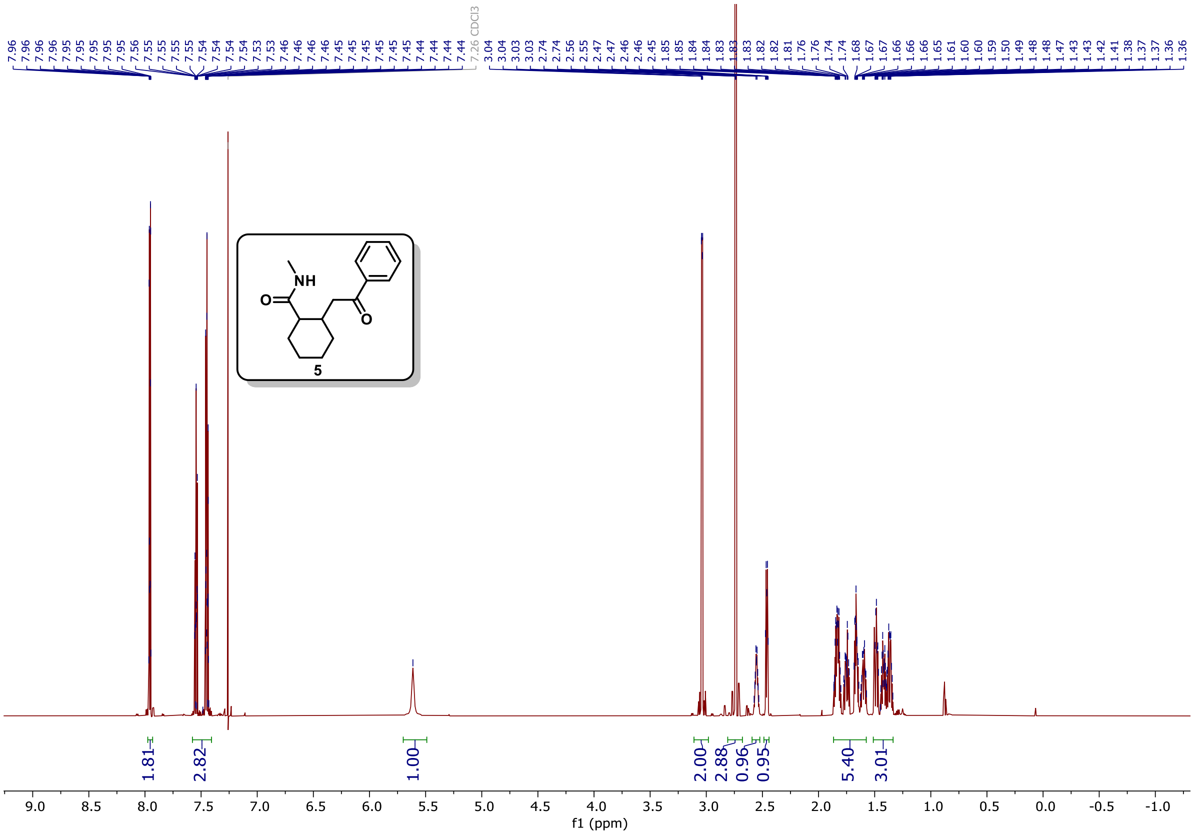


**¹H-NMR** (700 MHz, $CDCl_3$)

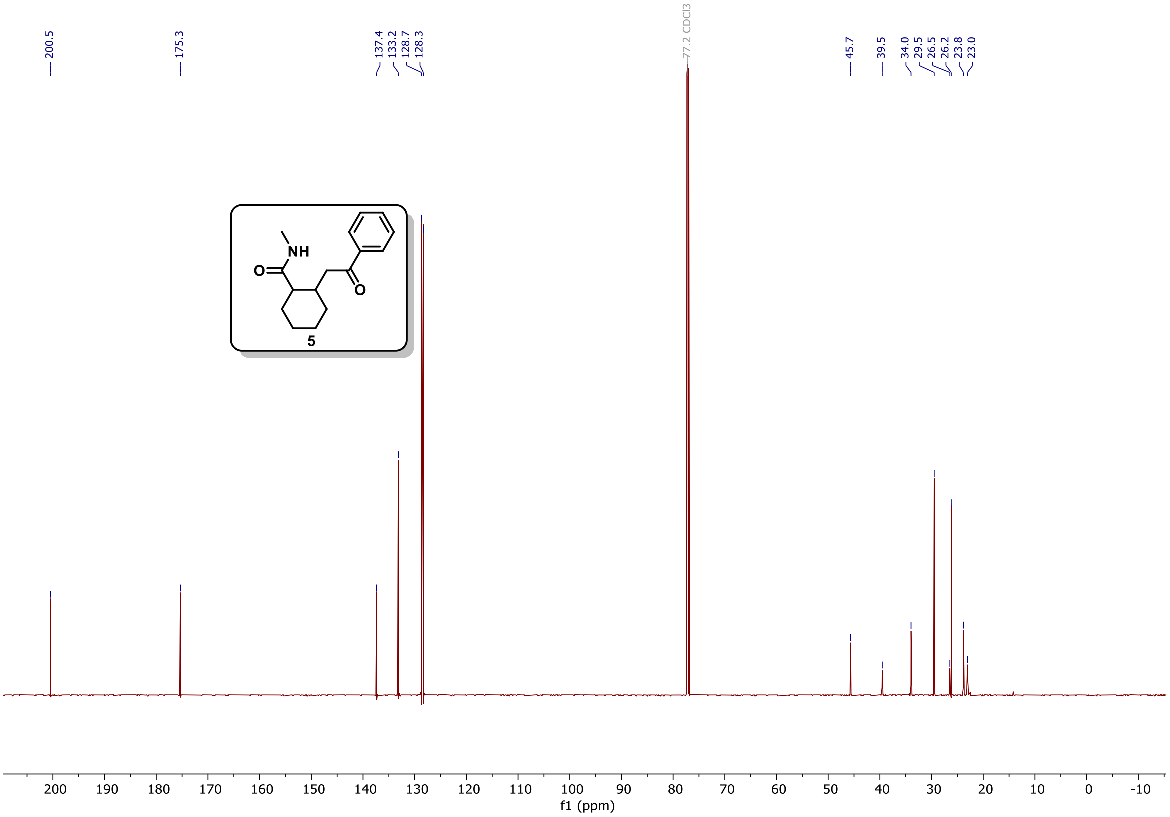


**¹³C{¹H}-NMR** (176 MHz, $CDCl_3$)

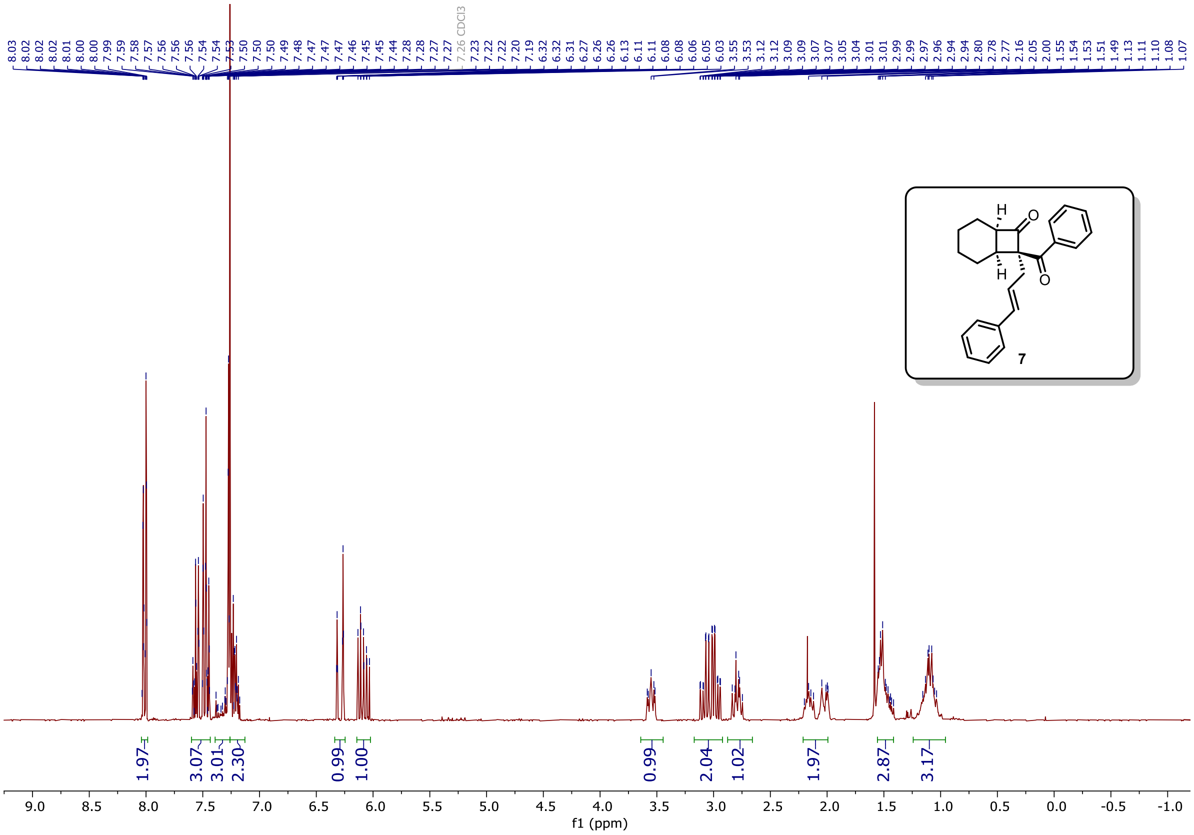


**$^{1}$H-NMR** (300 MHz, $CDCl_3$)

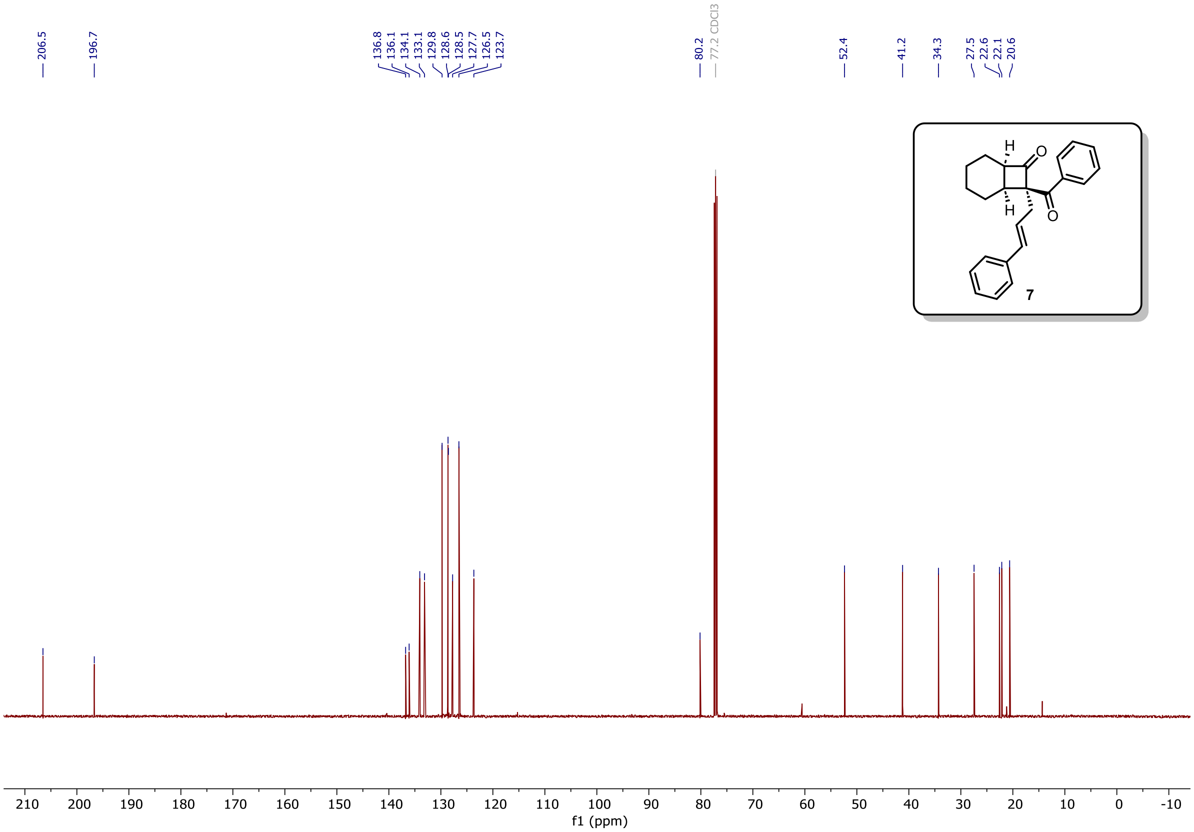


**$^{13}$C{$^{1}$H}-NMR** (126 MHz, $CDCl_3$)

# Decomposition of Cyclobutanone

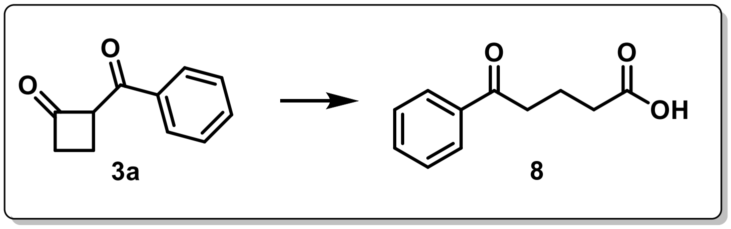


NMR data of **8**

**$^{1}$H NMR (500 MHz, $CDCl_3$):** δ 8.02 – 7.90 (m, 2H), 7.61 – 7.40 (m, 3H), 3.09 (td, *J* = 7.1, 2.2 Hz, 2H), 2.51 (td, *J* = 7.0, 2.2 Hz, 2H), 2.16 – 2.01 (m, 2H).

**$^{13}$C NMR (126 MHz, $CDCl_3$):** δ 199.5, 178.8, 136.9, 133.3, 128.8, 128.2, 37.5, 33.2, 19.2.

The 2-benzoyl cyclobutanones presented in this study show instability at room temperature in air, leading to ring-opening to the corresponding carboxylic acid over time.

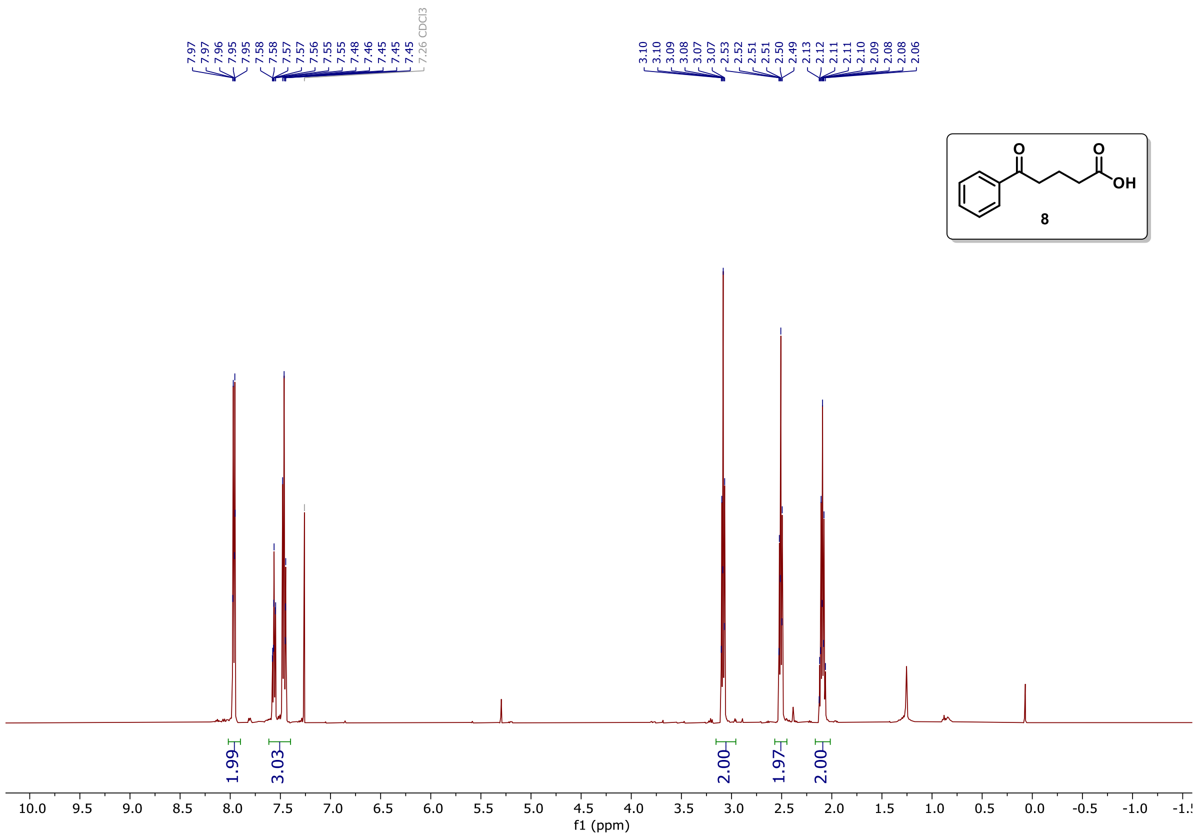


**$^1$H-NMR** (500 MHz, $CDCl_3$)

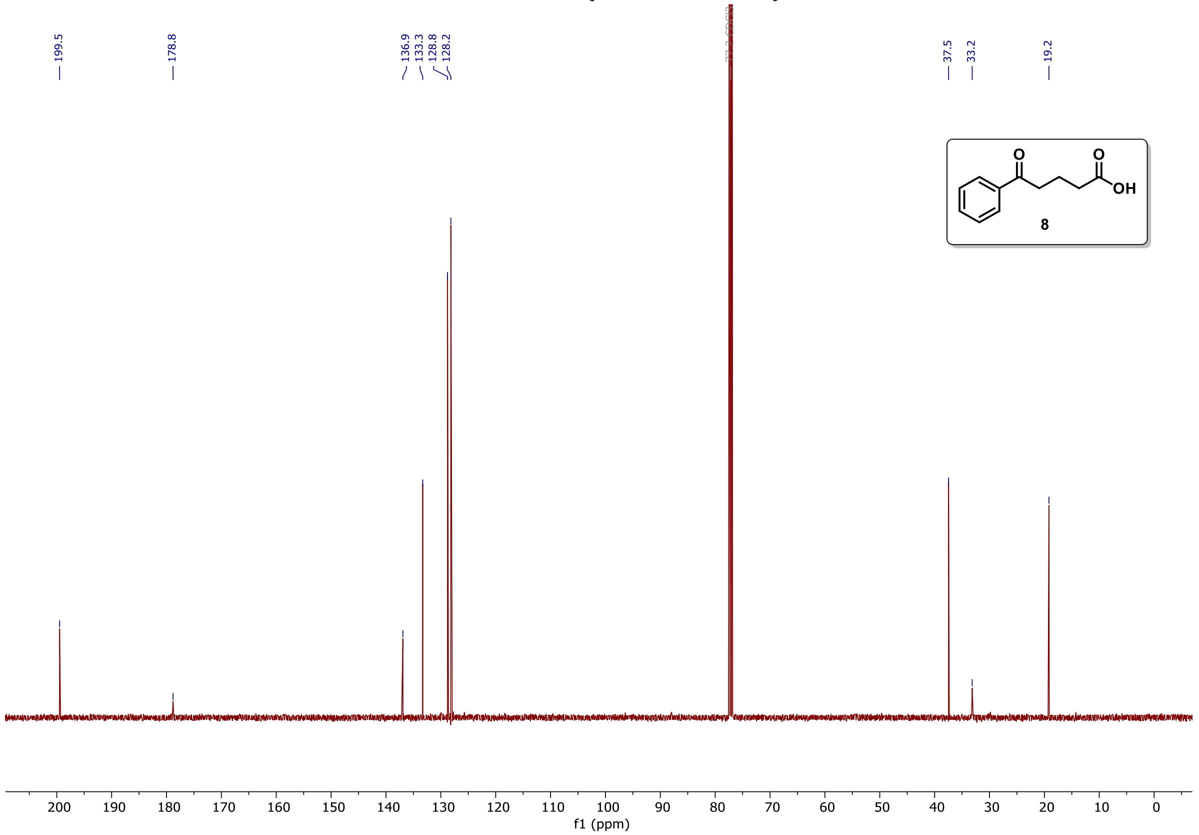


**$^{13}$C{$^1$H}-NMR** (126 MHz, $CDCl_3$)

## Stereospecificity Experiments

**Conditions for the separation of SCP 1d:**

Enantiomeric excess was determined using. Agilent 1260 Infinity with a G1311B-1260 quarternary pump. A Chiralcel OD-3 column (3 μm; 150 X 4.6 mm) was used with *n*-heptane/*i*-PrOH (95:5) w/ 0.1% AcOH eluent mixtures. Detection was conducted at 218 nm using a 1290 Infinity II detector.

flow rate: 0.5 mL · $min^{-1}$

column temperature hold at 22 °C

**Conditions for the separation of 2-benzoyl cyclobutanone 3k:**

Enantiomeric excess was determined using an Agilent 1100 series system with a G1311A quarternary pump. A Lux Cellulose-2 column (3 μm; 150 X 4.6 mm) was used with *n*-heptane/*i*-PrOH eluent (97.5:2.5) mixtures. Detection was conducted at 240 nm using a variable wavelength detector (VWD).

flow rate: 0.5 mL · $min^{-1}$

column temperature hold at 40 °C

Chromatogram of racemic SCP **1d**.

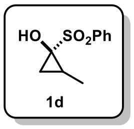


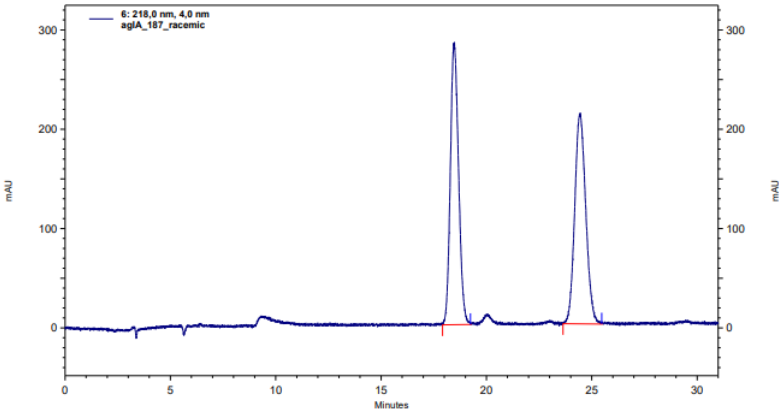


6: 218,0 nm, 4,0 nm
Results

| Peak Number | Retention Time | Area Percent | Area |
|---|---|---|---|
| 1 | 18,482 | 49,875 | 1033535424 |
| 2 | 24,455 | 50,125 | 1038701563 |
| Totals | | 100,000 | 2072236987 |

Chromatogram of enantioenriched SCP **1d**.

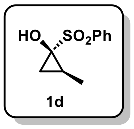


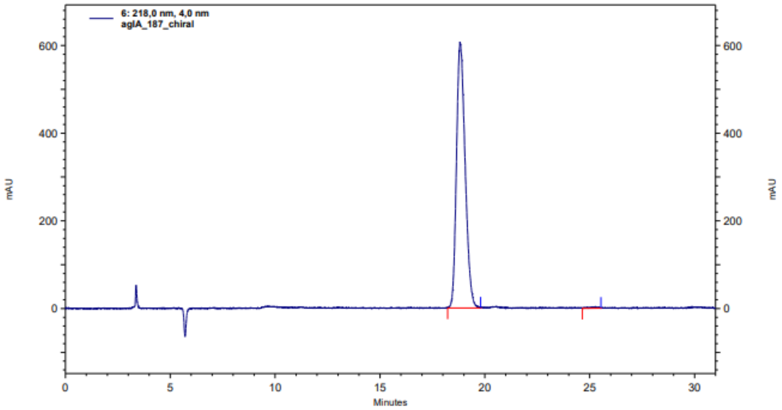


6: 218,0 nm, 4,0 nm
Results

| Peak Number | Retention Time | Area Percent | Area |
|---|---|---|---|
| 1 | 18,815 | 99,473 | 2336721448 |
| 2 | 25,245 | 0,527 | 12373090 |
| Totals | | 100,000 | 2349094538 |

Chromatogram of compound **3k** when using racemic SCP **1d** as the starting material.

| # | Time | Area | Height | Width | Area% | Symmetry |
|---|---|---|---|---|---|---|
| 1 | 13.573 | 2866.4 | 100.5 | 0.4755 | 51.059 | 1.245 |
| 2 | 15.026 | 2747.5 | 89.1 | 0.514 | 48.941 | 1.154 |

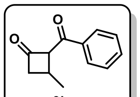
3k

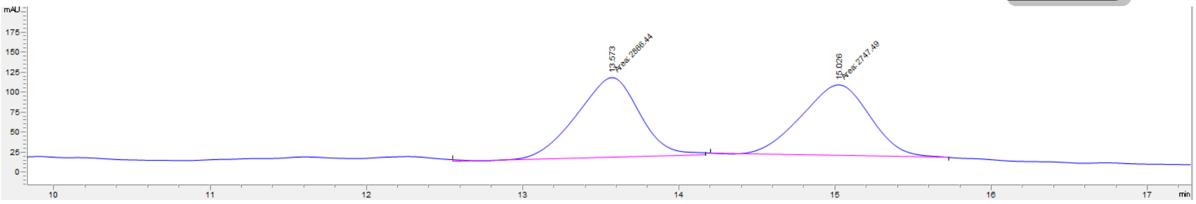

Chromatogram of compound **3k** when using enantioenriched SCP **1d** as the starting material.

| # | Time | Area | Height | Width | Area% | Symmetry |
|---|---|---|---|---|---|---|
| 1 | 13.47 | 189 | 8.3 | 0.3801 | 3.850 | 1.492 |
| 2 | 14.832 | 4720.3 | 168.1 | 0.468 | 96.150 | 1.104 |

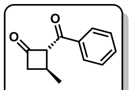
3k

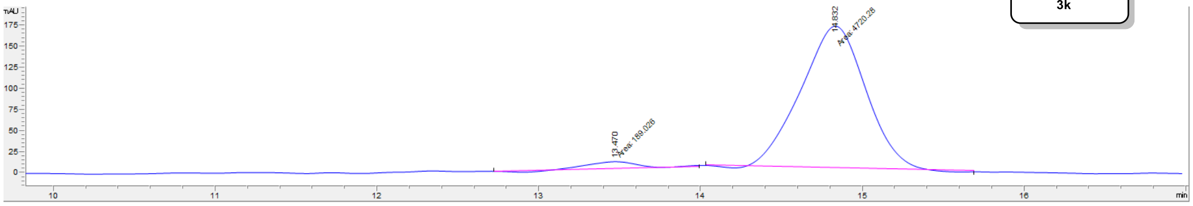

## Optimization of Reaction Conditions

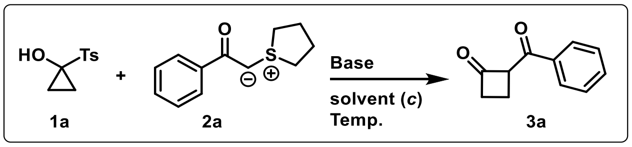


### Initial Screening

| Entry | SCP (eq.)[a] | Sulfonium Ylide (eq.) | Base (eq.) | Solvent ($c$/M) | Temp. (°C) | NMR yield of 3a (%)[d] |
|---|---|---|---|---|---|---|
| **1**[b] | **1a** (1.0) | **2a** (1.5) | KOH(1.0) | $CH_2Cl_2$ (0.05) | −78 to r.t. | – |
| **2**[b] | **1a** (1.0) | **2a** (1.5) | $NEt_3$ (1.0) | $CH_2Cl_2$ (0.05) | −78 to r.t. | – |
| **3**[b] | **1a** (1.0) | **2a** (1.5) | *t*-BuOK (1.0) | $CH_2Cl_2$ (0.05) | −78 to r.t. | – |
| **4**[b] | **1a** (1.0) | **2a** (1.5) | LiHMDS (1.0) | $CH_2Cl_2$ (0.05) | −78 to r.t. | <5 |
| **5**[b] | **1a** (1.0) | **2a** (1.5) | KHMDS (1.0) | $CH_2Cl_2$ (0.05) | −78 to r.t. | – |
| **6**[b] | **1a** (1.0) | **2a** (1.5) | NaHMDS (1.0) | $CH_2Cl_2$ (0.05) | −78 to r.t. | – |
| **7**[b] | **1a** (1.0) | **2a** (1.5) | DBU (1.0) | $CH_2Cl_2$ (0.05) | −78 to r.t. | – |
| **8**[b] | **1a** (1.0) | **2a** (1.5) | $K_3PO_4$ (1.0) | $CH_2Cl_2$ (0.05) | −78 to r.t. | 13 |
| **9**[c] | **1a** (1.0) | **2a** (1.5) | $K_3PO_4$ (1.5) | Toluene (0.05) | −78 to r.t. | 23 |
| **10**[c] | **1a** (1.0) | **2a** (1.5) | $K_3PO_4$ (1.5) | THF (0.05) | −78 to r.t. | – |
| **11**[c] | **1a** (1.0) | **2a** (1.5) | $K_3PO_4$ (1.5) | DMF (0.05) | −78 to r.t. | – |
| **12**[c] | **1a** (1.0) | **2a** (1.5) | $K_3PO_4$ (1.5) | $CH_3CN$ (0.05) | −78 to r.t. | |
| **13**[c] | **1a** (1.0) | **2a** (1.5) | $K_3PO_4$ (1.5) | DME (0.05) | −78 to r.t. | 17 |
| **14**[c] | **1a** (1.0) | **2a** (1.5) | $K_3PO_4$ (1.5) | Acetone (0.05) | −78 to r.t. | – |
| **15**[c] | **1a** (1.0) | **2a** (1.5) | $K_3PO_4$ (1.5) | EtOAc (0.05) | −78 to r.t. | – |

[a] All reactions were carried out on a 0.1 mmol scale with respect to SCP **1a**. [b] General procedure A: A stock solution of **1a** and **2a** in anhydrous solvent (0.05 M) was prepared and distributed to multiple reaction vials. After cooling to −78 °C, the respective base (solution in the indicated co-solvent or neat) was added dropwise. After warming to r.t. overnight, the crude reaction mixture was analyzed by GC/MS. [c] General procedure B: A solution of **1a**, **2a** and finely ground $K_3PO_4$ in the respective anhydrous solvent was cooled to −78 °C, and the mixture was stirred overnight while warming to r.t., the crude reaction mixture was analyzed by GC/MS. [d] Yields refer to $^1$H-NMR yield against a 1,3,5-trimethoxybenzene standard.

**Lewis Acid Screening**

| Entry | SCP (eq.)[a] | Sulfonium Ylide (eq.) | Base (eq.) | Solvent ($c$/M) | Temp. (°C) | Additive (eq.) | NMR yield of 3a (%)[b] |
|---|---|---|---|---|---|---|---|
| **1** | **1a** (1.0) | **2a** (1.5) | $K_3PO_4$ (1.5) | Toluene (0.02) | 0 to r.t. | $In(OTf)_3$(1.5) | 13 |
| **2** | **1a** (1.0) | **2a** (1.5) | $K_3PO_4$ (1.5) | Toluene (0.02) | 0 to r.t. | $ZrCl_4$ (1.5) | 34-37 |
| **3** | **1a** (1.0) | **2a** (1.5) | $K_3PO_4$ (1.5) | Toluene (0.02) | 0 to r.t. | $Sc(OTf)_3$(1.5) | – |
| **4** | **1a** (1.0) | **2a** (1.5) | $K_3PO_4$ (1.5) | Toluene (0.02) | 0 to r.t. | $Y(OTf)_3$(1.5) | – |
| **5** | **1a** (1.0) | **2a** (1.5) | $K_3PO_4$ (1.5) | Toluene (0.02) | 0 to r.t. | $ZnI_2$ (1.5) | 37 |
| **6** | **1a** (1.0) | **2a** (1.5) | $K_3PO_4$ (1.5) | Toluene (0.02) | 0 to r.t. | $ZnCl_2$ (1.5) | 31 |
| **7** | **1a** (1.0) | **2a** (1.5) | $K_3PO_4$ (1.5) | Toluene (0.02) | 0 to r.t. | $Zn(OTf)_2$ (1.5) | – |
| **8** | **1a** (1.0) | **2a** (1.5) | $K_3PO_4$ (1.5) | Toluene (0.02) | 0 to r.t. | $Cu(OTf)_2$ (1.5) | 9 |

[a] Reactions were carried out on a 0.1 mmol scale with respect to the SCP **1a**. [b] Yields refer to $^1$H-NMR yield against a 1,3,5-trimethoxybenzene standard.

**Temperature and concentration Screening**

| Entry | SCP (eq.)[a] | Sulfonium Ylide (eq.) | Base (eq.) | Solvent (*c*/M) | Temp. (°C) | Time (min/h) | Additives (eq.) | NMR yield of 3a (%)[b] |
|---|---|---|---|---|---|---|---|---|
| **1** | **1a** (1.0) | **2a** (1.5) | $K_3PO_4$ (1.5) | Toluene (0.01) | 0 to r.t. | 24 h | – | 11 |
| **2** | **1a** (1.0) | **2a** (1.5) | $K_3PO_4$ (1.5) | Toluene (0.01) | 0 to r.t. | 24 h | – | 24 |
| **3** | **1a** (1.0) | **2a** (1.5) | $K_3PO_4$ (1.5) | Toluene (0.01) | r.t. | 24 h | – | 19 |
| **4** | **1a** (1.0) | **2a** (1.5) | $K_3PO_4$ (1.5) | Toluene (0.01) | 50 | 24 h | – | 22 |
| **5** | **1a** (1.0) | **2a** (1.5) | $K_3PO_4$ (1.5) | Toluene (0.03) | 100 | 30 min | – | 52 |
| **6** | **1a** (1.0) | **2a** (1.5) | $K_3PO_4$ (1.5) | Toluene (0.01) | 100 | 30 min | $ZnI_2$(1.5) | 12 |
| **7** | **1a** (1.0) | **2a** (1.5) | $K_3PO_4$ (1.5) | Toluene (0.01) | 100 | 30 min | $ZrCl_4$(1.5) | 20 |

[a] Reactions were carried out on a 0.3 mmol scale with respect to SCP **1a**. [b] Yields refer to $^1$H-NMR yield against a 1,3,5-trimethoxybenzene standard.

# Computational Studies

## Methods

All computations were performed with the ORCA program (versions 5.0.4 or 6.0.1).[1] Structures were optimized without any geometry constraints using the M062X functional and a quadruple zeta basis set (def2-QZVP).[2] The optional empirical dispersion correction (D3zero) of Grimme et al. was applied to all computations.[3] The SCF convergence criteria were set to tight and DefGrid3 was used as integration grid in ORCA. Solvent effects were taken into account by applying the conductor-like polarizable continuum model (CPCM) with chloroform as solvent of choice. Frequency analysis was applied to confirm optimized structures as minima.

## Results

Since no keto-enol tautomerism in the $^1$H-NMR spectra of compounds **3a-n** was observed, we were interested in the energy difference between the ketone and the potential enols. Therefore, we optimized structures **(a) –(d)** and compared their energies.

| | 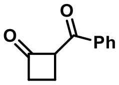 (a) | 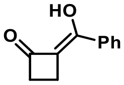 (b) | 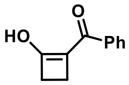 (c) | 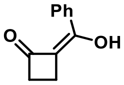 (d) |
|---|---|---|---|---|
| Electronic Energy (Eh) | -575.649121 | -575.646450 | -575.641040 | -575.639052 |
| Total thermal energy (Eh) | -575.454396 | -575.451052 | -575.445389 | -575.443966 |
| Total Enthalpy (Eh) | -575.453452 | -575.450108 | -575.444445 | -575.443021 |
| Entropy Term (kcal/mol) | 30.31 | 30.13 | 30.11 | 30.31 |
| Gibbs Free Energy (Eh) | -575.501760 | -575.498129 | -575.492436 | -575.491321 |
| ΔG (kcal/mol) | 0.0 | +2.3 | +5.9 | +6.6 |

**DFT optimized cartesian coordinates**

(a)

Lowest Freq. = 40.87 $cm^{-1}$

| | | | |
|---|---|---|---|
| C | -1.6927189 | -1.0847360 | -0.6496467 |
| C | -3.0792615 | -0.9643621 | -0.0581574 |
| C | -2.9586038 | 0.5714121 | -0.2413032 |
| C | -1.4505003 | 0.4401805 | -0.5401570 |
| O | -1.0535764 | -1.9746841 | -1.1216465 |
| C | -0.5347038 | 0.7753359 | 0.6214184 |
| O | -0.9889365 | 1.1019194 | 1.6939528 |
| C | 0.9366950 | 0.6600864 | 0.4045077 |
| C | 1.4691372 | 0.1100248 | -0.7596406 |
| C | 2.8448134 | 0.0063847 | -0.9119950 |
| C | 3.6905832 | 0.4599682 | 0.0905047 |
| C | 3.1632459 | 1.0117992 | 1.2536652 |
| C | 1.7919893 | 1.1066999 | 1.4122090 |
| H | -3.8680227 | -1.5018304 | -0.5771561 |
| H | -3.0636626 | -1.2580000 | 0.9919924 |
| H | -3.1927620 | 1.1651189 | 0.6342611 |
| H | -3.5077068 | 0.9320313 | -1.1044750 |
| H | -1.0774196 | 0.8865117 | -1.4592715 |
| H | 0.8196216 | -0.2564012 | -1.5411521 |
| H | 3.2543563 | -0.4276968 | -1.8124566 |
| H | 4.7616915 | 0.3836062 | -0.0324805 |
| H | 3.8229883 | 1.3649162 | 2.0329958 |
| H | 1.3658923 | 1.5282002 | 2.3109921 |

(b)

Lowest Freq. = 37.87 $cm^{-1}$

| | | | |
|---|---|---|---|
| C | -0.6617233 | -2.5318446 | -0.4836621 |
| C | -1.6287615 | -2.3625464 | -1.6488273 |
| C | -1.4020809 | -0.8214370 | -1.5227059 |
| C | -0.4573583 | -1.1019915 | -0.3684765 |
| O | -0.2159534 | -3.4811185 | 0.1341944 |
| C | 0.3319032 | -0.4863999 | 0.5395702 |
| C | 0.5466256 | 0.9577298 | 0.6876372 |
| O | 1.0064966 | -1.2401276 | 1.4219647 |
| C | 1.3610001 | 1.4413020 | 1.7133678 |
| C | 1.5639885 | 2.8045568 | 1.8587975 |
| C | 0.9593685 | 3.6977341 | 0.9838769 |
| C | 0.1486438 | 3.2227984 | -0.0402214 |
| C | -0.0584038 | 1.8616445 | -0.1890473 |
| H | -1.2821948 | -2.8108477 | -2.5772447 |
| H | -2.6387867 | -2.7038064 | -1.4333026 |
| H | -2.2964696 | -0.2602818 | -1.2604152 |
| H | -0.9392619 | -0.3698250 | -2.3975725 |
| H | 0.8059986 | -2.1793469 | 1.2618408 |
| H | 1.8307004 | 0.7468389 | 2.3929114 |
| H | 2.1947257 | 3.1703268 | 2.6561287 |
| H | 1.1191108 | 4.7603371 | 1.0987542 |

| | | | |
|---|---|---|---|
| H | -0.3227213 | 3.9139345 | -0.7238125 |
| H | -0.6882784 | 1.5042323 | -0.9890520 |

(c)

Lowest Freq. = 35.44 $cm^{-1}$

| | | | |
|---|---|---|---|
| C | -1.5061960 | -0.2014844 | -0.1804837 |
| C | -2.8269989 | -0.0899494 | -0.4413804 |
| C | -3.1800037 | -1.4999749 | -0.0996385 |
| C | -1.6528217 | -1.6653466 | 0.2125863 |
| O | -3.5823417 | 0.9053222 | -0.8597299 |
| C | -0.5557215 | 0.8758575 | -0.3378113 |
| O | -0.9839251 | 1.9657457 | -0.7283357 |
| C | 0.8943506 | 0.6976120 | -0.0498049 |
| C | 1.4047875 | -0.5004565 | 0.4462930 |
| C | 2.7626269 | -0.6262992 | 0.7048901 |
| C | 3.6166930 | 0.4416346 | 0.4670748 |
| C | 3.1129432 | 1.6398871 | -0.0289502 |
| C | 1.7587627 | 1.7670239 | -0.2847184 |
| H | -3.8416045 | -1.6137703 | 0.7565274 |
| H | -3.5480721 | -2.0957035 | -0.9322873 |
| H | -1.1473270 | -2.3804725 | -0.4328251 |
| H | -1.4422727 | -1.8986986 | 1.2540902 |
| H | -2.9880408 | 1.6699705 | -0.9767146 |
| H | 0.7501863 | -1.3368940 | 0.6365429 |
| H | 3.1521305 | -1.5570643 | 1.0911979 |
| H | 4.6741139 | 0.3422064 | 0.6676219 |
| H | 3.7780603 | 2.4710103 | -0.2142666 |
| H | 1.3516706 | 2.6908441 | -0.6688778 |

(d)

Lowest Freq. = 51.73 $cm^{-1}$

| | | | |
|---|---|---|---|
| C | -1.7421042 | -1.3286084 | 0.6053709 |
| C | -3.2671731 | -1.1991266 | 0.5305677 |
| C | -3.0701954 | 0.1135142 | -0.2791244 |
| C | -1.5723648 | -0.0401023 | -0.1065884 |
| O | -1.0179368 | -2.1727686 | 1.0566195 |
| C | -0.5554228 | 0.7983974 | -0.3565715 |
| C | 0.8673008 | 0.5436045 | -0.0688170 |
| O | -0.8676932 | 2.0092603 | -0.8772753 |
| C | 1.3990455 | -0.7432535 | -0.1464842 |
| C | 2.7435682 | -0.9546489 | 0.1120596 |
| C | 3.5710844 | 0.1076589 | 0.4525464 |
| C | 3.0473434 | 1.3901201 | 0.5403088 |
| C | 1.7038420 | 1.6074086 | 0.2793678 |
| H | -3.7563841 | -2.0215281 | 0.0102992 |
| H | -3.7261151 | -1.0690208 | 1.5102135 |
| H | -3.4941121 | 1.0059837 | 0.1791309 |
| H | -3.4080358 | 0.0551662 | -1.3135458 |
| H | -0.0681470 | 2.4385210 | -1.2018137 |
| H | 0.7558259 | -1.5722723 | -0.4037456 |

| | | | |
|---|---|---|---|
| H | 3.1476746 | -1.9560372 | 0.0446337 |
| H | 4.6204482 | -0.0647623 | 0.6531764 |
| H | 3.6816032 | 2.2207867 | 0.8206585 |
| H | 1.2967202 | 2.6061684 | 0.3888700 |

## Crystal Structure Determinations

Crystals of **3o** were obtained at room temperature by slow solvent evaporation from a solution of the compound dissolved in chloroform. A colourless, block-shaped crystal was mounted on a MiTeGen micromount with perfluoroether oil. Data were collected from a shock-cooled single crystal at 100(2) K on a Bruker D8 VENTURE dual wavelength Mo/Cu three-circle diffractometer with a microfocus sealed X-ray tube using a mirror optics as monochromator and a Bruker PHOTON III detector. The diffractometer was equipped with an Oxford Cryostream 800 low temperature device and used Mo$K_\alpha$ radiation (λ = 0.71073 Å). All data were integrated with SAINT V8.41 and a multi-scan absorption correction using SADABS 2016/2 was applied.[4,5] The structure was solved by direct methods with SHELXT 2018/2 and refined by full-matrix least-squares methods against $F^2$ using SHELXL-2019/2.[6,7] All non-hydrogen atoms were refined with anisotropic displacement parameters. All hydrogen atoms were refined isotropic on calculated positions using a riding model with their $U_{iso}$ values constrained to 1.5 times the $U_{eq}$ of their pivot atoms for terminal sp$^3$ carbon atoms and 1.2 times for all other carbon atoms.

Crystallographic data for the structures reported in **Table S1** have been deposited with the Cambridge Crystallographic Data Centre.[8] CCDC 2440974 contain the supplementary crystallographic data for this paper. Additionally, ellipsoid plots are presented as **Fig. S1-1**. These data can be obtained free of charge from The Cambridge Crystallographic Data Centre via www.ccdc.cam.ac.uk/structures. This report and the CIF file were generated using FinalCif.[9]

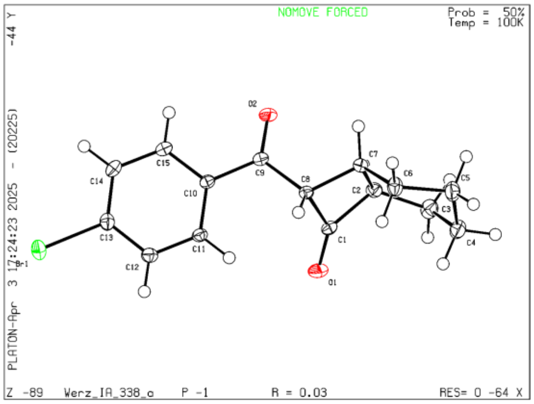


**Fig. S1-1** The molecule of compound **3o** in the crystal. Ellipsoids correspond to 50% probability levels

## Table S1. Crystallographic data and structure refinement details for compound 3o.

| | |
|---|---|
| CCDC number | 2440974 |
| Empirical formula | $C_{15}H_{15}BrO_2$ |
| Formula weight | 307.18 |
| Crystal size [mm$^3$] | 0.150×0.395×0.810 |
| Crystal shape | block |
| Crystal system | triclinic |
| Space group (number) | $P\overline{1}$ (2) |
| *a* [Å] | 5.274(2) |
| *b* [Å] | 9.933(4) |
| *c* [Å] | 13.724(6) |
| α [°] | 107.563(6) |
| β [°] | 98.947(10) |
| γ [°] | 100.88(2) |
| Volume [Å$^3$] | 655.7(5) |
| *Z* | 2 |
| Radiation | Mo$K_\alpha$ (λ=0.71073 Å) |
| $\rho_{calc}$ [gcm$^{-3}$] | 1.556 |
| $\mu$ [mm$^{-1}$] | 3.126 |
| *F*(000) | 312 |
| 2θ range [°] | 3.20 to 62.91 (0.68 Å) |
| Reflections collected | 34354 |
| Independent reflections | 4354<br>$R_{int}$ = 0.0420<br>$R_{sigma}$ = 0.0250 |
| Data / Restraints / Parameters | 4354 / 0 / 163 |
| Final *R* indexes [$I \geq 2\sigma(I)$] | $R_1$ = 0.0257<br>w$R_2$ = 0.0625 |
| Final *R* indexes [all data] | $R_1$ = 0.0324<br>w$R_2$ = 0.0648 |
| Largest peak/hole [eÅ$^{-3}$] | 0.47/−0.37 |
| Goodness-of-fit on $F^2$ | 1.029 |

# References

(1) (a) Neese, F.; Wennmohs, F.; Becker, U.; Riplinger, C. The ORCA quantum chemistry program package. *J. Chem. Phys.* **2020**, *152*, 224108. (b) Neese, F. Software update: The ORCA program system—Version 5.0. *Wiley Interdiscip. Rev. Comput. Mol. Sci.* **2022**, *12*, e1606.

(2) (a) Weigend, F.; Ahlrichs, R. Balanced basis sets of split valence, triple zeta valence and quadruple zeta valence quality for H to Rn: Design and assessment of accuracy. *Phys. Chem. Chem. Phys.* **2005**, *7*, 3297. (b) Walker, M.; Harvey, A. J. A.; Sen, A.; Dessent, C. E. H. Performance of M06, M06-2X, and M06-HF Density Functionals for Conformationally Flexible Anionic Clusters: M06 Functionals Perform Better than B3LYP for a Model System with Dispersion and Ionic Hydrogen-Bonding Interactions. *J. Phys. Chem. A* **2013**, *117*, 12590.

(3) (a) Smith, D. G. A.; Burns, L. A.; Patkowski, K.; Sherrill, C. D. Revised Damping Parameters for the D3 Dispersion Correction to Density Functional Theory. *J. Phys. Chem. Lett.* **2016**, 7, 2197.
(b) Grimme, S.; Antony, J.; Ehrlich, S.; Krieg, H. A consistent and accurate ab initio parametrization of density functional dispersion correction (DFT-D) for the 94 elements H-Pu *J. Chem. Phys.* **2010**, *132*, 154104.

(4) Bruker, *SAINT*, *V8.41*, Bruker AXS Inc., Madison, Wisconsin, USA.

(5) Krause, L.; Herbst-Irmer, R.; Sheldrick, G. M.; Stalke, D. Comparison of silver and molybdenum microfocus X-ray sources for single-crystal structure determination. *J. Appl. Cryst.* **2015**, *48*, 3–10.

(6) Sheldrick, G. M. SHELXT - integrated space-group and crystal-structure determination. *Acta Cryst.* **2015**, *A71*, 3–8.

(7) Sheldrick, G. M. Crystal structure refinement with SHELXL. *Acta Cryst.* **2015**, *C71*, 3–8.

(8) Groom, C. R.; Bruno, I. J.; Lightfoot, M. P.; Ward, S. C. The Cambridge Structural Database. *Acta Cryst.* **2016**, *B72*, 171–179.

(9) D. Kratzert, *FinalCif*, *V150*, https://dkratzert.de/finalcif.html.
